# Supplementary material for: Design, Synthesis and Biological Evaluation of 6-(Imidazo[1,2-a]pyridin-6-yl)quinazoline Derivatives as Anticancer Agents via PI3Kα Inhibition
Source: Int J Mol Sci. 2023 Apr 6;24(7):6851. doi: 10.3390/ijms24076851 (PMC10095550; doi:10.3390/ijms24076851)
Supplement: Supplementary file 1 [file ijms-24-06851-s001.zip › ijms-2253333-supplementary.pdf]

Supporting Information

Design, synthesis and biological evaluation of 6-(imidazo[1,2-a]pyridin-6-yl)quinazoline derivatives as anticancer agents via PI3Kα inhibition

Mei Li [1,2†], Daoping Wang [1,2†], Qing Li [1,2], Fang Luo [1,2], Ting Zhong [1,2], Hongshan Wu [1,2], Liang Xiong [1,2], Meitao Yuan [1,2], Mingzhi Su [1,2], Yanhua Fan [1,2 *]

[1] State Key Laboratory for Functions and Applications of Medicinal Plants, Guizhou Medical University, Guiyang 550014, China

[2] The Key Laboratory of Chemistry for Natural Products of Guizhou Province and Chinese Academy of Sciences, Guiyang 550014, China

[†] These authors contributed equally to this work.

*Corresponding author: State Key Laboratory for Functions and Applications of Medicinal Plants, Guizhou Medical University, Email address: fanyhkyem@163.com (Y.H. Fan).

Table of Contents

1. Supplementary diagram of 3D spheroid cell inhibition assay……………….2
2. Synthesis of compounds 10a-10u………………………………………………2
2.1. General experimental protocol for the preparation of intermediates 5a-5q
2.2. General experimental protocol for the preparation of intermediates 7a-7o
2.3. General experimental protocol for the preparation of compounds 10a-10u
3. Synthesis of compounds 13a-13k……………………………………………...21
3.1. General experimental protocol for the preparation of intermediates 12a-12c
3.2. General experimental protocol for preparation of compounds 13a-13k
4. ^{1}H NMR and ^{13}C NMR Spectrum of the compounds 10a-10u ……..………27
5. ^{1}H NMR and ^{13}C NMR Spectrum of the compounds 13a-13k………………63

1. Supplementary diagram of 3D spheroid cell inhibition assay

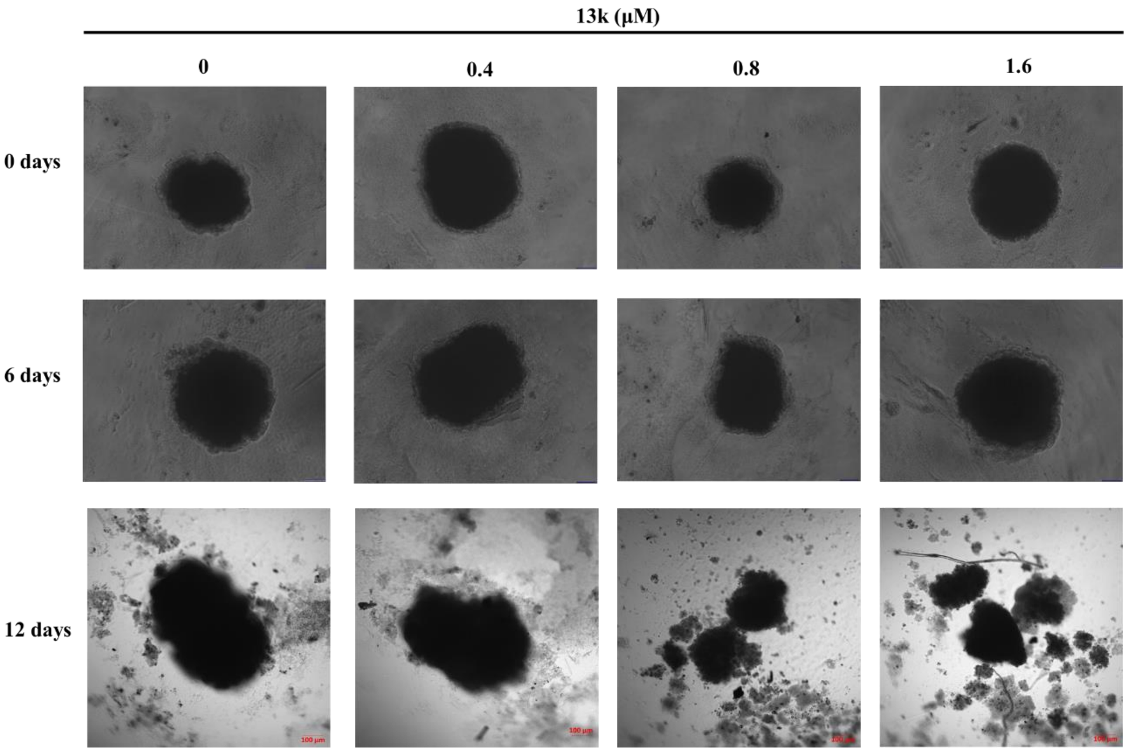

Figure 1. Effect of **13k** on HCC827 spheroid formation. Photographs were taken on days 0 and 6 with an inverted microscope (Olympus CKX53, JPN). After 12 days, pictures were taken with a ZEISS LSM 900 Airyscan 2 confocal laser scanning microscopy (ZEISS, GER). Scale bar: 100 μm.

2. Synthesis of compounds 10a-10u

2.1. General experimental protocol for the preparation of intermediates **5a-5q**.

2.1.1. Steps for the preparation of 6-iodo-N-(4-methoxybenzyl)quinazolin-4-amine (**5a**).

A mixture of 4-chloro-6-iodoquinazoline (2 mmol) and 4-methoxybenzylamine (2.4 mmol) was added to isopropanol and refluxed at 60 °C for 2 h under argon protection. After completion of the reaction (monitored by TLC), the solvent of the reaction mixture was removed under reduced pressure and extracted 2-3 times with ethyl acetate and saturated Na_2CO_3 solution. The organic phase was dried over anhydrous Na_2SO_4 and rotary dried under vacuum to obtain 6-iodo-N-(4-methoxybenzyl)quinazolin-4-amine **5a** as a white solid (77.0 % yield). ESI-MS: m/z 392.1$[M + H]^+$.

2.1.2. Steps for the preparation of N-(cyclopropylmethyl)-6-iodoquinazolin-4-amine (**5b**).

A mixture of 4-chloro-6-iodoquinazoline (2 mmol) and aminomethylcyclopropane (2.4 mmol) was added to isopropanol and refluxed at 60 °C for 2 h under argon protection. After completion of the reaction (monitored by TLC), the solvent of the reaction mixture was removed under reduced pressure and extracted 2-3 times with ethyl acetate and saturated Na_2CO_3 solution. The organic phase was dried over anhydrous Na_2SO_4 and rotary dried under vacuum to obtain N-(cyclopropylmethyl)-6-iodoquinazolin-4-amine **5b** as off-white solid (91.2 % yield). ESI-MS: m/z 326.0 $[M+H]^+$.

2.1.3. Steps for the preparation of N-(4-fluorobenzyl)-6-iodoquinazolin-4-amine (**5c**).

A mixture of 4-chloro-6-iodoquinazoline (2 mmol) and 4-fluorobenzylamine (2.4 mmol) was added to isopropanol and refluxed at 60 °C for 2 h under argon protection. After completion of the reaction (monitored by TLC), the solvent of the reaction mixture was removed under reduced pressure and extracted 2-3 times with ethyl acetate and saturated Na_2CO_3 solution. The organic phase was dried over anhydrous Na_2SO_4 and rotary dried under vacuum to obtain N-(4-fluorobenzyl)-6-iodoquinazolin-4-amine **5c** as off-white solid (77.8 % yield). ESI-MS: m/z 380.0 $[M+H]^+$.

2.1.4. Steps for the preparation of 6-iodo-N-(4-(trifluoromethyl)benzyl)quinazolin-4-amine (**5d**).

A mixture of 4-chloro-6-iodoquinazoline (2 mmol) and 4-(trifluoromethyl)benzylamine (2.4 mmol) was added to isopropanol and refluxed at 60 °C for 2 h under argon protection. After completion of the reaction (monitored by TLC), the solvent of the reaction mixture was removed under reduced pressure and extracted 2-3 times with ethyl acetate and saturated Na_2CO_3 solution. The organic phase was dried over anhydrous Na_2SO_4 and rotary dried under vacuum to obtain 6-iodo-N-(4-(trifluoromethyl)benzyl)quinazolin-4-amine **5d** as off-white solid (86.1 % yield). ESI-MS: m/z 430.2 $[M+H]^+$.

2.1.5. Steps for the preparation of 6-iodo-N-(3-methylbenzyl)quinazolin-4-amine(**5e**).

A mixture of 4-chloro-6-iodoquinazoline (2 mmol) and 3-methylbenzylamine (2.4 mmol) was added to isopropanol and refluxed at 60 °C for 2 h under argon protection. After completion of the reaction (monitored by TLC), the solvent of the reaction mixture was removed under reduced pressure and extracted 2-3 times with ethyl acetate and saturated Na_2CO_3 solution. The organic phase was dried over anhydrous Na_2SO_4 and rotary dried under vacuum to obtain 6-iodo-N-(3-methylbenzyl)quinazolin-4-amine **5e** as pale yellow solid (94.5 % yield). ESI-MS: m/z 376.2 $[M+H]^+$.

2.1.6. Steps for the preparation of N^1,N^1-diethyl-N^2-(6-iodoquinazolin-4-yl)ethane-1,2-diamine (**5f**).

A mixture of 4-chloro-6-iodoquinazoline (2 mmol) and N,N-Diethylethylenediamine (2.4 mmol) was added to isopropanol and refluxed at 60 °C for 2 h under argon protection. After completion of the reaction (monitored by TLC), the solvent of the reaction mixture was removed under reduced pressure and extracted 2-3 times with ethyl acetate and saturated Na_2CO_3 solution. The organic phase was dried over anhydrous Na_2SO_4 and rotary dried under vacuum to obtain N^1,N^1-diethyl-N^2-(6-iodoquinazolin-4-yl)ethane-1,2-diamine **5f** as pale yellow oily substance (91.0 % yield). ESI-MS: m/z 371.0 $[M+H]^+$.

2.1.7. Steps for the preparation of 6-iodo-N-(2-methylbenzyl)quinazolin-4-amine (**5g**).

A mixture of 4-chloro-6-iodoquinazoline (2 mmol) and 2-methylbenzylamine (2.4 mmol) was added to isopropanol and refluxed at 60 °C for 2 h under argon protection. After completion of the reaction (monitored by TLC), the solvent of the reaction mixture was removed under reduced pressure and extracted 2-3 times with ethyl acetate and saturated Na_2CO_3 solution. The organic phase was dried over anhydrous Na_2SO_4 and rotary dried under vacuum to obtain 6-iodo-N-(2-methylbenzyl)quinazolin-4-amine **5g** as pale yellow solid (95.3 % yield). ESI-MS: m/z 376.0 $[M+H]^+$.

2.1.8. Steps for the preparation of 6-iodo-N-(pyridin-2-ylmethyl)quinazolin-4-amine (**5h**).

A mixture of 4-chloro-6-iodoquinazoline (2 mmol) and 2-Picolylamine (2.4 mmol)

was added to isopropanol and refluxed at 60 °C for 2 h under argon protection. After completion of the reaction (monitored by TLC), the solvent of the reaction mixture was removed under reduced pressure and extracted 2-3 times with ethyl acetate and saturated Na_2CO_3 solution. The organic phase was dried over anhydrous Na_2SO_4 and rotary dried under vacuum to obtain 6-iodo-N-(pyridin-2-ylmethyl)quinazolin-4-amine **5h** as pale yellow solid (92.1 % yield). ESI-MS: m/z 385.2 $[M+Na]^+$.

2.1.9. Steps for the preparation of N-(2-fluorobenzyl)-6-iodoquinazolin-4-amine (**5i**).

A mixture of 4-chloro-6-iodoquinazoline (2 mmol) and 2-fluorobenzylamine (2.4 mmol) was added to isopropanol and refluxed at 60 °C for 2 h under argon protection. After completion of the reaction (monitored by TLC), the solvent of the reaction mixture was removed under reduced pressure and extracted 2-3 times with ethyl acetate and saturated Na_2CO_3 solution. The organic phase was dried over anhydrous Na_2SO_4 and rotary dried under vacuum to obtain N-(2-fluorobenzyl)-6-iodoquinazolin-4-amine **5i** as off-white solid (61.9 % yield). ESI-MS: m/z 380.0 $[M+H]^+$.

2.1.10. Steps for the preparation of N-(3-fluorophenyl)-6-iodoquinazolin-4-amine (**5j**).

A mixture of 4-chloro-6-iodoquinazoline (2 mmol) and 3-fluoroaniline (2.4 mmol) was added to isopropanol and refluxed at 60 °C for 2 h under argon protection. After completion of the reaction (monitored by TLC), the solvent of the reaction mixture was removed under reduced pressure and extracted 2-3 times with ethyl acetate and saturated Na_2CO_3 solution. The organic phase was dried over anhydrous Na_2SO_4 and rotary dried under vacuum to obtain N-(3-fluorophenyl)-6-iodoquinazolin-4-amine **5j** as pale yellow solid (85.3 % yield). ESI-MS: m/z 336.1 $[M+H]^+$.

2.1.11. Steps for the preparation of N-(3,5-dimethoxyphenyl)-6-iodoquinazolin-4-amine (**5k**).

A mixture of 4-chloro-6-iodoquinazoline (2 mmol) and 3,5-dimethoxyaniline (2.4 mmol) was added to isopropanol and refluxed at 60 °C for 2 h under argon protection. After completion of the reaction (monitored by TLC), the solvent of the reaction mixture was removed under reduced pressure and extracted 2-3 times with ethyl acetate and saturated Na_2CO_3 solution. The organic phase was dried over anhydrous Na_2SO_4

and rotary dried under vacuum to obtain N-(3,5-dimethoxyphenyl)-6-iodoquinazolin-4-amine **5k** as pale yellow solid (90.7 % yield). ESI-MS: m/z 408.2 $[M+H]^+$.

2.1.12. Steps for the preparation of 6-iodo-N-(pyridin-3-ylmethyl)quinazolin-4-amine (**5l**).

A mixture of 4-chloro-6-iodoquinazoline (2 mmol) and 3-(aminomethyl)pyridine (2.4 mmol) was added to isopropanol and refluxed at 60 °C for 2 h under argon protection. After completion of the reaction (monitored by TLC), the solvent of the reaction mixture was removed under reduced pressure and extracted 2-3 times with ethyl acetate and saturated Na_2CO_3 solution. The organic phase was dried over anhydrous Na_2SO_4 and rotary dried under vacuum to obtain 6-iodo-N-(pyridin-3-ylmethyl)quinazolin-4-amine **5l** as pink solid (94.2 % yield). ESI-MS: m/z 385.2 $[M+H]^+$.

2.1.13. Steps for the preparation of N-(2,3-difluorophenyl)-6-iodoquinazolin-4-amine (**5m**).

A mixture of 4-chloro-6-iodoquinazoline (2 mmol) and 2,3-difluoroaniline (2.4 mmol) was added to isopropanol and refluxed at 60 °C for 2 h under argon protection. After completion of the reaction (monitored by TLC), the solvent of the reaction mixture was removed under reduced pressure and extracted 2-3 times with ethyl acetate and saturated Na_2CO_3 solution. The organic phase was dried over anhydrous Na_2SO_4 and rotary dried under vacuum to obtain N-(2,3-difluorophenyl)-6-iodoquinazolin-4-amine **5m** as off-white solid (93.6 % yield). ESI-MS: m/z 384.1 $[M+H]^+$.

2.1.14. Steps for the preparation of 6-iodo-N-methyl-N-(p-tolyl)quinazolin-4-amine (**5n**).

A mixture of 4-chloro-6-iodoquinazoline (2 mmol) and N-methyl-p-toluidine (2.4 mmol) was added to isopropanol and refluxed at 60 °C for 2 h under argon protection. After completion of the reaction (monitored by TLC), the solvent of the reaction mixture was removed under reduced pressure and extracted 2-3 times with ethyl acetate and saturated Na_2CO_3 solution. The organic phase was dried over anhydrous Na_2SO_4 and rotary dried under vacuum to obtain 6-iodo-N-methyl-N-(p-tolyl)quinazolin-4-

amine **5n** as pale yellow solid (94.0 % yield). ESI-MS: m/z 376.0 $[M+H]^+$.

2.1.15. Steps for the preparation of N-ethyl-6-iodo-N-phenylquinazolin-4-amine (**5o**).

A mixture of 4-chloro-6-iodoquinazoline (2 mmol) and N-ethylaniline (2.4 mmol) was added to isopropanol and refluxed at 60 °C for 2 h under argon protection. After completion of the reaction (monitored by TLC), the solvent of the reaction mixture was removed under reduced pressure and extracted 2-3 times with ethyl acetate and saturated Na_2CO_3 solution. The organic phase was dried over anhydrous Na_2SO_4 and rotary dried under vacuum to obtain N-ethyl-6-iodo-N-phenylquinazolin-4-amine **5o** as pale yellow solid (95.7 % yield). ESI-MS: m/z 376.0 $[M+H]^+$.

2.1.16. Steps for the preparation of 6-iodo-N-(1H-pyrazol-3-yl)quinazolin-4-amine (**5p**).

A mixture of 4-chloro-6-iodoquinazoline (2 mmol) and 3-aminopyrazole (2.4 mmol) was added to isopropanol and refluxed at 60 °C for 2 h under argon protection. After completion of the reaction (monitored by TLC), the solvent of the reaction mixture was removed under reduced pressure and extracted 2-3 times with ethyl acetate and saturated Na_2CO_3 solution. The organic phase was dried over anhydrous Na_2SO_4 and rotary dried under vacuum to obtain 6-iodo-N-(1H-pyrazol-3-yl)quinazolin-4-amine **5p** as white solid (91.5 % yield). ESI-MS: m/z 338.1 $[M+H]^+$.

2.1.17. Steps for the preparation of 6-iodo-N-((tetrahydro-2H-pyran-4-yl)methyl)quinazolin-4-amine (**5q**).

A mixture of 4-chloro-6-iodoquinazoline (2 mmol) and 4-(aminomethyl)tetrahydro-2H-pyran (2.4 mmol) was added to isopropanol and refluxed at 60 °C for 2 h under argon protection. After completion of the reaction (monitored by TLC), the solvent of the reaction mixture was removed under reduced pressure and extracted 2-3 times with ethyl acetate and saturated Na_2CO_3 solution. The organic phase was dried over anhydrous Na_2SO_4 and rotary dried under vacuum to obtain 6-iodo-N-((tetrahydro-2H-pyran-4-yl)methyl)quinazolin-4-amine **5q** as white solid (90.1 % yield). ESI-MS: m/z 392.0 $[M+H]^+$.

2.2. General experimental protocol for the preparation of intermediates **7a-7o**

2.2.1. Steps for the preparation of 6-iodo-N-(4-methoxybenzyl)quinazolin-4-amine

(**7a**).

6-iodo-N-(4-methoxybenzyl)quinazolin-4-amine **5a** (1.5 mmol), 5-(4,4,5,5-tetramethyl-1,3,2-dioxaborolan-2-yl)pyridin-2-amine (1.5 mmol) and K_2CO_3 (4.6 mmol) were added to 15 mL of solvent [$V_{(1,4\text{-dioxane})}$:$V_{(water)}$ = 4:1]. The mixture was heated to 100 °C under a protective atmosphere of argon followed by the addition of $Pd(dppf)Cl_2$. The mixture continues to be stirred under these conditions for a further 4-6 hours. After completion of the reaction (monitored by TLC), the 1, 4-dioxane and water were removed under reduced pressure and the residue was puri-fied through a column chromatography on silica with dichloromethane/methanol to afford white solid 6-(6-aminopyridin-3-yl)-N-(4-methoxybenzyl)quinazolin-4-amine **7a** (66.6 % yield), ESI-MS: m/z 358.1 $[M + H]^+$.

2.2.2. Steps for the preparation of 6-(6-aminopyridin-3-yl)-N-(cyclopropylmethyl)quinazolin-4-amine (**7b**).

N-(cyclopropylmethyl)-6-iodoquinazolin-4-amine **5b** (1.5 mmol), 5-(4,4,5,5-tetramethyl-1,3,2-dioxaborolan-2-yl)pyridin-2-amine (1.5 mmol) and K_2CO_3 (4.6 mmol) were added to 15 mL of solvent [$V_{(1,4\text{-dioxane})}$:$V_{(water)}$ = 4:1]. The mixture was heated to 100 °C under a protective atmosphere of argon followed by the addition of $Pd(dppf)Cl_2$. The mixture continues to be stirred under these conditions for a further 4-6 hours. After completion of the reaction (monitored by TLC), the 1, 4-dioxane and water were removed under reduced pressure and the residue was puri-fied through a column chromatography on silica with dichloromethane/methanol to afford off-white solid 6-(6-aminopyridin-3-yl)-N-(cyclopropylmethyl)quinazolin-4-amine **7b** (71.2 % yield), ESI-MS: m/z 291.1 $[M + H]^+$.

2.2.3. Steps for the preparation of 6-(6-aminopyridin-3-yl)-N-(4-fluorobenzyl)quinazolin-4-amine (**7c**).

N-(4-fluorobenzyl)-6-iodoquinazolin-4-amine **5c** (1.5 mmol), 5-(4,4,5,5-tetramethyl-1,3,2-dioxaborolan-2-yl)pyridin-2-amine (1.5 mmol) and K_2CO_3 (4.6 mmol) were added to 15 mL of solvent [$V_{(1,4\text{-dioxane})}$:$V_{(water)}$ = 4:1]. The mixture was heated to 100 °C under a protective atmosphere of argon followed by the addition of

$Pd(dppf)Cl_2$. The mixture continues to be stirred under these conditions for a further 4-6 hours. After completion of the reaction (monitored by TLC), the 1, 4-dioxane and water were removed under reduced pressure and the residue was puri-fied through a column chromatography on silica with dichloromethane/methanol to afford off-white solid 6-(6-aminopyridin-3-yl)-N-(4-fluorobenzyl)quinazolin-4-amine **7c** (92.2 % yield), ESI-MS: m/z 246.1 $[M + H]^+$.

2.2.4. Steps for the preparation of 6-(6-aminopyridin-3-yl)-N-(4-(trifluoromethyl)benzyl)quinazolin-4-amine (**7d**).

6-iodo-N-(4-(trifluoromethyl)benzyl)quinazolin-4-amine **5d** (1.5 mmol), 5-(4,4,5,5-tetramethyl-1,3,2-dioxaborolan-2-yl)pyridin-2-amine (1.5 mmol) and K_2CO_3 (4.6 mmol) were added to 15 mL of solvent [$V_{(1,4\text{-dioxane})}$:$V_{(water)}$ = 4:1]. The mixture was heated to 100 °C under a protective atmosphere of argon followed by the addition of $Pd(dppf)Cl_2$. The mixture continues to be stirred under these conditions for a further 4-6 hours. After completion of the reaction (monitored by TLC), the 1, 4-dioxane and water were removed under reduced pressure and the residue was puri-fied through a column chromatography on silica with dichloromethane/methanol to afford off-white solid 6-(6-aminopyridin-3-yl)-N-(4-(trifluoromethyl)benzyl)quinazolin-4-amine **7d** (85.7 % yield), ESI-MS: m/z 396.1 $[M + H]^+$.

2.2.5. Steps for the preparation of 6-(6-aminopyridin-3-yl)-N-(3-methylbenzyl)quinazolin-4-amine (**7e**).

6-iodo-N-(3-methylbenzyl)quinazolin-4-amine **5e** (1.5 mmol), 5-(4,4,5,5-tetramethyl-1,3,2-dioxaborolan-2-yl)pyridin-2-amine (1.5 mmol) and K_2CO_3 (4.6 mmol) were added to 15 mL of solvent [$V_{(1,4\text{-dioxane})}$:$V_{(water)}$ = 4:1]. The mixture was heated to 100 °C under a protective atmosphere of argon followed by the addition of $Pd(dppf)Cl_2$. The mixture continues to be stirred under these conditions for a further 4-6 hours. After completion of the reaction (monitored by TLC), the 1, 4-dioxane and water were removed under reduced pressure and the residue was puri-fied through a column chromatography on silica with dichloromethane/methanol to afford off-white solid 6-(6-aminopyridin-3-yl)-N-(3-methylbenzyl)quinazolin-4-amine **7e** (73.4 %

yield), ESI-MS: m/z 342.1 $[M + H]^+$.

2.2.6. Steps for the preparation of N^1-(6-(6-aminopyridin-3-yl)quinazolin-4-yl)-N^2,N^2-diethylethane-1,2-diamine (**7f**).

N^1,N^1-diethyl-N^2-(6-iodoquinazolin-4-yl)ethane-1,2-diamine **5f** (1.5 mmol), 5-(4,4,5,5-tetramethyl-1,3,2-dioxaborolan-2-yl)pyridin-2-amine (1.5 mmol) and K_2CO_3 (4.6 mmol) were added to 15 mL of solvent [$V_{(1,4\text{-dioxane})}$:$V_{(water)}$ = 4:1]. The mixture was heated to 100 °C under a protective atmosphere of argon followed by the addition of $Pd(dppf)Cl_2$. The mixture continues to be stirred under these conditions for a further 4-6 hours. After completion of the reaction (monitored by TLC), the 1, 4-dioxane and water were removed under reduced pressure and the residue was puri-fied through a column chromatography on silica with dichloromethane/methanol to afford brown solid N^1-(6-(6-aminopyridin-3-yl)quinazolin-4-yl)-N^2,N^2-diethylethane-1,2-diamine **7f** (82.9 % yield), ESI-MS: m/z 359.1 $[M + H]^+$.

2.2.7. Steps for the preparation of 6-(6-aminopyridin-3-yl)-N-(2-methylbenzyl)quinazolin-4-amine (**7g**).

6-iodo-N-(2-methylbenzyl)quinazolin-4-amine **5g** (1.5 mmol), 5-(4,4,5,5-tetramethyl-1,3,2-dioxaborolan-2-yl)pyridin-2-amine (1.5 mmol) and K_2CO_3 (4.6 mmol) were added to 15 mL of solvent [$V_{(1,4\text{-dioxane})}$:$V_{(water)}$ = 4:1]. The mixture was heated to 100 °C under a protective atmosphere of argon followed by the addition of $Pd(dppf)Cl_2$. The mixture continues to be stirred under these conditions for a further 4-6 hours. After completion of the reaction (monitored by TLC), the 1, 4-dioxane and water were removed under reduced pressure and the residue was puri-fied through a column chromatography on silica with dichloromethane/methanol to afford off-white solid 6-(6-aminopyridin-3-yl)-N-(2-methylbenzyl)quinazolin-4-amine **7g** (76.3 % yield), ESI-MS: m/z 342.1 $[M + H]^+$.

2.2.8. Steps for the preparation of 6-(6-aminopyridin-3-yl)-N-(pyridin-2-ylmethyl)quinazolin-4-amine (**7h**).

6-iodo-N-(pyridin-2-ylmethyl)quinazolin-4-amine **5h** (1.5 mmol), 5-(4,4,5,5-tetramethyl-1,3,2-dioxaborolan-2-yl)pyridin-2-amine (1.5 mmol) and K_2CO_3 (4.6

mmol) were added to 15 mL of solvent [V $_{(1,4\text{-dioxane})}$:V $_{(water)}$ = 4:1]. The mixture was heated to 100 °C under a protective atmosphere of argon followed by the addition of Pd(dppf)Cl_2. The mixture continues to be stirred under these conditions for a further 4-6 hours. After completion of the reaction (monitored by TLC), the 1, 4-dioxane and water were removed under reduced pressure and the residue was puri-fied through a column chromatography on silica with dichloromethane/methanol to afford yellow solid 6-(6-aminopyridin-3-yl)-N-(pyridin-2-ylmethyl)quinazolin-4-amine **7h** (70.6 % yield), ESI-MS: m/z 328.1 $[M + H]^+$.

2.2.9. Steps for the preparation of 6-(6-aminopyridin-3-yl)-N-(2-fluorobenzyl)quinazolin-4-amine (**7i**).

N-(2-fluorobenzyl)-6-iodoquinazolin-4-amine **5i** (1.5 mmol), 5-(4,4,5,5-tetramethyl-1,3,2-dioxaborolan-2-yl)pyridin-2-amine (1.5 mmol) and K_2CO_3 (4.6 mmol) were added to 15 mL of solvent [V $_{(1,4\text{-dioxane})}$:V $_{(water)}$ = 4:1]. The mixture was heated to 100 °C under a protective atmosphere of argon followed by the addition of Pd(dppf)Cl_2. The mixture continues to be stirred under these conditions for a further 4-6 hours. After completion of the reaction (monitored by TLC), the 1, 4-dioxane and water were removed under reduced pressure and the residue was puri-fied through a column chromatography on silica with dichloromethane/methanol to afford off-white solid 6-(6-aminopyridin-3-yl)-N-(2-fluorobenzyl)quinazolin-4-amine **7i** (86.3 % yield), ESI-MS: m/z 346.1 $[M + H]^+$.

2.2.10. Steps for the preparation of 6-(6-aminopyridin-3-yl)-N-(3-fluorophenyl)quinazolin-4-amine (**7j**).

N-(3-fluorophenyl)-6-iodoquinazolin-4-amine **5j** (1.5 mmol), 5-(4,4,5,5-tetramethyl-1,3,2-dioxaborolan-2-yl)pyridin-2-amine (1.5 mmol) and K_2CO_3 (4.6 mmol) were added to 15 mL of solvent [V $_{(1,4\text{-dioxane})}$:V $_{(water)}$ = 4:1]. The mixture was heated to 100 °C under a protective atmosphere of argon followed by the addition of Pd(dppf)Cl_2. The mixture continues to be stirred under these conditions for a further 4-6 hours. After completion of the reaction (monitored by TLC), the 1, 4-dioxane and water were removed under reduced pressure and the residue was puri-fied through a

column chromatography on silica with dichloromethane/methanol to afford pale yellow solid 6-(6-aminopyridin-3-yl)-N-(3-fluorophenyl)quinazolin-4-amine **7j** (77.6 % yield), ESI-MS: m/z 332.1 $[M + H]^+$.

2.2.11. Steps for the preparation of 6-(6-aminopyridin-3-yl)-N-(3,5-dimethoxyphenyl)quinazolin-4-amine (**7k**).

N-(3,5-dimethoxyphenyl)-6-iodoquinazolin-4-amine **5k** (1.5 mmol), 5-(4,4,5,5-tetramethyl-1,3,2-dioxaborolan-2-yl)pyridin-2-amine (1.5 mmol) and K_2CO_3 (4.6 mmol) were added to 15 mL of solvent $[V_{(1,4\text{-dioxane})}:V_{(water)} = 4:1]$. The mixture was heated to 100 °C under a protective atmosphere of argon followed by the addition of $Pd(dppf)Cl_2$. The mixture continues to be stirred under these conditions for a further 4-6 hours. After completion of the reaction (monitored by TLC), the 1, 4-dioxane and water were removed under reduced pressure and the residue was puri-fied through a column chromatography on silica with dichloromethane/methanol to afford yellow solid 6-(6-aminopyridin-3-yl)-N-(3,5-dimethoxyphenyl)quinazolin-4-amine **7k** (81.3 % yield), ESI-MS: m/z 396.1 $[M + Na]^+$.

2.2.12. Steps for the preparation of 6-(6-aminopyridin-3-yl)-N-(pyridin-3-ylmethyl)quinazolin-4-amine (**7l**).

6-iodo-N-(pyridin-3-ylmethyl)quinazolin-4-amine **5l** (1.5 mmol), 5-(4,4,5,5-tetramethyl-1,3,2-dioxaborolan-2-yl)pyridin-2-amine (1.5 mmol) and K_2CO_3 (4.6 mmol) were added to 15 mL of solvent $[V_{(1,4\text{-dioxane})}:V_{(water)} = 4:1]$. The mixture was heated to 100 °C under a protective atmosphere of argon followed by the addition of $Pd(dppf)Cl_2$. The mixture continues to be stirred under these conditions for a further 4-6 hours. After completion of the reaction (monitored by TLC), the 1, 4-dioxane and water were removed under reduced pressure and the residue was puri-fied through a column chromatography on silica with dichloromethane/methanol to afford off-white solid 6-(6-aminopyridin-3-yl)-N-(pyridin-3-ylmethyl)quinazolin-4-amine **7l** (67.6 % yield), ESI-MS: m/z 329.1 $[M + H]^+$.

2.2.13. Steps for the preparation of 6-(6-aminopyridin-3-yl)-N-(2,3-difluorophenyl)quinazolin-4-amine (**7m**).

N-(2,3-difluorophenyl)-6-iodoquinazolin-4-amine **5m** (1.5 mmol), 5-(4,4,5,5-tetramethyl-1,3,2-dioxaborolan-2-yl)pyridin-2-amine (1.5 mmol) and K_2CO_3 (4.6 mmol) were added to 15 mL of solvent [$V_{(1,4\text{-dioxane})}$:$V_{(water)}$ = 4:1]. The mixture was heated to 100 °C under a protective atmosphere of argon followed by the addition of $Pd(dppf)Cl_2$. The mixture continues to be stirred under these conditions for a further 4-6 hours. After completion of the reaction (monitored by TLC), the 1, 4-dioxane and water were removed under reduced pressure and the residue was puri-fied through a column chromatography on silica with dichloromethane/methanol to afford white solid 6-(6-aminopyridin-3-yl)-N-(2,3-difluorophenyl)quinazolin-4-amine **7m** (88.7 % yield), ESI-MS: m/z 352.1 $[M + Na]^+$.

2.2.14. Steps for the preparation of 6-(6-aminopyridin-3-yl)-N-methyl-N-(p-tolyl)quinazolin-4-amine (**7n**).

6-iodo-N-methyl-N-(p-tolyl)quinazolin-4-amine **5n** (1.5 mmol), 5-(4,4,5,5-tetramethyl-1,3,2-dioxaborolan-2-yl)pyridin-2-amine (1.5 mmol) and K_2CO_3 (4.6 mmol) were added to 15 mL of solvent [$V_{(1,4\text{-dioxane})}$:$V_{(water)}$ = 4:1]. The mixture was heated to 100 °C under a protective atmosphere of argon followed by the addition of $Pd(dppf)Cl_2$. The mixture continues to be stirred under these conditions for a further 4-6 hours. After completion of the reaction (monitored by TLC), the 1, 4-dioxane and water were removed under reduced pressure and the residue was puri-fied through a column chromatography on silica with dichloromethane/methanol to afford pale yellow solid 6-(6-aminopyridin-3-yl)-N-methyl-N-(p-tolyl)quinazolin-4-amine **7n** (79.8 % yield), ESI-MS: m/z 342.1 $[M + H]^+$.

2.2.15. Steps for the preparation of 6-(6-aminopyridin-3-yl)-N-ethyl-N-phenylquinazolin-4-amine (**7o**).

N-ethyl-6-iodo-N-phenylquinazolin-4-amine **5o** (1.5 mmol), 5-(4,4,5,5-tetramethyl-1,3,2-dioxaborolan-2-yl)pyridin-2-amine (1.5 mmol) and K_2CO_3 (4.6 mmol) were added to 15 mL of solvent [$V_{(1,4\text{-dioxane})}$:$V_{(water)}$ = 4:1]. The mixture was heated to 100 °C under a protective atmosphere of argon followed by the addition of $Pd(dppf)Cl_2$. The mixture continues to be stirred under these conditions for a further 4-

6 hours. After completion of the reaction (monitored by TLC), the 1, 4-dioxane and water were removed under reduced pressure and the residue was puri-fied through a column chromatography on silica with dichloromethane/methanol to afford yellow solid 6-(6-aminopyridin-3-yl)-N-ethyl-N-phenylquinazolin-4-amine **7o** (75.7 % yield), ESI-MS: m/z 342.1 $[M + H]^+$.

2.3. General experimental protocol for the preparation of compounds **10a-10u.**

2.3.1. Procedure for the preparation of ethyl 6-(4-((4-methoxybenzyl)amino)quinazolin-6-yl)imidazo[1,2-a]pyridine-2-carboxylate (**10a**).

A mixture of 6-iodo-N-(4-methoxybenzyl)quinazolin-4-amine **7a** (0.5 mmol), ethyl bromopyruvate (1.5 mmol) and $NaHCO_3$ (1.5 mmol) was added to EtOH (5 mL) and the mixture was warmed to 80 °C and refluxed by condensation under argon for 4 hours. After completion of the reaction (monitored by TLC), the solvents were removed under reduced pressure and the residue was purified through a column chromatography on silica with dichloromethane/methanol to obtain ethyl 6-(4-((4-methoxybenzyl)amino)quinazolin-6-yl)imidazo[1,2-a]pyridine-2-carboxylate **10a** as white solid (62.0 % yield).

2.3.2. Procedure for the preparation of ethyl 6-(4-((cyclopropylmethyl)amino)quinazolin-6-yl)imidazo[1,2-a]pyridine-2-carboxylate (**10b**).

6-(6-aminopyridin-3-yl)-N-(cyclopropylmethyl)quinazolin-4-amine **7b** (0.5 mmol), ethyl bromopyruvate (1.5 mmol) and $NaHCO_3$ (1.5 mmol) was added to EtOH (5 mL) and the mixture was warmed to 80 °C and refluxed by condensation under argon for 4 hours. After completion of the reaction (monitored by TLC), the solvents were removed under reduced pressure and the residue was purified through a column chromatography on silica with dichloromethane/methanol to obtain ethyl 6-(4-((cyclopropylmethyl)amino)quinazolin-6-yl)imidazo[1,2-a]pyridine-2-carboxylate **10b** as white solid (49.8 % yield).

2.3.3. Procedure for the preparation of ethyl 6-(4-((4-fluorobenzyl)amino)quinazolin-6-yl)imidazo[1,2-a]pyridine-2-carboxylate (**10c**).

6-(6-aminopyridin-3-yl)-N-(4-fluorobenzyl)quinazolin-4-amine **7c** (0.5 mmol),

ethyl bromopyruvate (1.5 mmol) and $NaHCO_3$ (1.5 mmol) was added to EtOH (5 mL) and the mixture was warmed to 80 °C and refluxed by condensation under argon for 4 hours. After completion of the reaction (monitored by TLC), the solvents were removed under reduced pressure and the residue was purified through a column chromatography on silica with dichloromethane/methanol to obtain ethyl 6-(4-((4-fluorobenzyl)amino)quinazolin-6-yl)imidazo[1,2-a]pyridine-2-carboxylate **10c** as white solid (55.1 % yield).

2.3.4. Procedure for the preparation of ethyl 6-(4-((4-(trifluoromethyl)benzyl)amino)quinazolin-6-yl)imidazo[1,2-a]pyridine-2-carboxylate (**10d**).

6-(6-aminopyridin-3-yl)-N-(4-(trifluoromethyl)benzyl)quinazolin-4-amine **7d** (0.5 mmol), ethyl bromopyruvate (1.5 mmol) and $NaHCO_3$ (1.5 mmol) was added to EtOH (5 mL) and the mixture was warmed to 80 °C and refluxed by condensation under argon for 4 hours. After completion of the reaction (monitored by TLC), the solvents were removed under reduced pressure and the residue was purified through a column chromatography on silica with dichloromethane/methanol to obtain ethyl 6-(4-((4-(trifluoromethyl)benzyl)amino)quinazolin-6-yl)imidazo[1,2-a]pyridine-2-carboxylate **10d** as white solid (56.8 % yield).

2.3.5. Procedure for the preparation of ethyl 6-(4-((3-methylbenzyl)amino)quinazolin-6-yl)imidazo[1,2-a]pyridine-2-carboxylate (**10e**).

6-(6-aminopyridin-3-yl)-N-(3-methylbenzyl)quinazolin-4-amine **7e** (0.5 mmol), ethyl bromopyruvate (1.5 mmol) and $NaHCO_3$ (1.5 mmol) was added to EtOH (5 mL) and the mixture was warmed to 80 °C and refluxed by condensation under argon for 4 hours. After completion of the reaction (monitored by TLC), the solvents were removed under reduced pressure and the residue was purified through a column chromatography on silica with dichloromethane/methanol to obtain ethyl 6-(4-((3-methylbenzyl)amino)quinazolin-6-yl)imidazo[1,2-a]pyridine-2-carboxylate **10e** as off-white solid (53.0 % yield).

2.3.6. Procedure for the preparation of ethyl 6-(4-((2-

(diethylamino)ethyl)amino)quinazolin-6-yl)imidazo[1,2-a]pyridine-2-carboxylate (**10f**).

N^1-(6-(6-aminopyridin-3-yl)quinazolin-4-yl)-N^2,N^2-diethylethane-1,2-diamine **7f** (0.5 mmol), ethyl bromopyruvate (1.5 mmol) and $NaHCO_3$ (1.5 mmol) was added to EtOH (5 mL) and the mixture was warmed to 80 °C and refluxed by condensation under argon for 4 hours. After completion of the reaction (monitored by TLC), the solvents were removed under reduced pressure and the residue was purified through a column chromatography on silica with dichloromethane/methanol to obtain ethyl 6-(4-((2-(diethylamino)ethyl)amino)quinazolin-6-yl)imidazo[1,2-a]pyridine-2-carboxylate **10f** as brown solid (42.1 % yield).

2.3.7. Procedure for the preparation of ethyl 6-(4-((2-methylbenzyl)amino)quinazolin-6-yl)imidazo[1,2-a]pyridine-2-carboxylate (**10g**).

6-(6-aminopyridin-3-yl)-N-(2-methylbenzyl)quinazolin-4-amine **7g** (0.5 mmol), ethyl bromopyruvate (1.5 mmol) and $NaHCO_3$ (1.5 mmol) was added to EtOH (5 mL) and the mixture was warmed to 80 °C and refluxed by condensation under argon for 4 hours. After completion of the reaction (monitored by TLC), the solvents were removed under reduced pressure and the residue was purified through a column chromatography on silica with dichloromethane/methanol to obtain ethyl 6-(4-((2-methylbenzyl)amino)quinazolin-6-yl)imidazo[1,2-a]pyridine-2-carboxylate **10g** as pink solid (45.8 % yield).

2.3.8. Procedure for the preparation of ethyl 6-(4-((pyridin-2-ylmethyl)amino)quinazolin-6-yl)imidazo[1,2-a]pyridine-2-carboxylate (**10h**).

6-(6-aminopyridin-3-yl)-N-(pyridin-2-ylmethyl)quinazolin-4-amine **7h** (0.5 mmol), ethyl bromopyruvate (1.5 mmol) and $NaHCO_3$ (1.5 mmol) was added to EtOH (5 mL) and the mixture was warmed to 80 °C and refluxed by condensation under argon for 4 hours. After completion of the reaction (monitored by TLC), the solvents were removed under reduced pressure and the residue was purified through a column chromatography on silica with dichloromethane/methanol to obtain ethyl 6-(4-((2-methylbenzyl)amino)quinazolin-6-yl)imidazo[1,2-a]pyridine-2-carboxylate **10h** as

yellow solid (57.3 % yield).

2.3.9. Procedure for the preparation of ethyl 6-(4-((2-fluorobenzyl)amino)quinazolin-6-yl)imidazo[1,2-a]pyridine-2-carboxylate (**10i**).

6-(6-aminopyridin-3-yl)-N-(2-fluorobenzyl)quinazolin-4-amine **7i** (0.5 mmol), ethyl bromopyruvate (1.5 mmol) and $NaHCO_3$ (1.5 mmol) was added to EtOH (5 mL) and the mixture was warmed to 80 °C and refluxed by condensation under argon for 4 hours. After completion of the reaction (monitored by TLC), the solvents were removed under reduced pressure and the residue was purified through a column chromatography on silica with dichloromethane/methanol to obtain ethyl 6-(4-((2-fluorobenzyl)amino)quinazolin-6-yl)imidazo[1,2-a]pyridine-2-carboxylate **10i** as off-white solid (59.2 % yield).

2.3.10. Procedure for the preparation of ethyl 6-(4-((3-fluorophenyl)amino)quinazolin-6-yl)imidazo[1,2-a]pyridine-2-carboxylate (**10j**).

6-(6-aminopyridin-3-yl)-N-(3-fluorophenyl)quinazolin-4-amine **7j** (0.5 mmol), ethyl bromopyruvate (1.5 mmol) and $NaHCO_3$ (1.5 mmol) was added to EtOH (5 mL) and the mixture was warmed to 80 °C and refluxed by condensation under argon for 4 hours. After completion of the reaction (monitored by TLC), the solvents were removed under reduced pressure and the residue was purified through a column chromatography on silica with dichloromethane/methanol to obtain ethyl 6-(4-((3-fluorophenyl)amino)quinazolin-6-yl)imidazo[1,2-a]pyridine-2-carboxylate **10j** as yellow solid (46.7 % yield).

2.3.11. Procedure for the preparation of ethyl 6-(4-((3,5-dimethoxyphenyl)amino)quinazolin-6-yl)imidazo[1,2-a]pyridine-2-carboxylate (**10k**).

6-(6-aminopyridin-3-yl)-N-(3,5-dimethoxyphenyl)quinazolin-4-amine **7k** (0.5 mmol), ethyl bromopyruvate (1.5 mmol) and $NaHCO_3$ (1.5 mmol) was added to EtOH (5 mL) and the mixture was warmed to 80 °C and refluxed by condensation under argon for 4 hours. After completion of the reaction (monitored by TLC), the solvents were removed under reduced pressure and the residue was purified through a column chromatography on silica with dichloromethane/methanol to obtain ethyl 6-(4-((3,5-

dimethoxyphenyl)amino)quinazolin-6-yl)imidazo[1,2-a]pyridine-2-carboxylate **10k** as yellow solid (50.2 % yield).

2.3.12. Procedure for the preparation of ethyl 6-(4-((2,3-difluorophenyl)amino)quinazolin-6-yl)imidazo[1,2-a]pyridine-2-carboxylate (**10l**).

6-(6-aminopyridin-3-yl)-N-(2,3-difluorophenyl)quinazolin-4-amine **7m** (0.5 mmol), ethyl bromopyruvate (1.5 mmol) and $NaHCO_3$ (1.5 mmol) was added to EtOH (5 mL) and the mixture was warmed to 80 °C and refluxed by condensation under argon for 4 hours. After completion of the reaction (monitored by TLC), the solvents were removed under reduced pressure and the residue was purified through a column chromatography on silica with dichloromethane/methanol to obtain ethyl 6-(4-((2,3-difluorophenyl)amino)quinazolin-6-yl)imidazo[1,2-a]pyridine-2-carboxylate **10l** as white solid (55.3 % yield).

2.3.13. Procedure for the preparation of ethyl 6-(4-(ethyl(phenyl)amino)quinazolin-6-yl)imidazo[1,2-a]pyridine-2-carboxylate (**10m**).

6-(6-aminopyridin-3-yl)-N-ethyl-N-phenylquinazolin-4-amine **7o** (0.5 mmol), ethyl bromopyruvate (1.5 mmol) and $NaHCO_3$ (1.5 mmol) was added to EtOH (5 mL) and the mixture was warmed to 80 °C and refluxed by condensation under argon for 4 hours. After completion of the reaction (monitored by TLC), the solvents were removed under reduced pressure and the residue was purified through a column chromatography on silica with dichloromethane/methanol to obtain ethyl 6-(4-(ethyl(phenyl)amino)quinazolin-6-yl)imidazo[1,2-a]pyridine-2-carboxylate **10m** as pink flocculent (63.1 % yield).

2.3.14. Procedure for the preparation of ethyl 6-(4-((pyridin-3-ylmethyl)amino)quinazolin-6-yl)imidazo[1,2-a]pyridine-2-carboxylate (**10n**).

6-(6-aminopyridin-3-yl)-N-(pyridin-3-ylmethyl)quinazolin-4-amine **7l** (0.5 mmol), ethyl bromopyruvate (1.5 mmol) and $NaHCO_3$ (1.5 mmol) was added to EtOH (5 mL) and the mixture was warmed to 80 °C and refluxed by condensation under argon for 4 hours. After completion of the reaction (monitored by TLC), the solvents were removed under reduced pressure and the residue was purified through a column

chromatography on silica with dichloromethane/methanol to obtain ethyl 6-(4-((pyridin-3-ylmethyl)amino)quinazolin-6-yl)imidazo[1,2-a]pyridine-2-carboxylate **10n** as white solid (63.1 % yield).

2.3.15. Procedure for the preparation of methyl 6-(4-((4-methoxybenzyl)amino)quinazolin-6-yl)imidazo[1,2-a]pyridine-2-carboxylate (**10o**).

A mixture of 6-iodo-N-(4-methoxybenzyl)quinazolin-4-amine **7a** (0.5 mmol), methyl bromopyruvate (1.5 mmol) and $NaHCO_3$ (1.5 mmol) was added to EtOH (5 mL) and the mixture was warmed to 80 °C and refluxed by condensation under argon for 4 hours. After completion of the reaction (monitored by TLC), the solvents were removed under reduced pressure and the residue was purified through a column chromatography on silica with dichloromethane/methanol to obtain methyl 6-(4-((4-methoxybenzyl)amino)quinazolin-6-yl)imidazo[1,2-a]pyridine-2-carboxylate **10o** as white solid (43.7 % yield).

2.3.16. Procedure for the preparation of methyl 6-(4-((2-methylbenzyl)amino)quinazolin-6-yl)imidazo[1,2-a]pyridine-2-carboxylate (**10p**).

6-(6-aminopyridin-3-yl)-N-(2-methylbenzyl)quinazolin-4-amine **7g** (0.5 mmol), methyl bromopyruvate (1.5 mmol) and $NaHCO_3$ (1.5 mmol) was added to EtOH (5 mL) and the mixture was warmed to 80 °C and refluxed by condensation under argon for 4 hours. After completion of the reaction (monitored by TLC), the solvents were removed under reduced pressure and the residue was purified through a column chromatography on silica with dichloromethane/methanol to obtain methyl 6-(4-((2-methylbenzyl)amino)quinazolin-6-yl)imidazo[1,2-a]pyridine-2-carboxylate **10p** as off-white solid (36.7 % yield).

2.3.17. Procedure for the preparation of methyl 6-(4-((pyridin-2-ylmethyl)amino)quinazolin-6-yl)imidazo[1,2-a]pyridine-2-carboxylate (**10q**).

6-(6-aminopyridin-3-yl)-N-(pyridin-2-ylmethyl)quinazolin-4-amine **7h** (0.5 mmol), methyl bromopyruvate (1.5 mmol) and $NaHCO_3$ (1.5 mmol) was added to EtOH (5 mL) and the mixture was warmed to 80 °C and refluxed by condensation under argon for 4 hours. After completion of the reaction (monitored by TLC), the solvents

were removed under reduced pressure and the residue was purified through a column chromatography on silica with dichloromethane/methanol to obtain methyl 6-(4-((pyridin-2-ylmethyl)amino)quinazolin-6-yl)imidazo[1,2-a]pyridine-2-carboxylate **10q** as yellow solid (53.8 % yield).

2.3.18. Procedure for the preparation of methyl 6-(4-((2-fluorobenzyl)amino)quinazolin-6-yl)imidazo[1,2-a]pyridine-2-carboxylate (**10r**).

6-(6-aminopyridin-3-yl)-N-(2-fluorobenzyl)quinazolin-4-amine **7i** (0.5 mmol), methyl bromopyruvate (1.5 mmol) and $NaHCO_3$ (1.5 mmol) was added to EtOH (5 mL) and the mixture was warmed to 80 °C and refluxed by condensation under argon for 4 hours. After completion of the reaction (monitored by TLC), the solvents were removed under reduced pressure and the residue was purified through a column chromatography on silica with dichloromethane/methanol to obtain methyl 6-(4-((2-fluorobenzyl)amino)quinazolin-6-yl)imidazo[1,2-a]pyridine-2-carboxylate **10r** as off-white solid (52.1 % yield).

2.3.19. Procedure for the preparation of methyl 6-(4-((3-fluorophenyl)amino)quinazolin-6-yl)imidazo[1,2-a]pyridine-2-carboxylate (**10s**).

6-(6-aminopyridin-3-yl)-N-(3-fluorophenyl)quinazolin-4-amine **7j** (0.5 mmol), methyl bromopyruvate (1.5 mmol) and $NaHCO_3$ (1.5 mmol) was added to EtOH (5 mL) and the mixture was warmed to 80 °C and refluxed by condensation under argon for 4 hours. After completion of the reaction (monitored by TLC), the solvents were removed under reduced pressure and the residue was purified through a column chromatography on silica with dichloromethane/methanol to obtain methyl 6-(4-((3-fluorophenyl)amino)quinazolin-6-yl)imidazo[1,2-a]pyridine-2-carboxylate **10s** as white solid (48.6 % yield).

2.3.20. Procedure for the preparation of methyl 6-(4-(methyl(p-tolyl)amino)quinazolin-6-yl)imidazo[1,2-a]pyridine-2-carboxylate (**10t**).

6-(6-aminopyridin-3-yl)-N-methyl-N-(p-tolyl)quinazolin-4-amine **7n** (0.5 mmol), methyl bromopyruvate (1.5 mmol) and $NaHCO_3$ (1.5 mmol) was added to EtOH (5 mL) and the mixture was warmed to 80 °C and refluxed by condensation under argon for 4

hours. After completion of the reaction (monitored by TLC), the solvents were removed under reduced pressure and the residue was purified through a column chromatography on silica with dichloromethane/methanol to obtain methyl 6-(4-(methyl(p-tolyl)amino)quinazolin-6-yl)imidazo[1,2-a]pyridine-2-carboxylate **10t** as pink flocculent (39.5 % yield).

2.3.21. Procedure for the preparation of methyl 6-(4-(ethyl(phenyl)amino)quinazolin-6-yl)imidazo[1,2-a]pyridine-2-carboxylate (**10u**).

6-(6-aminopyridin-3-yl)-N-ethyl-N-phenylquinazolin-4-amine **7o** (0.5 mmol), methyl bromopyruvate (1.5 mmol) and $NaHCO_3$ (1.5 mmol) was added to EtOH (5 mL) and the mixture was warmed to 80 °C and refluxed by condensation under argon for 4 hours. After completion of the reaction (monitored by TLC), the solvents were removed under reduced pressure and the residue was purified through a column chromatography on silica with dichloromethane/methanol to obtain methyl 6-(4-(ethyl(phenyl)amino)quinazolin-6-yl)imidazo[1,2-a]pyridine-2-carboxylate **10u** as orange flocculent (51.4 % yield).

3. Synthesis of compounds 13a-k

3.1. General experimental protocol for the preparation of intermediates **12a-c**.

3.1.1. Procedure for the preparation of 2-phenyl-6-(4,4,5,5-tetramethyl-1,3,2-dioxaborolan-2-yl)imidazo[1,2-a]pyridine (**12a**).

5-(4,4,5,5-Tetramethyl-1,3,2-dioxaborolan-2-yl)pyridin-2-amine (5.4 mmol), 2-bromoacetophenone (6.5 mmol) and $NaHCO_3$ (16 mmol) were added to EtOH (10 mL) and the mixture was heated to 80 °C and refluxed by condensation under argon for 4 hours. After completion of the reaction (monitored by TLC), the solvent of the reaction mixture was removed under reduced pressure and the mixture was extracted 2-3 times with ethyl acetate and saturated Na_2CO_3 solution. The organic phase was dried over anhydrous Na_2SO_4 and rotary dried under vacuum to form 2-phenyl-6-(4,4,5,5-tetramethyl-1,3,2-dioxaborolan-2-yl)imidazo[1,2-a]pyridine **12a** as pale yellow oil

substance (86.8 % yield), ESI-MS: m/z 321.1 $[M + H]^+$.

3.1.2. Procedure for the preparation of 2-(4-fluorophenyl)-6-(4,4,5,5-tetramethyl-1,3,2-dioxaborolan-2-yl)imidazo[1,2-a]pyridine (**12b**).

5-(4,4,5,5-Tetramethyl-1,3,2-dioxaborolan-2-yl)pyridin-2-amine (5.4 mmol), 2-bromo-4'-fluoroacetophenone (6.5 mmol) and $NaHCO_3$ (16 mmol) were added to EtOH (10 mL) and the mixture was heated to 80 °C and refluxed by condensation under argon for 4 hours. After completion of the reaction (monitored by TLC), the solvent of the reaction mixture was removed under reduced pressure and the mixture was extracted 2-3 times with ethyl acetate and saturated Na_2CO_3 solution. The organic phase was dried over anhydrous Na_2SO_4 and rotary dried under vacuum to form 2-(4-fluorophenyl)-6-(4,4,5,5-tetramethyl-1,3,2-dioxaborolan-2-yl)imidazo[1,2-a]pyridine **12b** as yellow solid (81.5 % yield), ESI-MS: m/z 337.2 $[M + H]^+$.

3.1.3. Procedure for the preparation of 2-cyclopropyl-6-(4,4,5,5-tetramethyl-1,3,2-dioxaborolan-2-yl)imidazo[1,2-a]pyridine (**12c**).

5-(4,4,5,5-Tetramethyl-1,3,2-dioxaborolan-2-yl)pyridin-2-amine (5.4 mmol), 2-bromo-1-cyclopropylethanone (6.5 mmol) and $NaHCO_3$ (16 mmol) were added to EtOH (10 mL) and the mixture was heated to 80 °C and refluxed by condensation under argon for 4 hours. After completion of the reaction (monitored by TLC), the solvent of the reaction mixture was removed under reduced pressure and the mixture was extracted 2-3 times with ethyl acetate and saturated Na_2CO_3 solution. The organic phase was dried over anhydrous Na_2SO_4 and rotary dried under vacuum to form 2-cyclopropyl-6-(4,4,5,5-tetramethyl-1,3,2-dioxaborolan-2-yl)imidazo[1,2-a]pyridine **12c** as yellow solid (81.5 % yield), ESI-MS: m/z 283.2 $[M + H]^+$.

3.2. General experimental protocol for preparation of compounds **13a-13k**

3.2.1. Procedure for the preparation of N-(2-fluorobenzyl)-6-(2-phenylimidazo[1,2-a]pyridin-6-yl)quinazolin-4-amine (**13a**).

N-(2-fluorobenzyl)-6-iodoquinazolin-4-amine **5i** (0.5mmol), 2-phenyl-6-(4,4,5,5-tetramethyl-1,3,2-dioxaborolan-2-yl)imidazo[1,2-a]pyridine **12a** (0.5 mmol) and K_2CO_3 (1.5 mmol) were added to 1,4-dioxane/water 10 mL $[V_{(1,4\text{-dioxane})}:V_{(water)} = 4:1]$ and the mixture was heated to 100 °C under a protective atmosphere of argon followed

by the addition of Pd(dppf)Cl_2. The mixture continues to be stirred under these conditions for a further 4-5 hours. After completion of the reaction (monitored by TLC), the 1, 4-dioxane and water were removed under reduced pressure and the residue was purified through a column chromatography on silica with dichloromethane/methanol to afford N-(2-fluorobenzyl)-6-(2-phenylimidazo[1,2-a]pyridin-6-yl)quinazolin-4-amine **13a** as pink flocculent (67.3 % yield).

3.2.2. Procedure for the preparation of N-(2-fluorobenzyl)-6-(2-(4-fluorophenyl)imidazo[1,2-a]pyridin-6-yl)quinazolin-4-amine (**13b**).

N-(2-fluorobenzyl)-6-iodoquinazolin-4-amine **5i** (0.5mmol), 2-(4-fluorophenyl)-6-(4,4,5,5-tetramethyl-1,3,2-dioxaborolan-2-yl)imidazo[1,2-a]pyridine **12b** (0.5 mmol) and K_2CO_3 (1.5 mmol) were added to 1,4-dioxane/water 10 mL [$V_{(1,4\text{-dioxane})}$:$V_{(water)}$ = 4:1] and the mixture was heated to 100 °C under a protective atmosphere of argon followed by the addition of Pd(dppf)Cl_2. The mixture continues to be stirred under these conditions for a further 4-5 hours. After completion of the reaction (monitored by TLC), the 1, 4-dioxane and water were removed under reduced pressure and the residue was purified through a column chromatography on silica with dichloromethane/methanol to afford N-(2-fluorobenzyl)-6-(2-(4-fluorophenyl)imidazo[1,2-a]pyridin-6-yl)quinazolin-4-amine **13b** as pink flocculent (70.7 % yield).

3.2.3. Procedure for the preparation of N-(2,3-difluorophenyl)-6-(2-phenylimidazo[1,2-a]pyridin-6-yl)quinazolin-4-amine (**13c**).

N-(2,3-difluorophenyl)-6-iodoquinazolin-4-amine **5m** (0.5mmol), 2-phenyl-6-(4,4,5,5-tetramethyl-1,3,2-dioxaborolan-2-yl)imidazo[1,2-a]pyridine **12a** (0.5 mmol) and K_2CO_3 (1.5 mmol) were added to 1,4-dioxane/water 10 mL [$V_{(1,4\text{-dioxane})}$:$V_{(water)}$ = 4:1] and the mixture was heated to 100 °C under a protective atmosphere of argon followed by the addition of Pd(dppf)Cl_2. The mixture continues to be stirred under these conditions for a further 4-5 hours. After completion of the reaction (monitored by TLC), the 1, 4-dioxane and water were removed under reduced pressure and the residue was purified through a column chromatography on silica with dichloromethane/methanol to afford N-(2,3-difluorophenyl)-6-(2-phenylimidazo[1,2-a]pyridin-6-yl)quinazolin-4-

amine **13c** as pink flocculent (73.2 % yield).

3.2.4. Procedure for the preparation of N-(2,3-difluorophenyl)-6-(2-(4-fluorophenyl)imidazo[1,2-a]pyridin-6-yl)quinazolin-4-amine (**13d**).

N-(2,3-difluorophenyl)-6-iodoquinazolin-4-amine **5m** (0.5mmol), 2-(4-fluorophenyl)-6-(4,4,5,5-tetramethyl-1,3,2-dioxaborolan-2-yl)imidazo[1,2-a]pyridine **12b** (0.5 mmol) and K_2CO_3 (1.5 mmol) were added to 1,4-dioxane/water 10 mL [$V_{(1,4\text{-dioxane})}$:$V_{(water)}$ = 4:1] and the mixture was heated to 100 °C under a protective atmosphere of argon followed by the addition of $Pd(dppf)Cl_2$. The mixture continues to be stirred under these conditions for a further 4-5 hours. After completion of the reaction (monitored by TLC), the 1, 4-dioxane and water were removed under reduced pressure and the residue was purified through a column chromatography on silica with dichloromethane/methanol to afford N-(2,3-difluorophenyl)-6-(2-(4-fluorophenyl)imidazo[1,2-a]pyridin-6-yl)quinazolin-4-amine **13d** as pink flocculent (72.5 % yield).

3.2.5. Procedure for the preparation of N-(cyclopropylmethyl)-6-(2-(4-fluorophenyl)imidazo[1,2-a]pyridin-6-yl)quinazolin-4-amine (**13e**).

N-(cyclopropylmethyl)-6-iodoquinazolin-4-amine **5b** (0.5mmol), 2-(4-fluorophenyl)-6-(4,4,5,5-tetramethyl-1,3,2-dioxaborolan-2-yl)imidazo[1,2-a]pyridine **12b** (0.5 mmol) and K_2CO_3 (1.5 mmol) were added to 1,4-dioxane/water 10 mL [$V_{(1,4\text{-dioxane})}$:$V_{(water)}$ = 4:1] and the mixture was heated to 100 °C under a protective atmosphere of argon followed by the addition of $Pd(dppf)Cl_2$. The mixture continues to be stirred under these conditions for a further 4-5 hours. After completion of the reaction (monitored by TLC), the 1, 4-dioxane and water were removed under reduced pressure and the residue was purified through a column chromatography on silica with dichloromethane/methanol to afford N-(cyclopropylmethyl)-6-(2-(4-fluorophenyl)imidazo[1,2-a]pyridin-6-yl)quinazolin-4-amine **13e** as pink flocculent (56.1 % yield).

3.2.6. Procedure for the preparation of 6-(2-phenylimidazo[1,2-a]pyridin-6-yl)-N-(1H-pyrazol-3-yl)quinazolin-4-amine (**13f**).

6-iodo-N-(1H-pyrazol-3-yl)quinazolin-4-amine **5p** (0.5mmol), 2-phenyl-6-(4,4,5,5-tetramethyl-1,3,2-dioxaborolan-2-yl)imidazo[1,2-a]pyridine **12a** (0.5 mmol) and K_2CO_3 (1.5 mmol) were added to 1,4-dioxane/water 10 mL [$V_{(1,4\text{-dioxane})}$:$V_{(water)}$ = 4:1] and the mixture was heated to 100 °C under a protective atmosphere of argon followed by the addition of Pd(dppf)Cl_2. The mixture continues to be stirred under these conditions for a further 4-5 hours. After completion of the reaction (monitored by TLC), the 1, 4-dioxane and water were removed under reduced pressure and the residue was purified through a column chromatography on silica with dichloromethane/methanol to afford 6-(2-phenylimidazo[1,2-a]pyridin-6-yl)-N-(1H-pyrazol-3-yl)quinazolin-4-amine **13f** as pink solid (54.6 % yield).

3.2.7. Procedure for the preparation of 6-(2-cyclopropylimidazo[1,2-a]pyridin-6-yl)-N-(cyclopropylmethyl)quinazolin-4-amine (**13g**).

N-(cyclopropylmethyl)-6-iodoquinazolin-4-amine **5b** (0.5mmol), 2-cyclopropyl-6-(4,4,5,5-tetramethyl-1,3,2-dioxaborolan-2-yl)imidazo[1,2-a]pyridine **12c** (0.5 mmol) and K_2CO_3 (1.5 mmol) were added to 1,4-dioxane/water 10 mL [$V_{(1,4\text{-dioxane})}$:$V_{(water)}$ = 4:1] and the mixture was heated to 100 °C under a protective atmosphere of argon followed by the addition of Pd(dppf)Cl_2. The mixture continues to be stirred under these conditions for a further 4-5 hours. After completion of the reaction (monitored by TLC), the 1, 4-dioxane and water were removed under reduced pressure and the residue was purified through a column chromatography on silica with dichloromethane/methanol to afford 6-(2-cyclopropylimidazo[1,2-a]pyridin-6-yl)-N-(cyclopropylmethyl)quinazolin-4-amine **13g** as yellow solid (60.6 % yield).

3.2.8. Procedure for the preparation of 6-(2-cyclopropylimidazo[1,2-a]pyridin-6-yl)-N-ethyl-N-phenylquinazolin-4-amine (**13h**).

N-ethyl-6-iodo-N-phenylquinazolin-4-amine **5o** (0.5mmol), 2-cyclopropyl-6-(4,4,5,5-tetramethyl-1,3,2-dioxaborolan-2-yl)imidazo[1,2-a]pyridine **12c** (0.5 mmol) and K_2CO_3 (1.5 mmol) were added to 1,4-dioxane/water 10 mL [$V_{(1,4\text{-dioxane})}$:$V_{(water)}$ = 4:1] and the mixture was heated to 100 °C under a protective atmosphere of argon followed by the addition of Pd(dppf)Cl_2. The mixture continues to be stirred under these

conditions for a further 4-5 hours. After completion of the reaction (monitored by TLC), the 1, 4-dioxane and water were removed under reduced pressure and the residue was purified through a column chromatography on silica with dichloromethane/methanol to afford 6-(2-cyclopropylimidazo[1,2-a]pyridin-6-yl)-N-ethyl-N-phenylquinazolin-4-amine **13h** as yellow solid (58.3 % yield).

3.2.9. Procedure for the preparation of N-ethyl-N-phenyl-6-(2-phenylimidazo[1,2-a]pyridin-6-yl)quinazolin-4-amine (**13i**).

N-ethyl-6-iodo-N-phenylquinazolin-4-amine **5o** (0.5mmol), 2-phenyl-6-(4,4,5,5-tetramethyl-1,3,2-dioxaborolan-2-yl)imidazo[1,2-a]pyridine **12a** (0.5 mmol) and K_2CO_3 (1.5 mmol) were added to 1,4-dioxane/water 10 mL [$V_{(1,4\text{-dioxane})}$:$V_{(water)}$ = 4:1] and the mixture was heated to 100 °C under a protective atmosphere of argon followed by the addition of $Pd(dppf)Cl_2$. The mixture continues to be stirred under these conditions for a further 4-5 hours. After completion of the reaction (monitored by TLC), the 1, 4-dioxane and water were removed under reduced pressure and the residue was purified through a column chromatography on silica with dichloromethane/methanol to afford N-ethyl-N-phenyl-6-(2-phenylimidazo[1,2-a]pyridin-6-yl)quinazolin-4-amine **13i** as pink solid (67.4 % yield).

3.2.10. Procedure for the preparation of 6-(2-phenylimidazo[1,2-a]pyridin-6-yl)-N-(pyridin-2-ylmethyl)quinazolin-4-amine (**13j**).

6-iodo-N-(pyridin-2-ylmethyl)quinazolin-4-amine **5h** (0.5mmol), 2-phenyl-6-(4,4,5,5-tetramethyl-1,3,2-dioxaborolan-2-yl)imidazo[1,2-a]pyridine **12a** (0.5 mmol) and K_2CO_3 (1.5 mmol) were added to 1,4-dioxane/water 10 mL [$V_{(1,4\text{-dioxane})}$:$V_{(water)}$ = 4:1] and the mixture was heated to 100 °C under a protective atmosphere of argon followed by the addition of $Pd(dppf)Cl_2$. The mixture continues to be stirred under these conditions for a further 4-5 hours. After completion of the reaction (monitored by TLC), the 1, 4-dioxane and water were removed under reduced pressure and the residue was purified through a column chromatography on silica with dichloromethane/methanol to afford 6-(2-phenylimidazo[1,2-a]pyridin-6-yl)-N-(pyridin-2-ylmethyl)quinazolin-4-amine **13j** as pink solid (70.2 % yield).

3.2.11. Procedure for the preparation of 6-(2-phenylimidazo[1,2-a]pyridin-6-yl)-N-((tetrahydro-2H-pyran-4-yl)methyl)quinazolin-4-amine (**13k**).

6-iodo-N-((tetrahydro-2H-pyran-4-yl)methyl)quinazolin-4-amine **5q** (0.5mmol), 2-phenyl-6-(4,4,5,5-tetramethyl-1,3,2-dioxaborolan-2-yl)imidazo[1,2-a]pyridine **12a** (0.5 mmol) and K_2CO_3 (1.5 mmol) were added to 1,4-dioxane/water 10 mL [V $_{(1,4\text{-dioxane})}$:V $_{(water)}$ = 4:1] and the mixture was heated to 100 °C under a protective atmosphere of argon followed by the addition of Pd(dppf)Cl_2. The mixture continues to be stirred under these conditions for a further 4-5 hours. After completion of the reaction (monitored by TLC), the 1, 4-dioxane and water were removed under reduced pressure and the residue was purified through a column chromatography on silica with dichloromethane/methanol to afford 6-(2-phenylimidazo[1,2-a]pyridin-6-yl)-N-((tetrahydro-2H-pyran-4-yl)methyl)quinazolin-4-amine **13k** as pink solid (69.8 % yield).

4. ^{1}H NMR and ^{13}C NMR Spectrum of the compounds 10a-10u.

Spectra of compound **10a** (^{1}H NMR, ^{13}C NMR, HRMS)

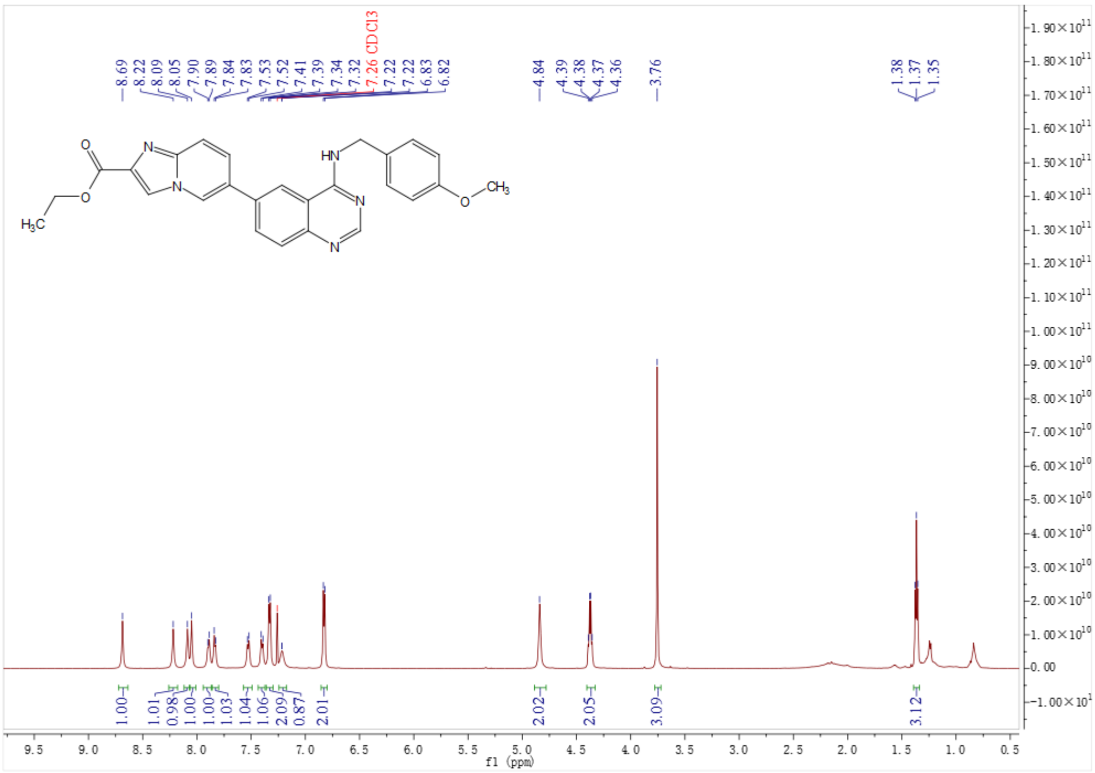

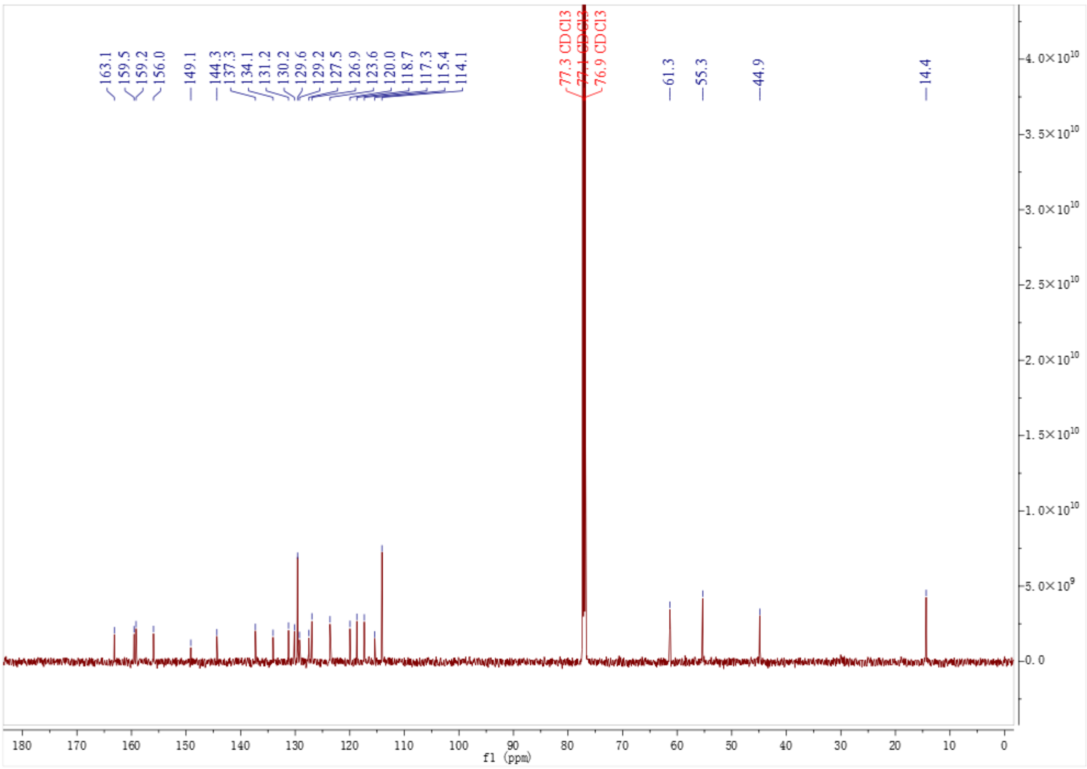
163.1
159.5
159.2
156.0
149.1
144.3
137.3
134.1
131.2
130.2
129.6
129.2
127.5
126.9
123.6
120.0
118.7
117.3
115.4
114.1
77.3 CDCl3
76.9 CDCl3
61.3
55.3
44.9
14.4
f1 (ppm)

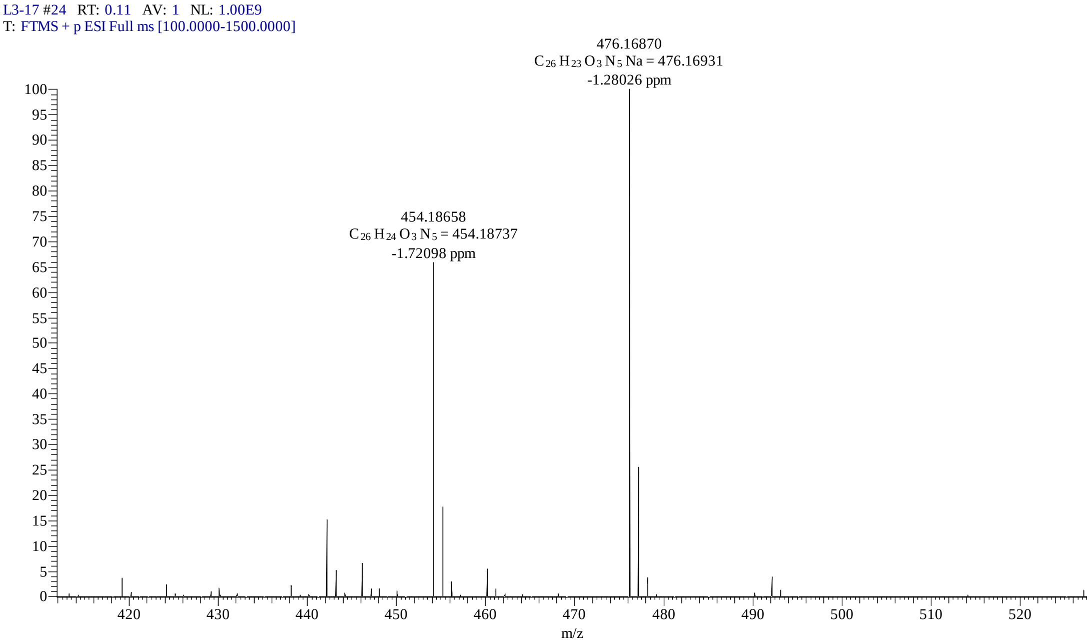
L3-17 #24 RT: 0.11 AV: 1 NL: 1.00E9
T: FTMS + p ESI Full ms [100.0000-1500.0000]
476.16870
C 26 H 23 O 3 N 5 Na = 476.16931
-1.28026 ppm
454.18658
C 26 H 24 O 3 N 5 = 454.18737
-1.72098 ppm
m/z

Spectra of compound **10b** (^{1}H NMR, ^{13}C NMR, HRMS)

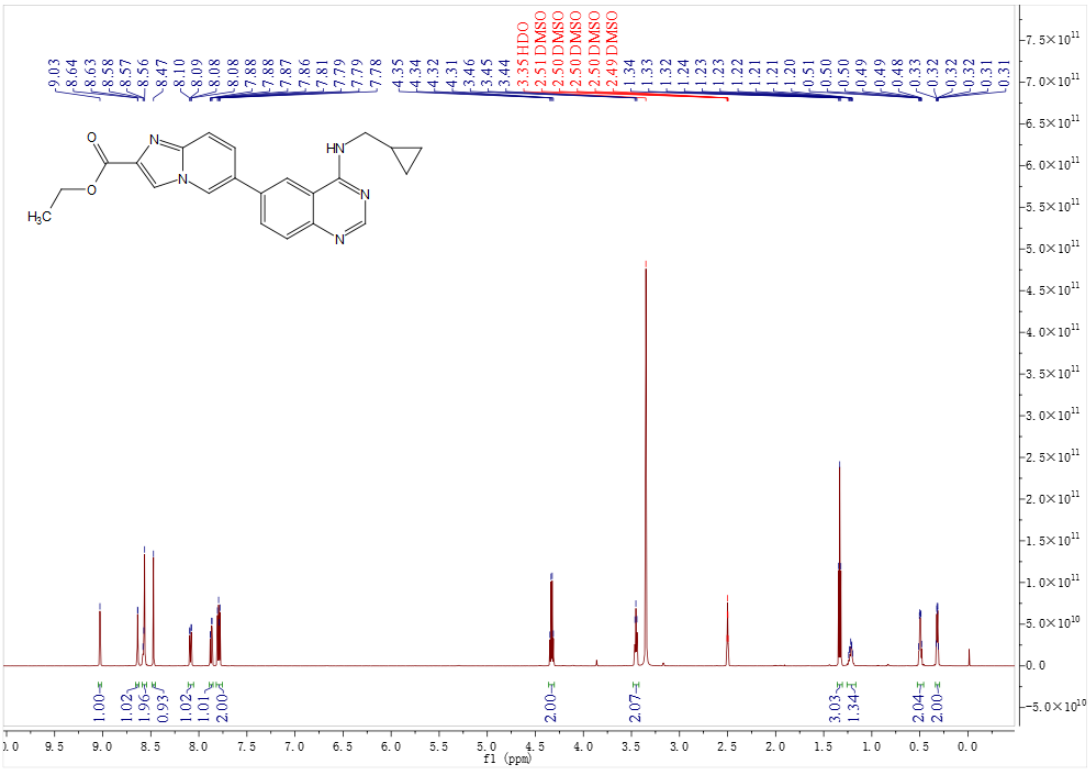

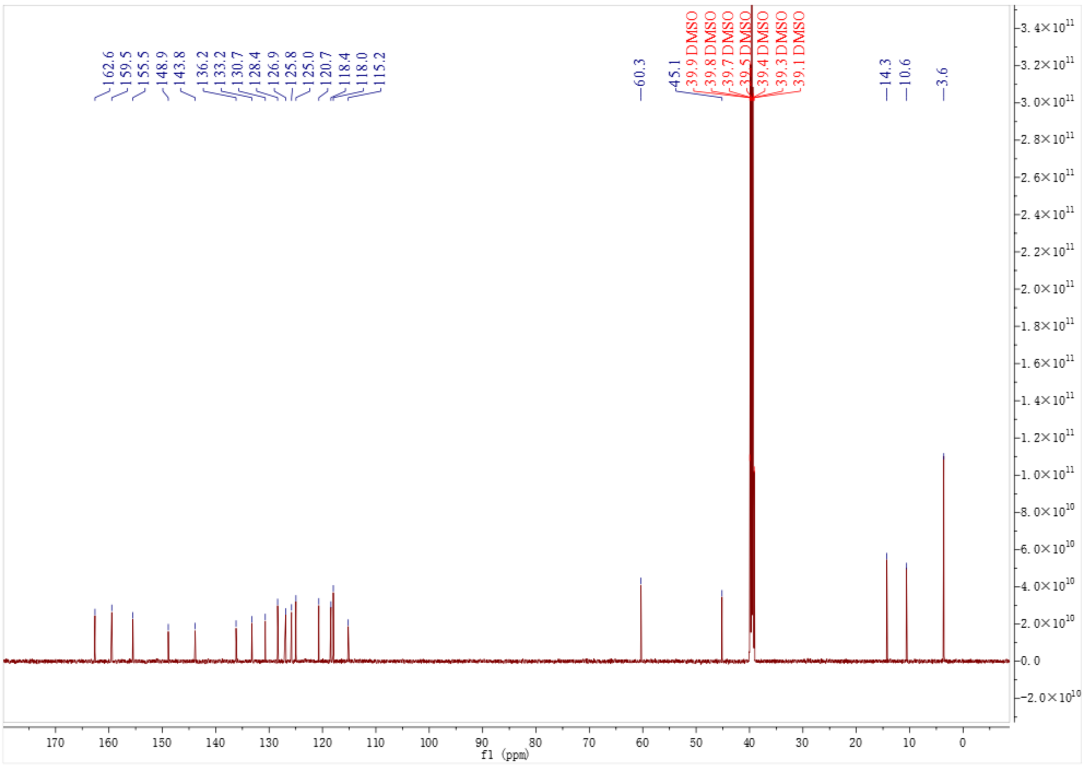

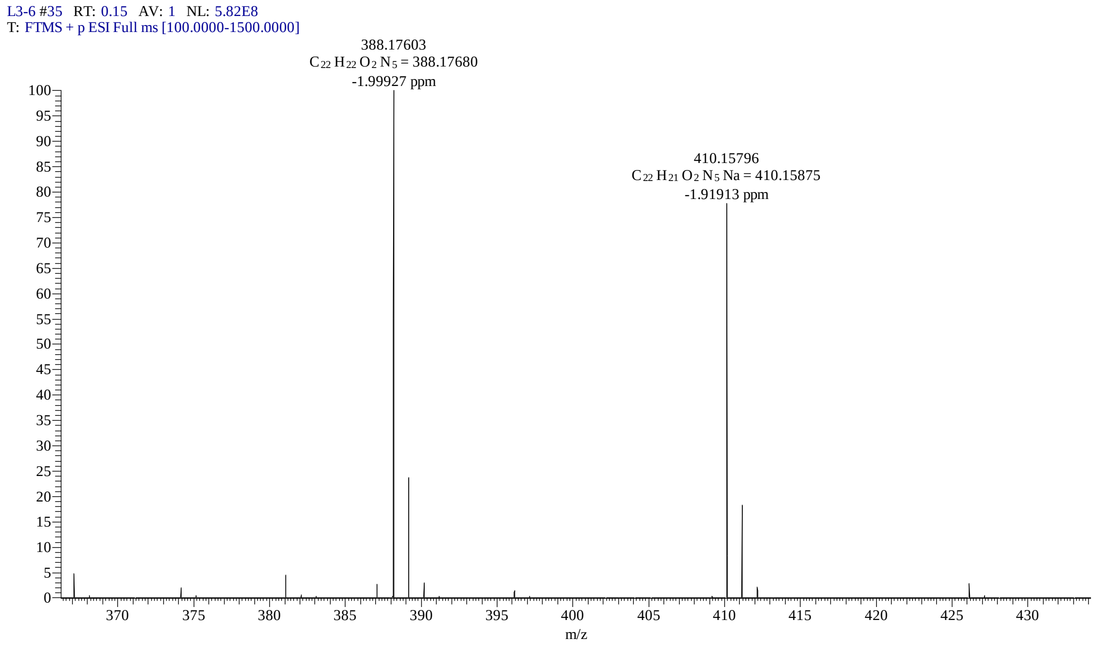

Spectra of compound **10c** (^{1}H NMR, ^{13}C NMR, ^{19}F NMR, HRMS)

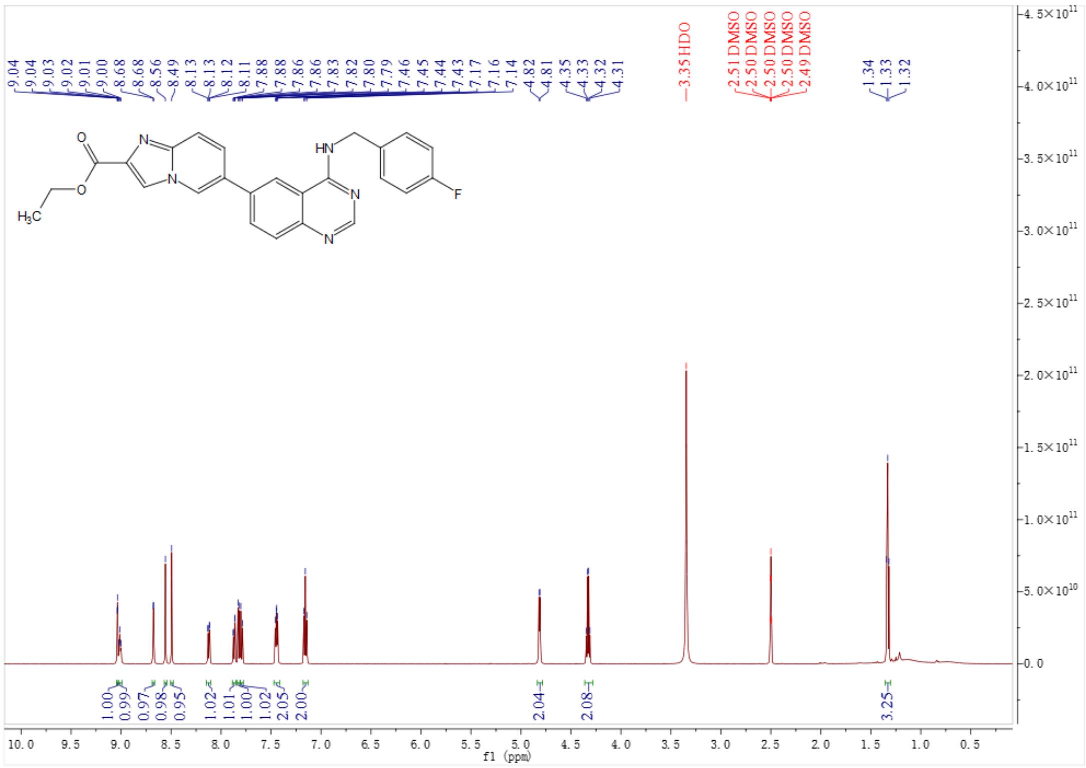

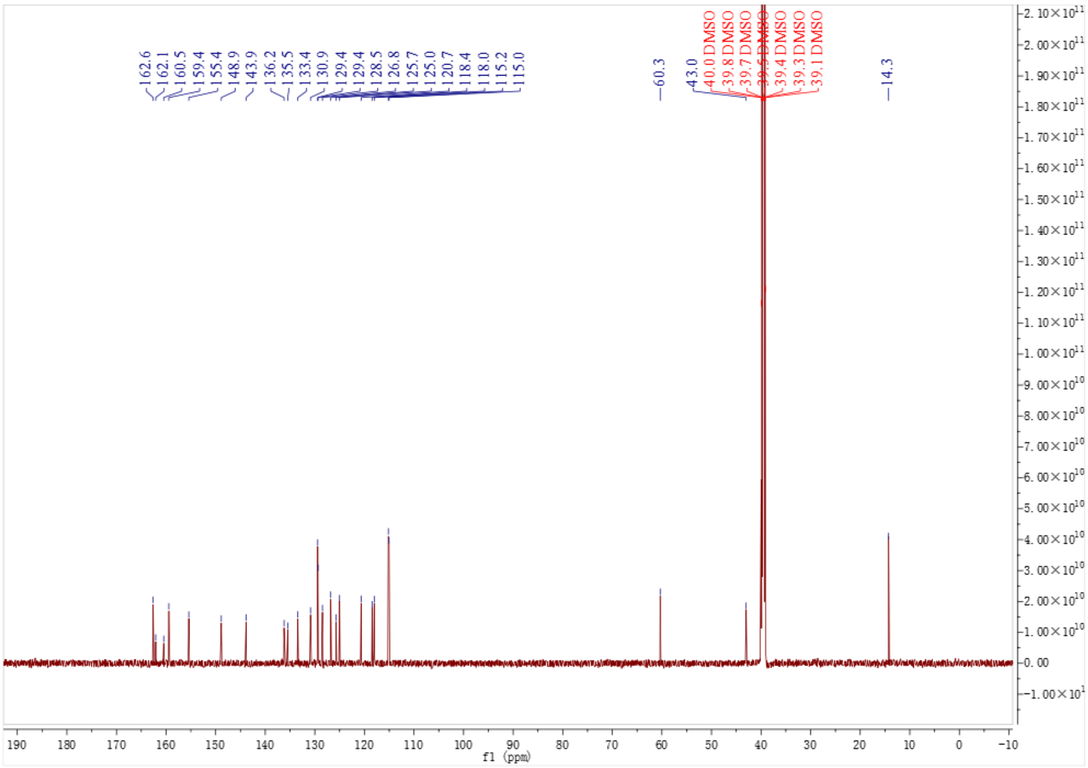
162.6
162.1
160.5
159.4
155.4
148.9
143.9
136.2
135.5
133.4
130.9
129.4
129.4
128.5
126.8
125.7
125.0
120.7
118.4
118.0
115.2
115.0
60.3
43.0
40.0 DMSO
39.8 DMSO
39.7 DMSO
39.5 DMSO
39.4 DMSO
39.3 DMSO
39.1 DMSO
14.3
f1 (ppm)

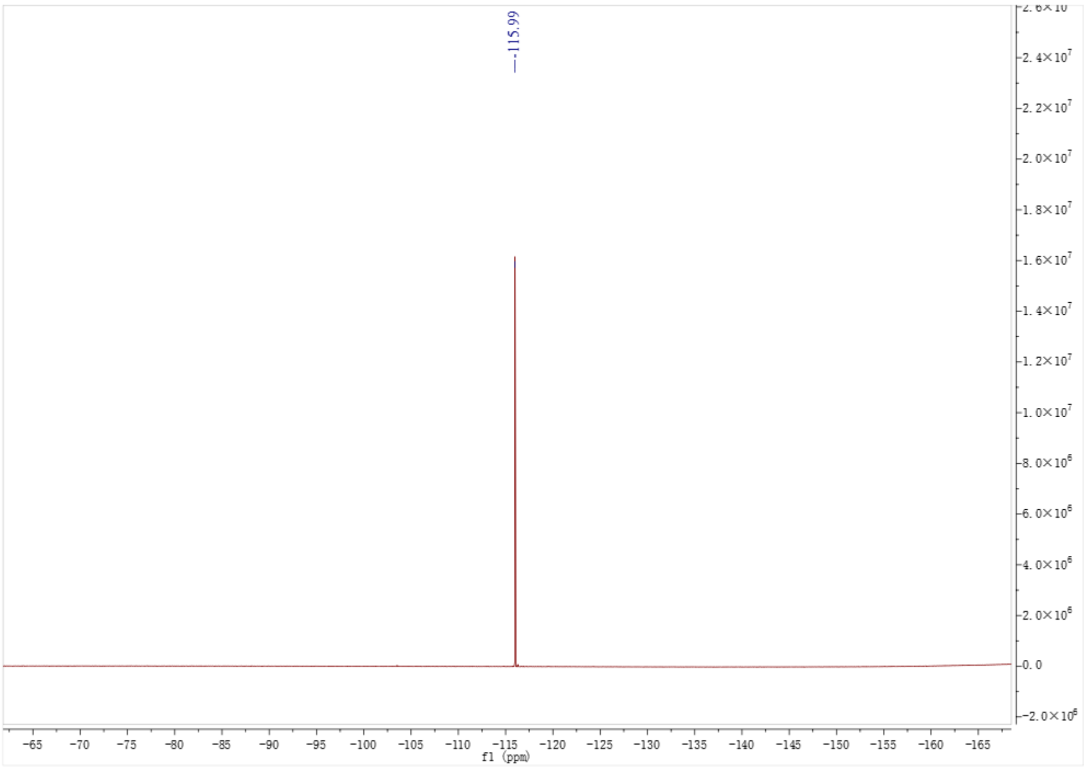
-115.99
f1 (ppm)

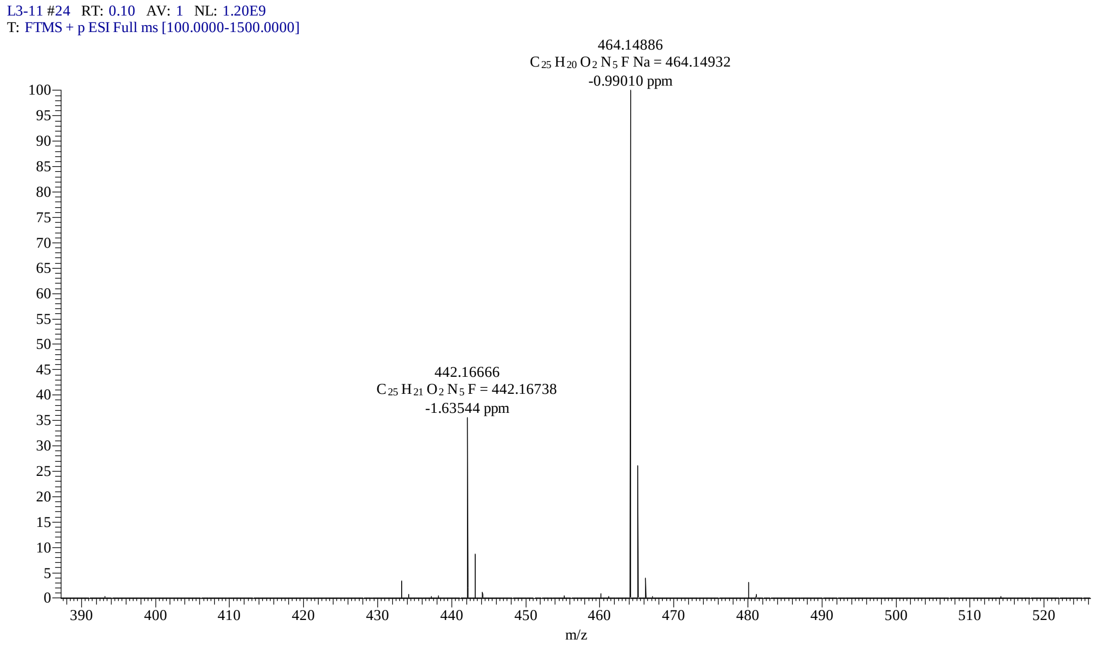

Spectra of compound **10d** (^{1}H NMR, ^{13}C NMR, ^{19}F NMR, HRMS)

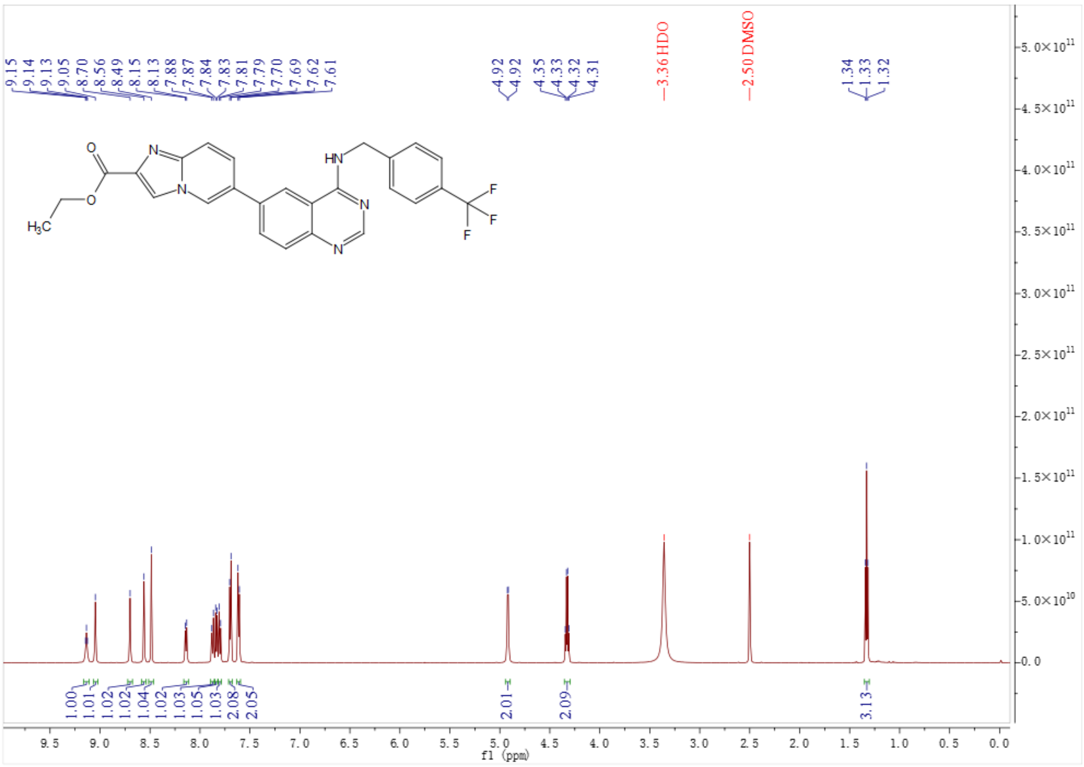

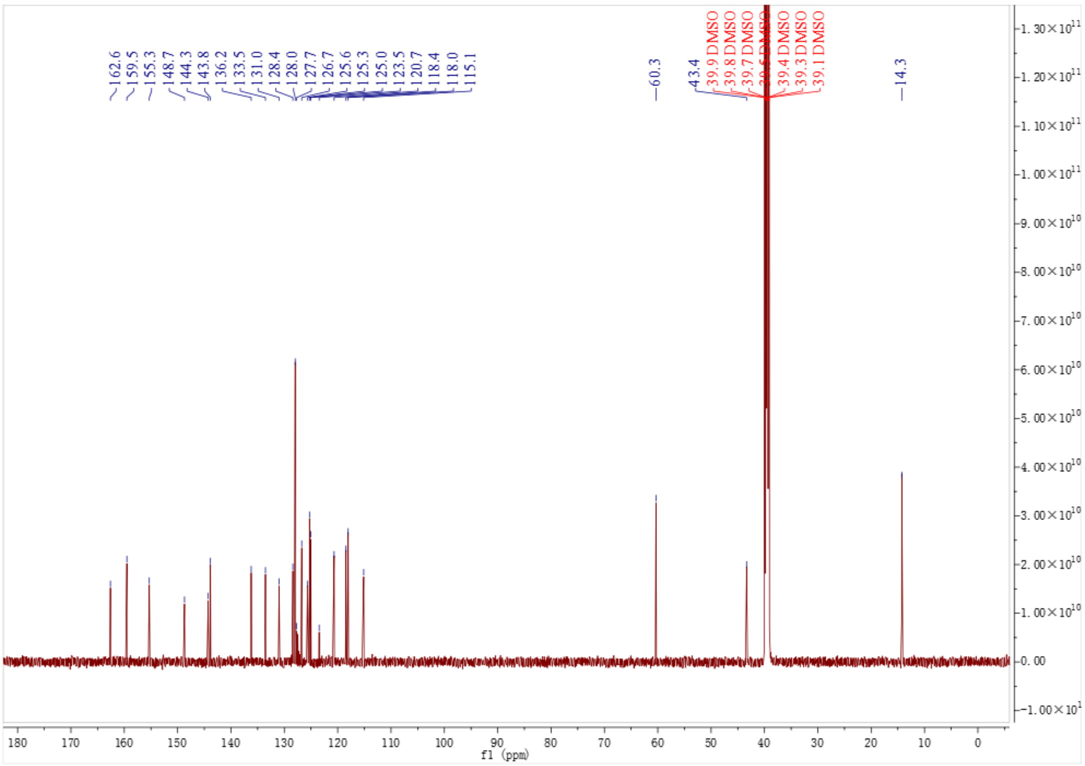
162.6
159.5
155.3
148.7
144.3
143.8
136.2
133.5
131.0
128.4
128.0
127.7
126.7
125.6
125.3
125.0
123.5
120.7
118.4
118.0
115.1
60.3
43.4
39.9 DMSO
39.8 DMSO
39.7 DMSO
39.4 DMSO
39.3 DMSO
39.1 DMSO
14.3
f1 (ppm)

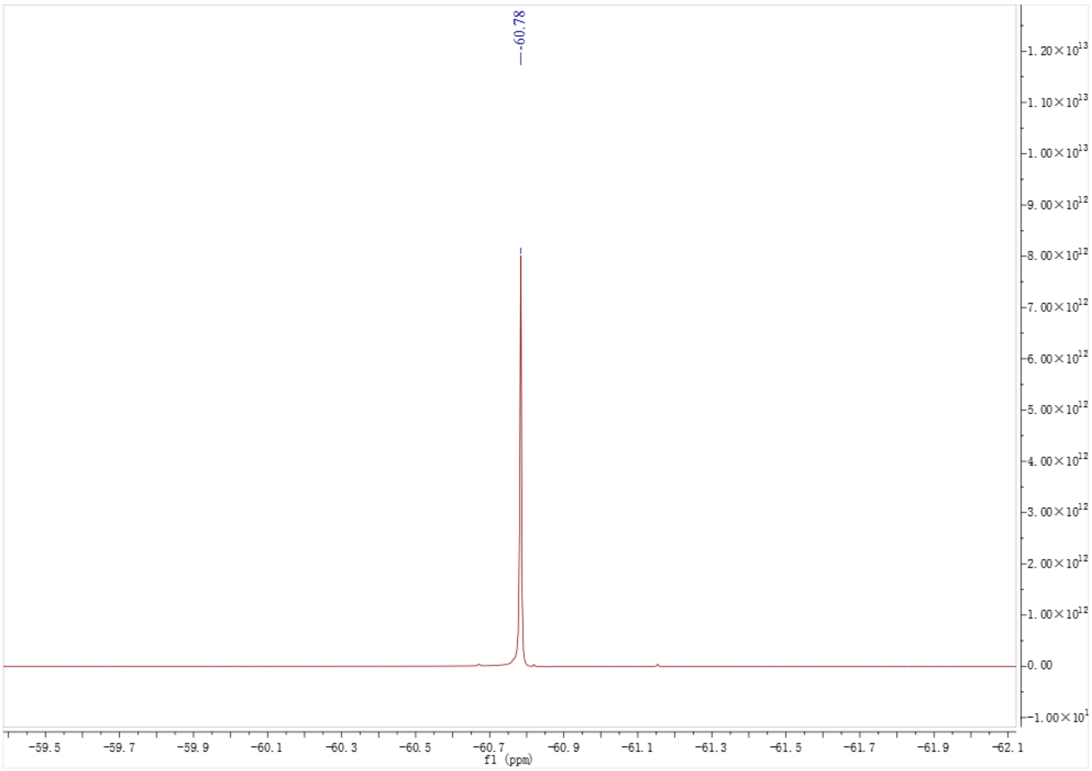
-60.78
f1 (ppm)

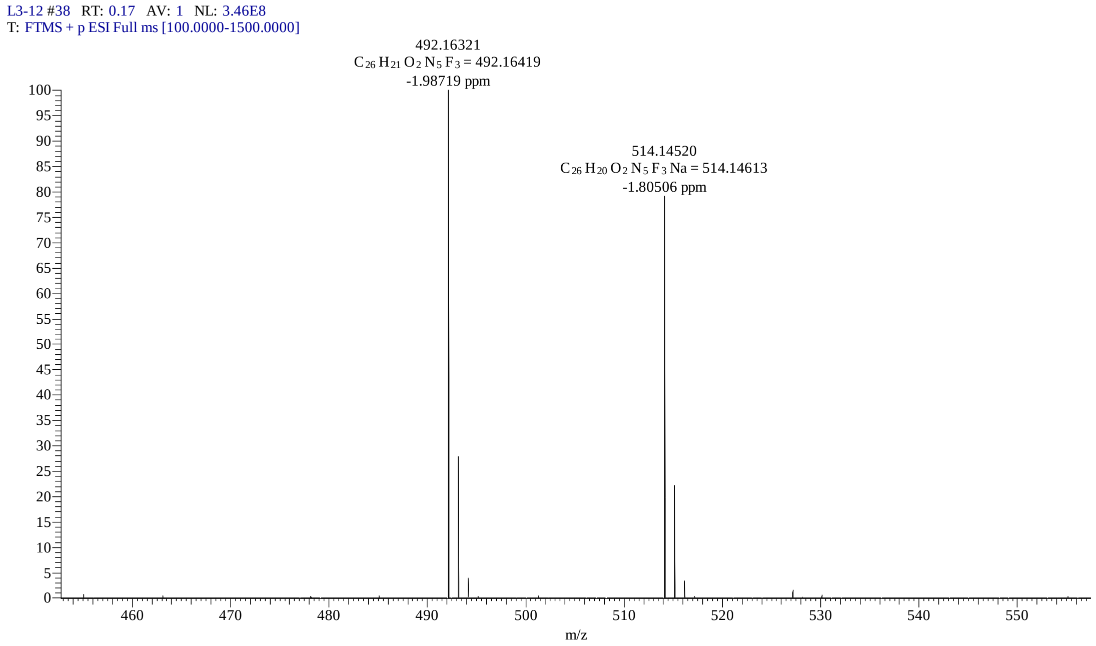

Spectra of compound **10e** (^{1}H NMR, ^{13}C NMR, HRMS)

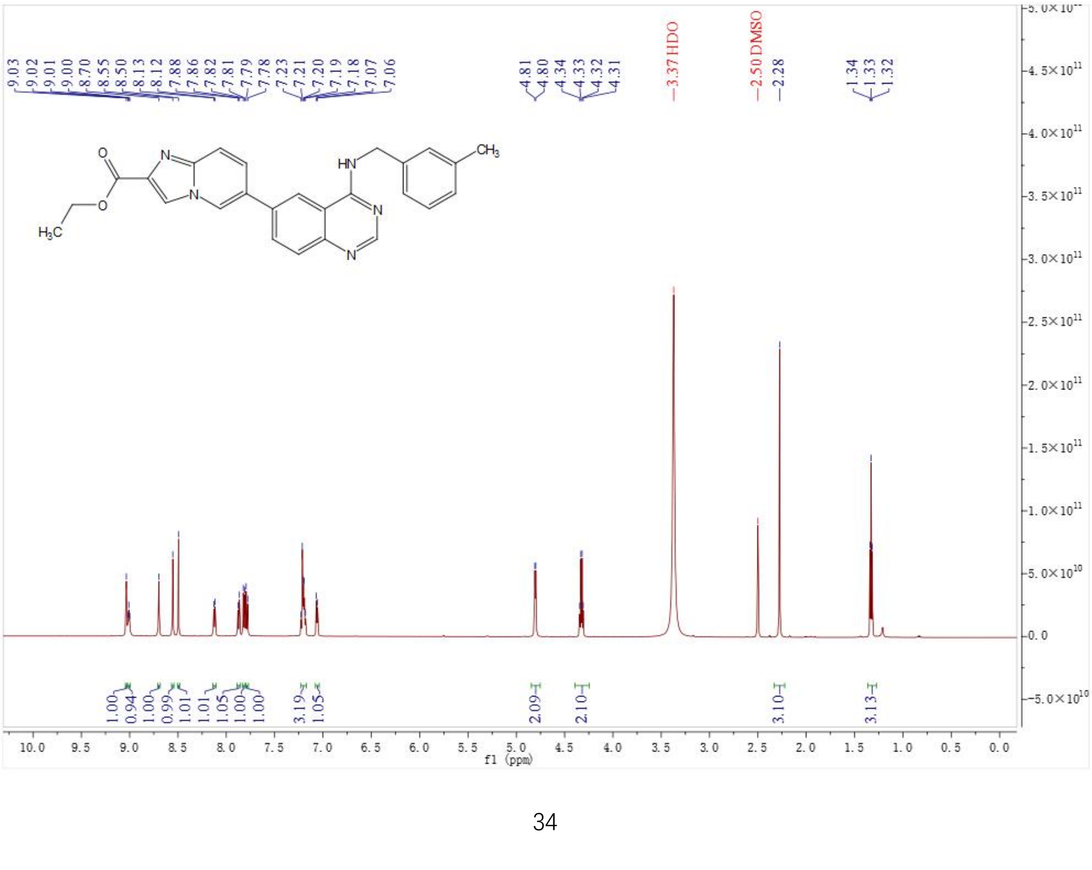

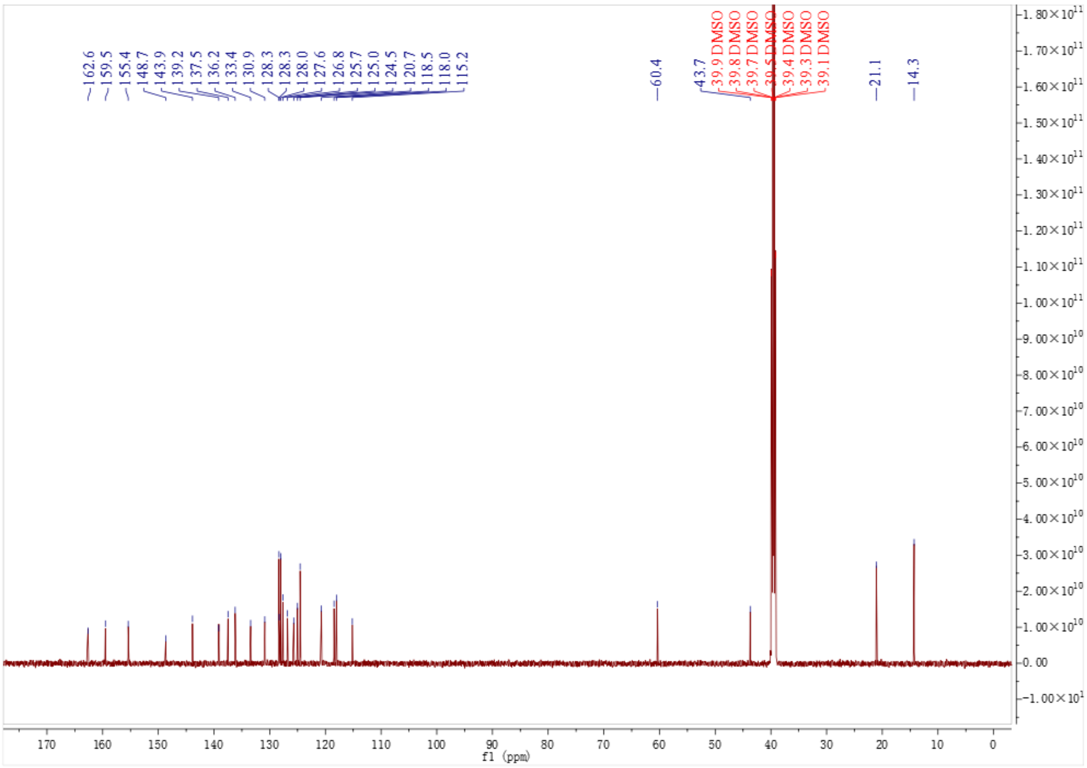

L3-5 #25 RT: 0.11 AV: 1 NL: 1.70E9
T: FTMS + p ESI Full ms [100.0000-1500.0000]

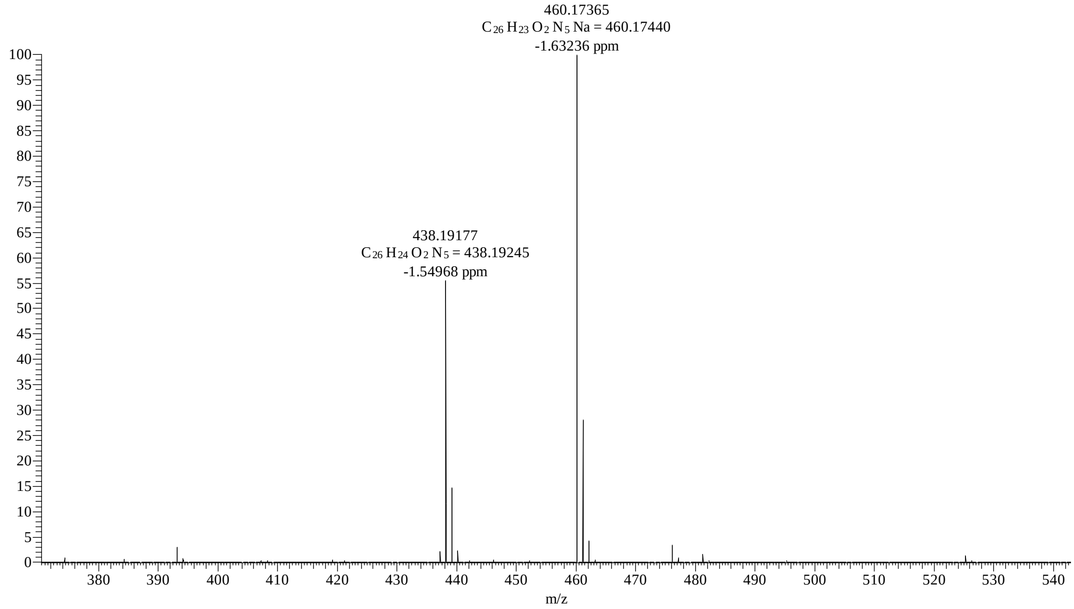

Spectra of compound **10f** (^{1}H NMR, ^{13}C NMR, HRMS)

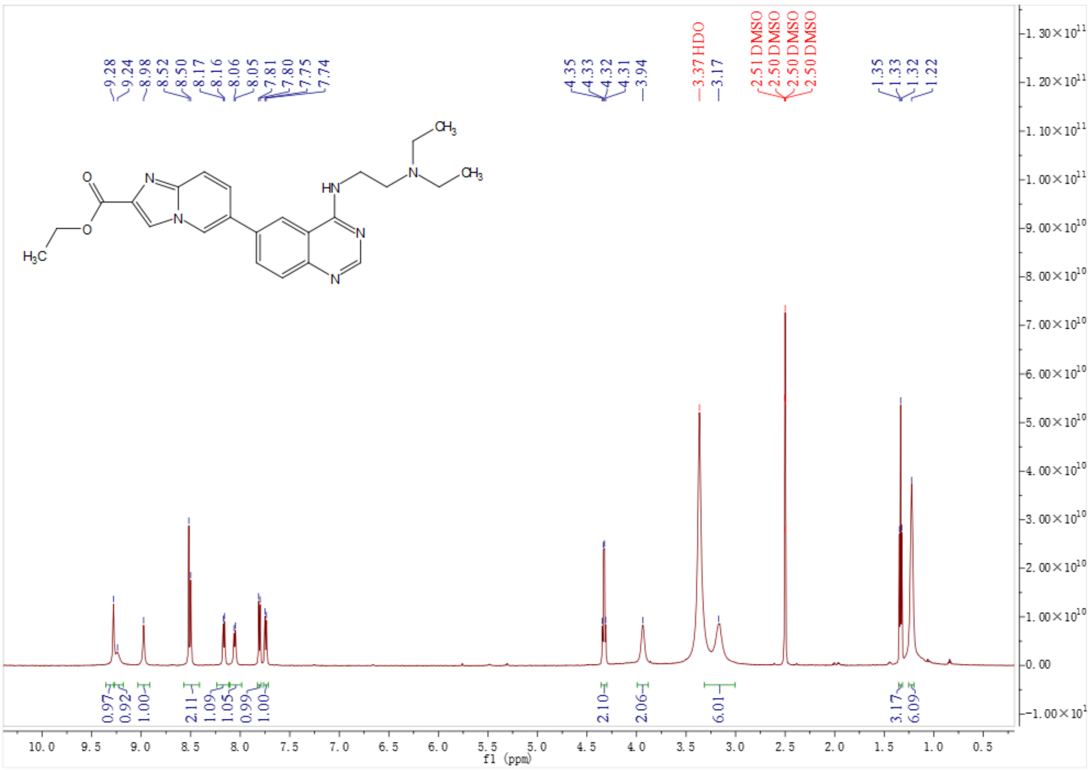

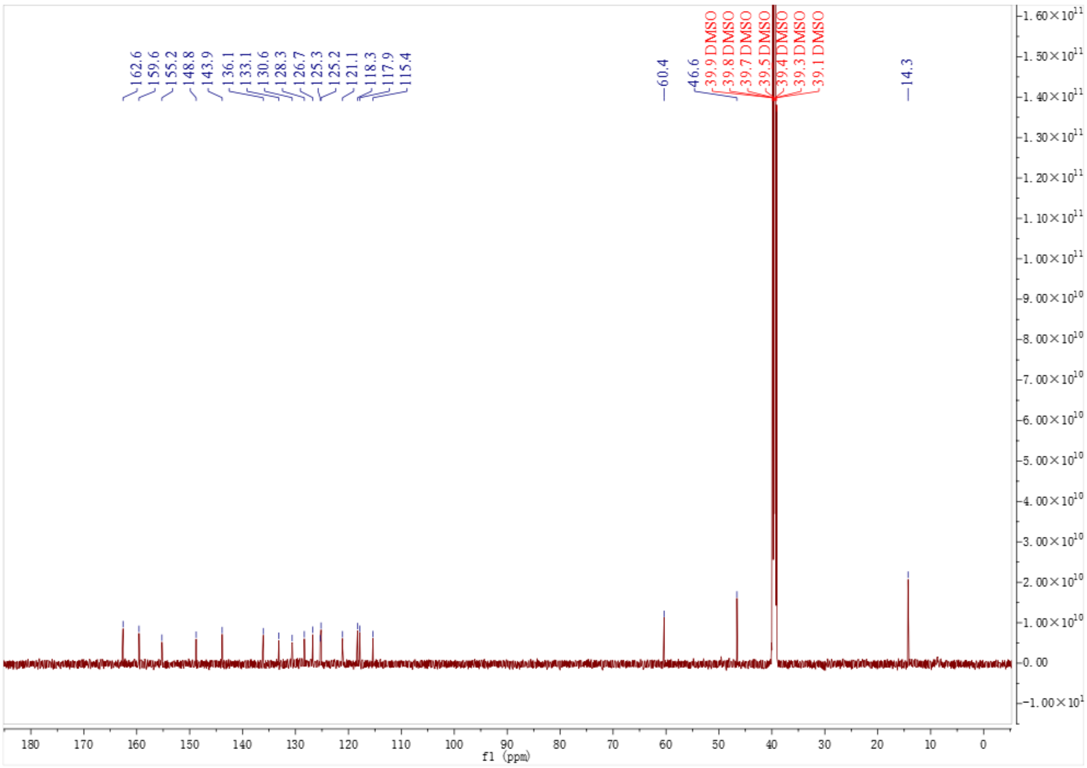

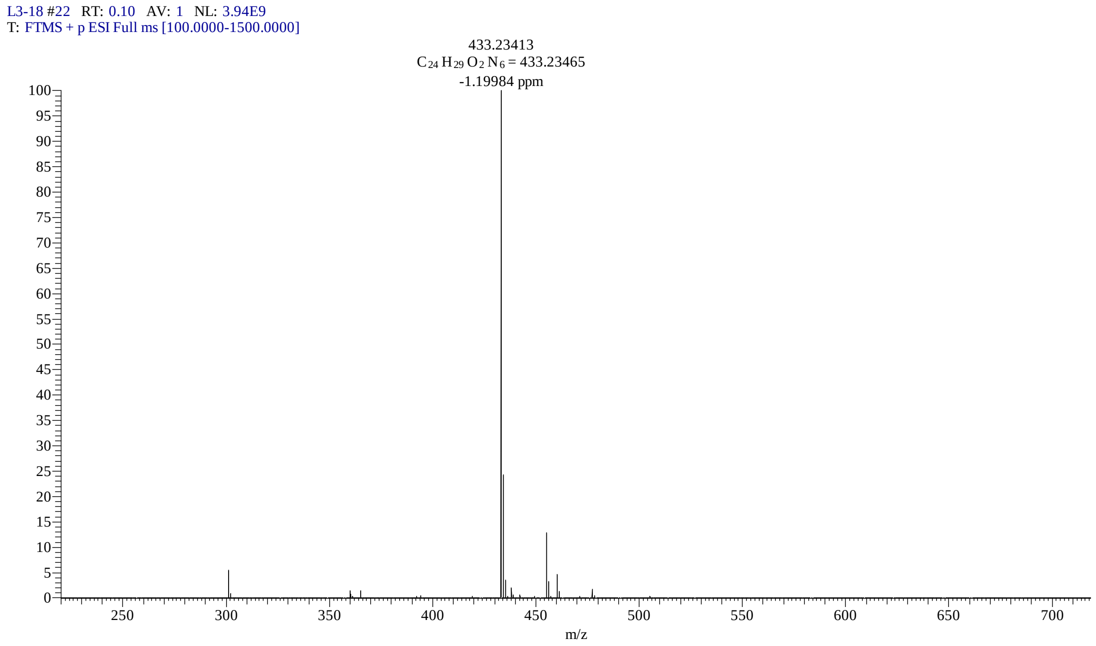

Spectra of compound **10g** (^{1}H NMR, ^{13}C NMR, HRMS)

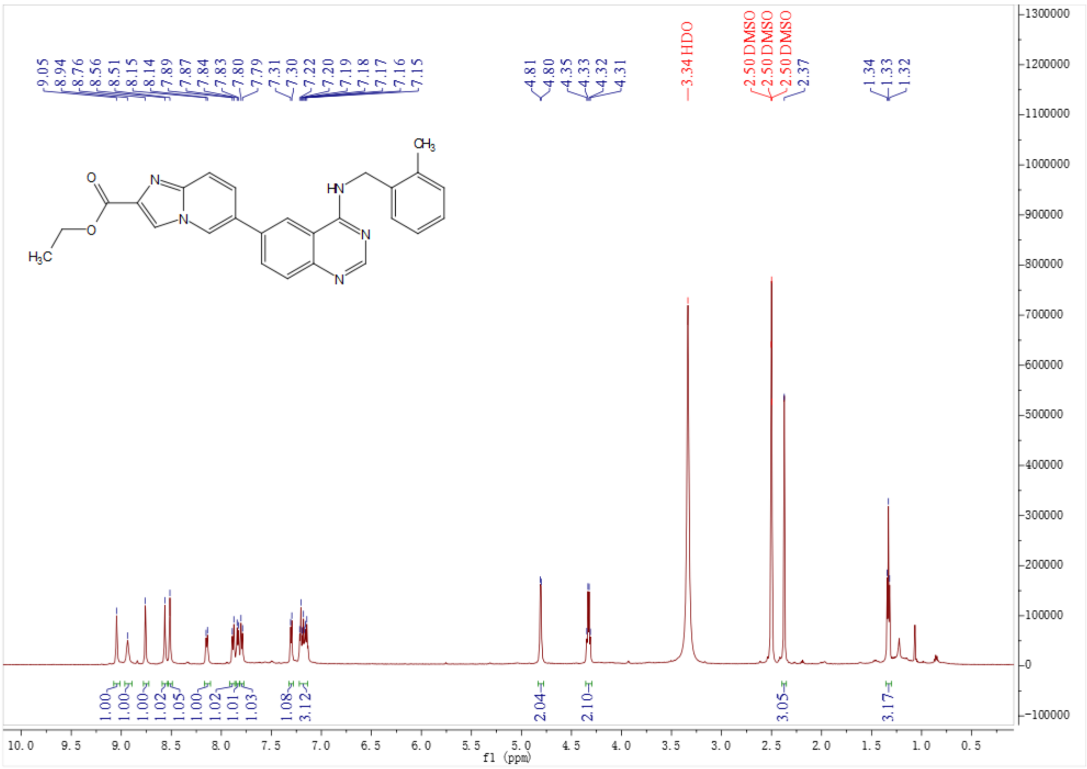

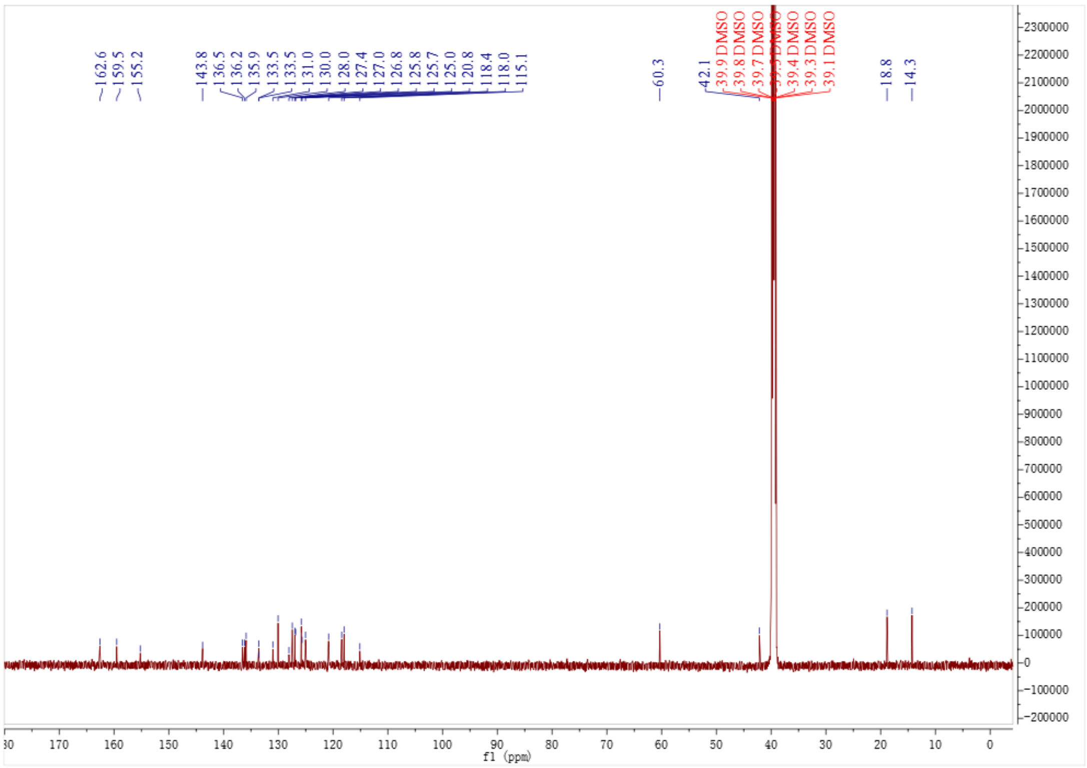
162.6
159.5
155.2
143.8
136.5
136.2
135.9
133.5
133.5
131.0
130.0
128.0
127.4
127.0
126.8
125.8
125.7
125.0
120.8
118.4
118.0
115.1
60.3
42.1
39.9 DMSO
39.8 DMSO
39.7 DMSO
39.4 DMSO
39.3 DMSO
39.1 DMSO
18.8
14.3
f1 (ppm)

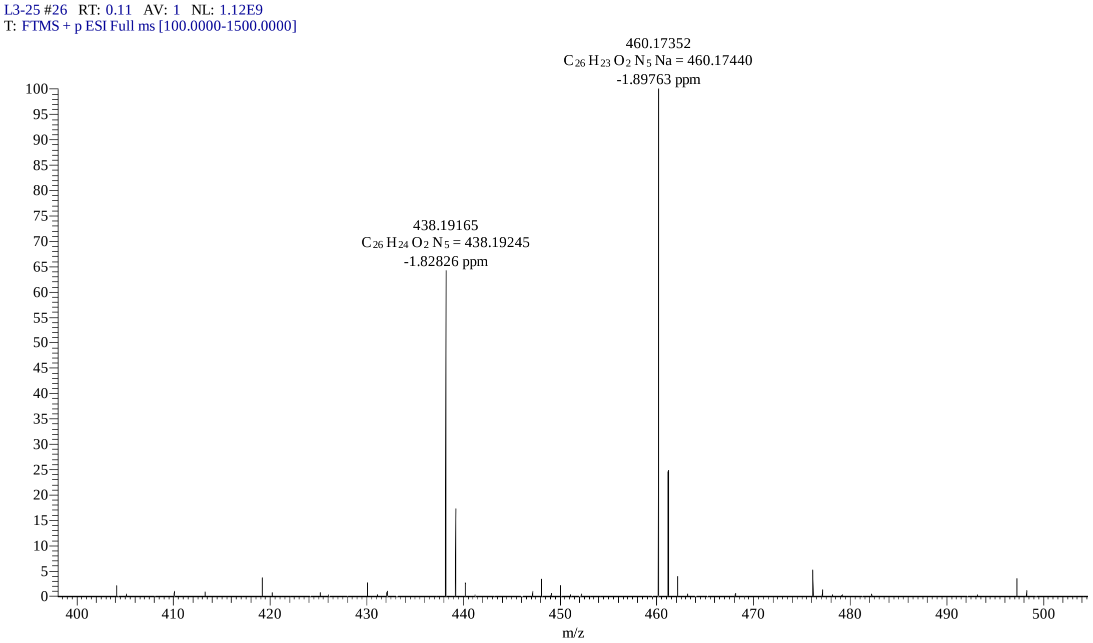
L3-25 #26 RT: 0.11 AV: 1 NL: 1.12E9
T: FTMS + p ESI Full ms [100.0000-1500.0000]
460.17352
C26 H23 O2 N5 Na = 460.17440
-1.89763 ppm
438.19165
C26 H24 O2 N5 = 438.19245
-1.82826 ppm
m/z

Spectra of compound **10h** (^{1}H NMR, ^{13}C NMR, HRMS)

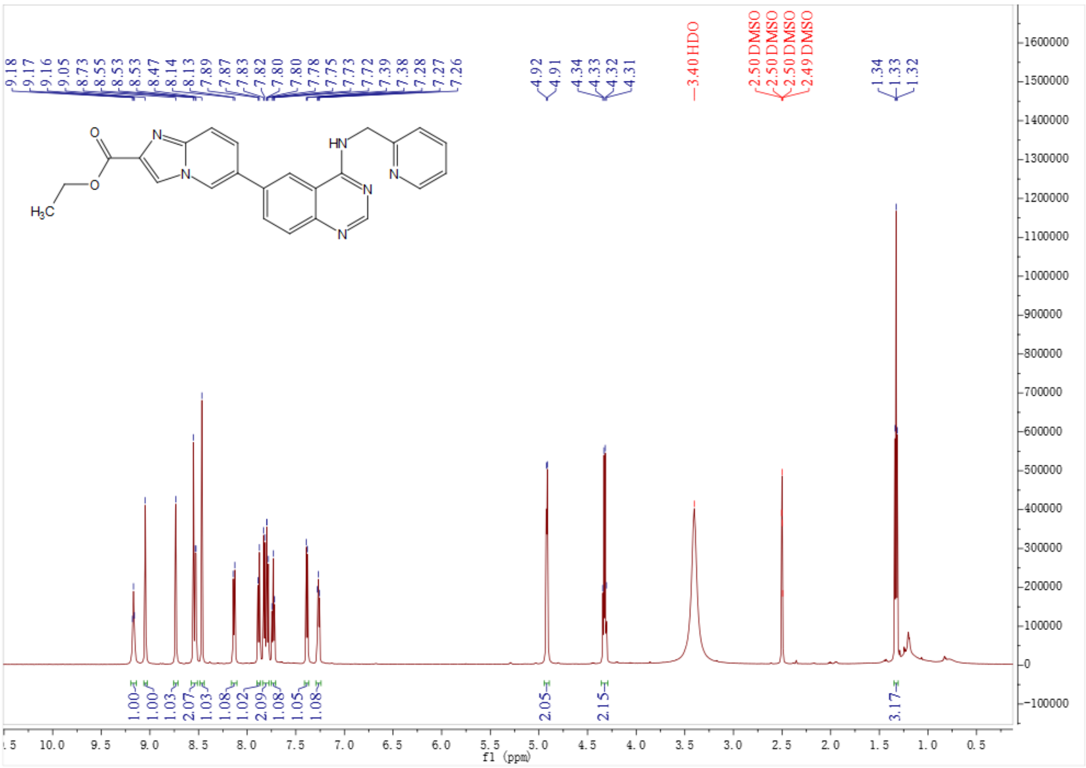

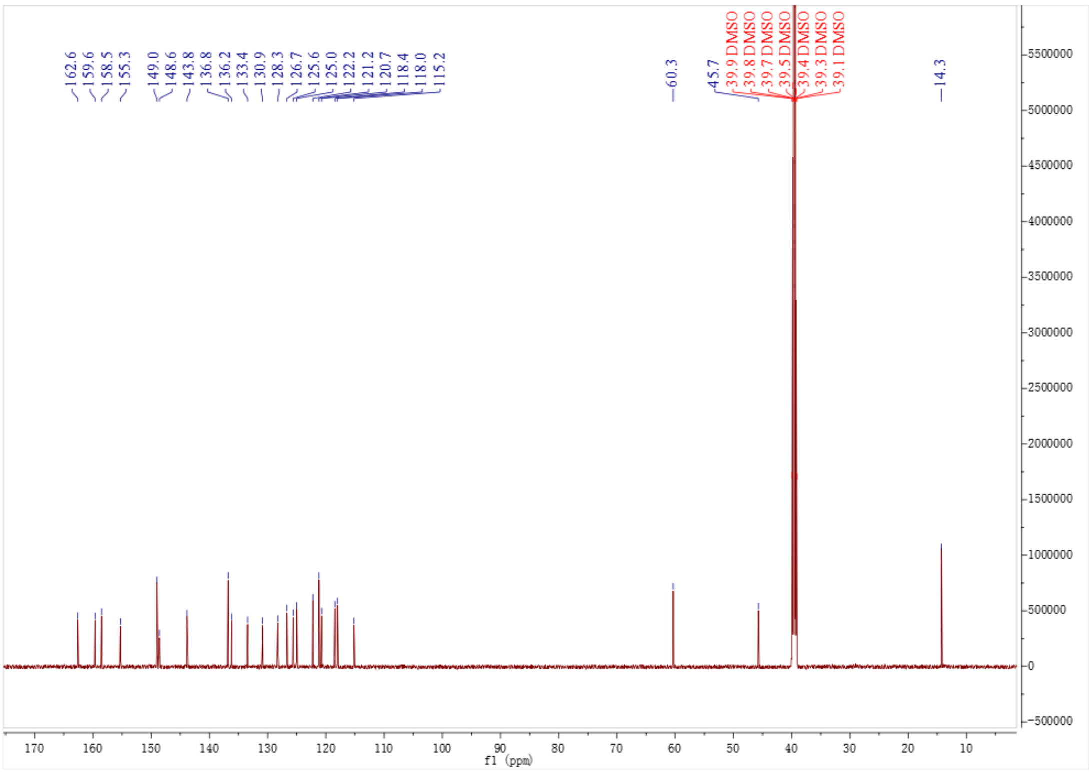

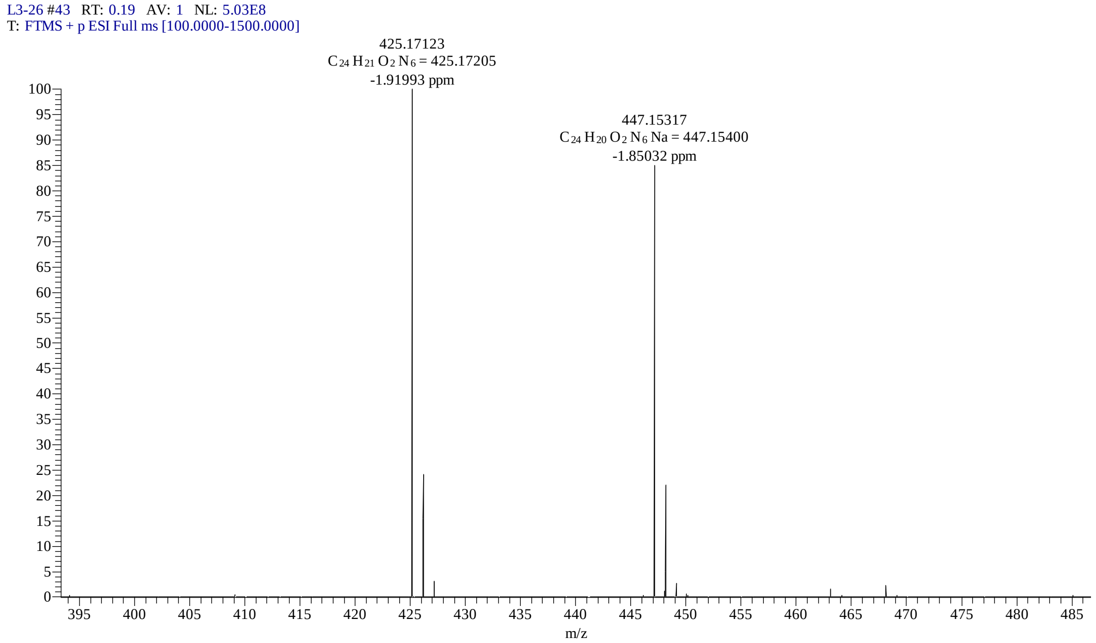

Spectra of compound **10i** (^{1}H NMR, ^{13}C NMR, ^{19}F NMR, HRMS)

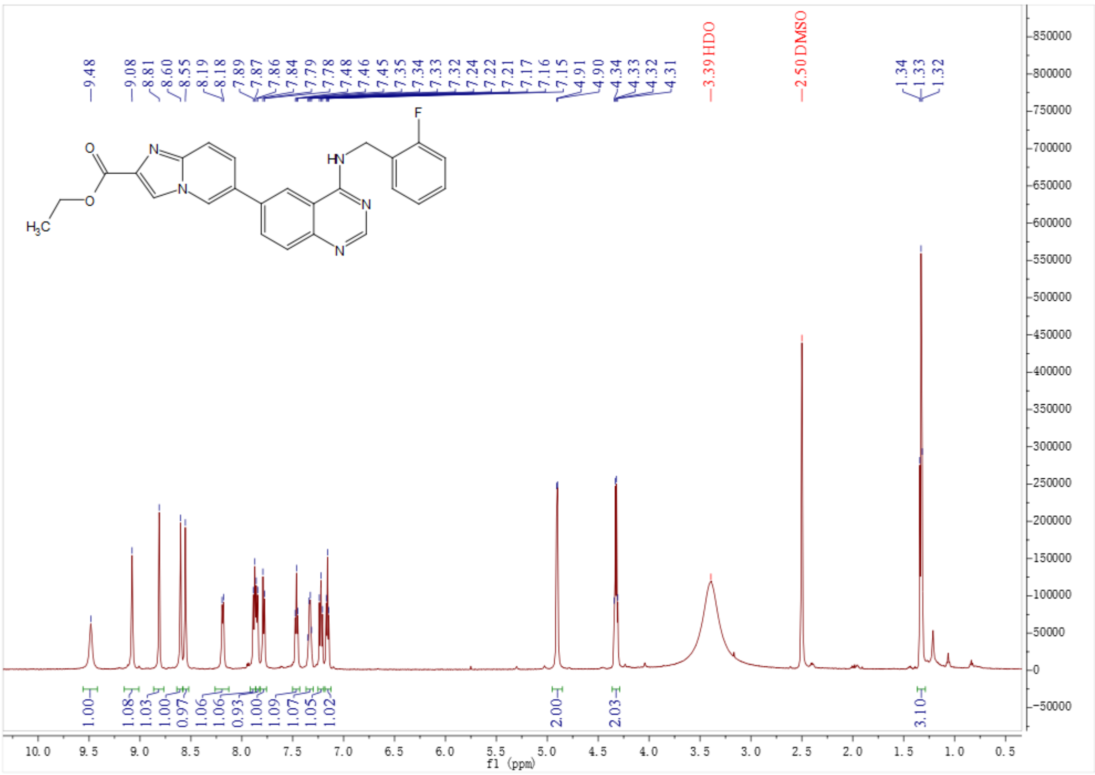

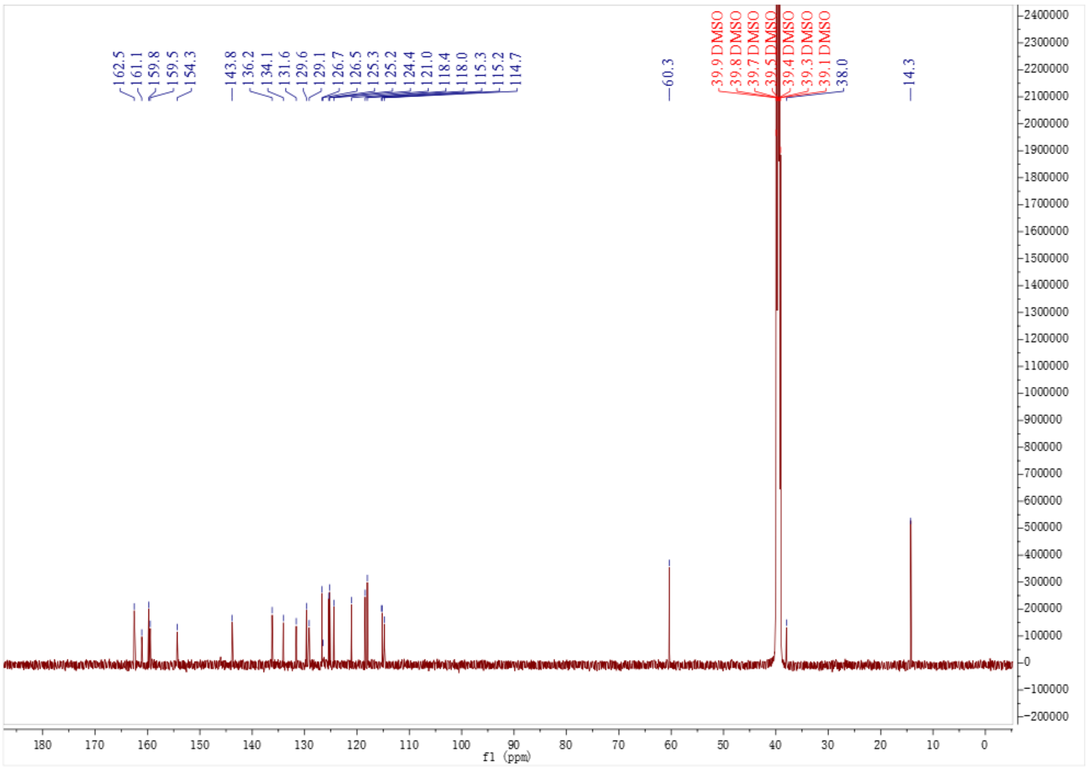
162.5
161.1
159.8
159.5
154.3
143.8
136.2
134.1
131.6
129.6
129.1
126.7
126.5
125.3
125.2
124.4
121.0
118.4
118.0
115.3
115.2
114.7
60.3
39.9 DMSO
39.8 DMSO
39.7 DMSO
39.5 DMSO
39.4 DMSO
39.3 DMSO
39.1 DMSO
38.0
14.3
f1 (ppm)

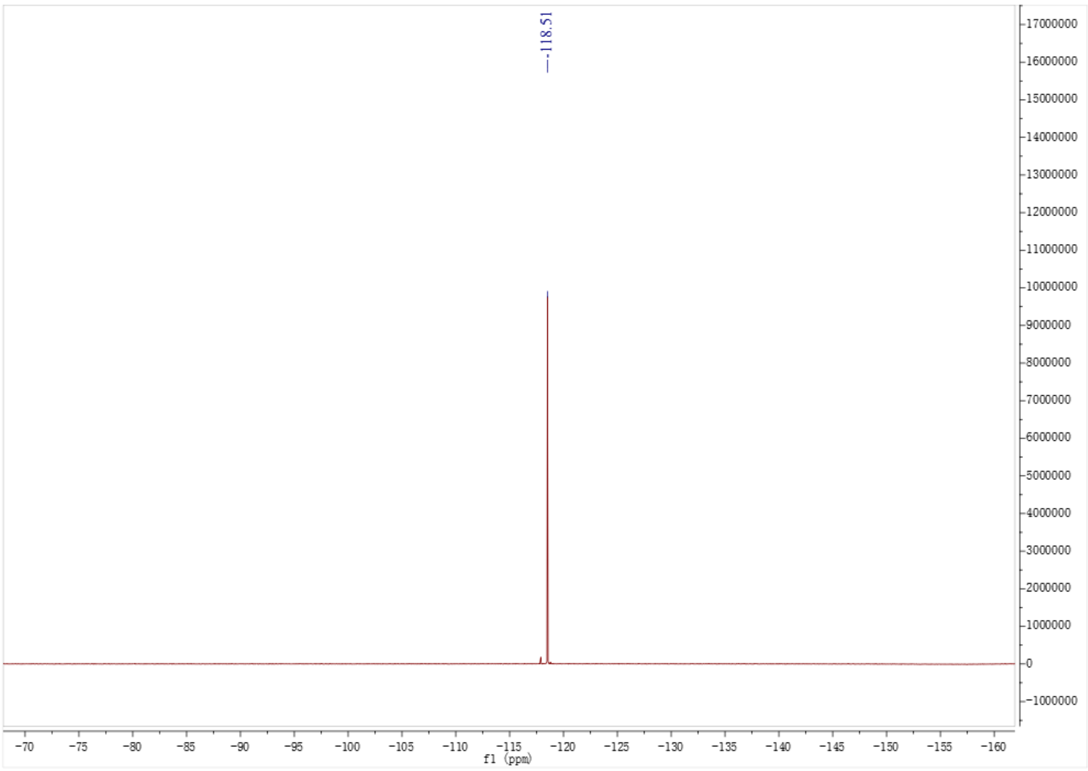
-118.51
f1 (ppm)

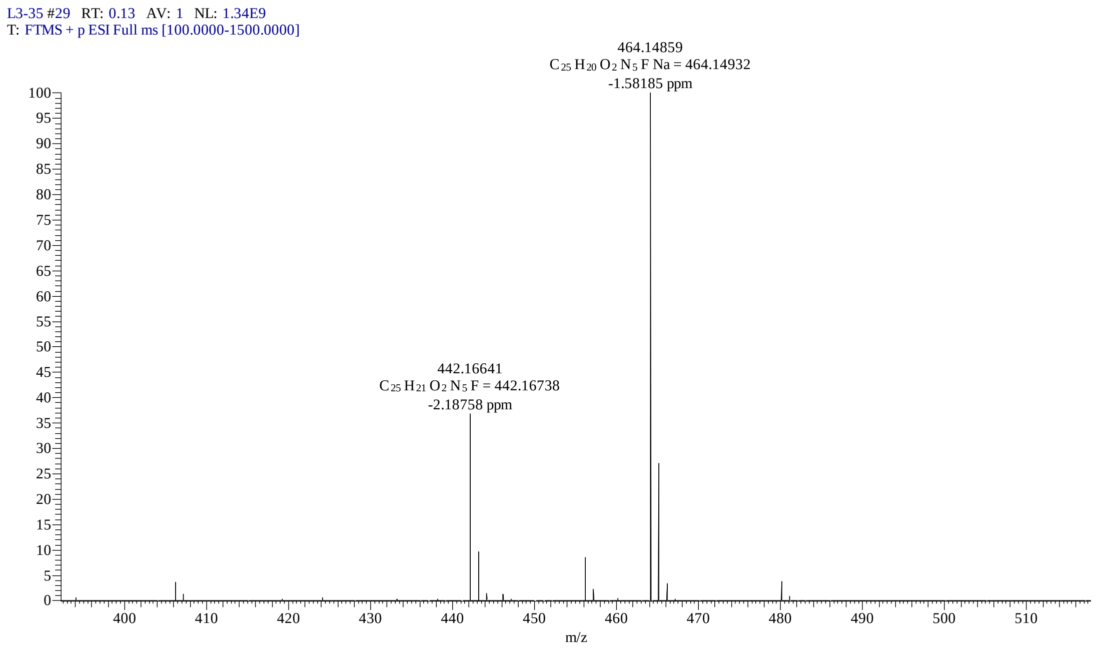

Spectra of compound **10j** (^{1}H NMR, ^{13}C NMR, ^{19}F NMR, HRMS)

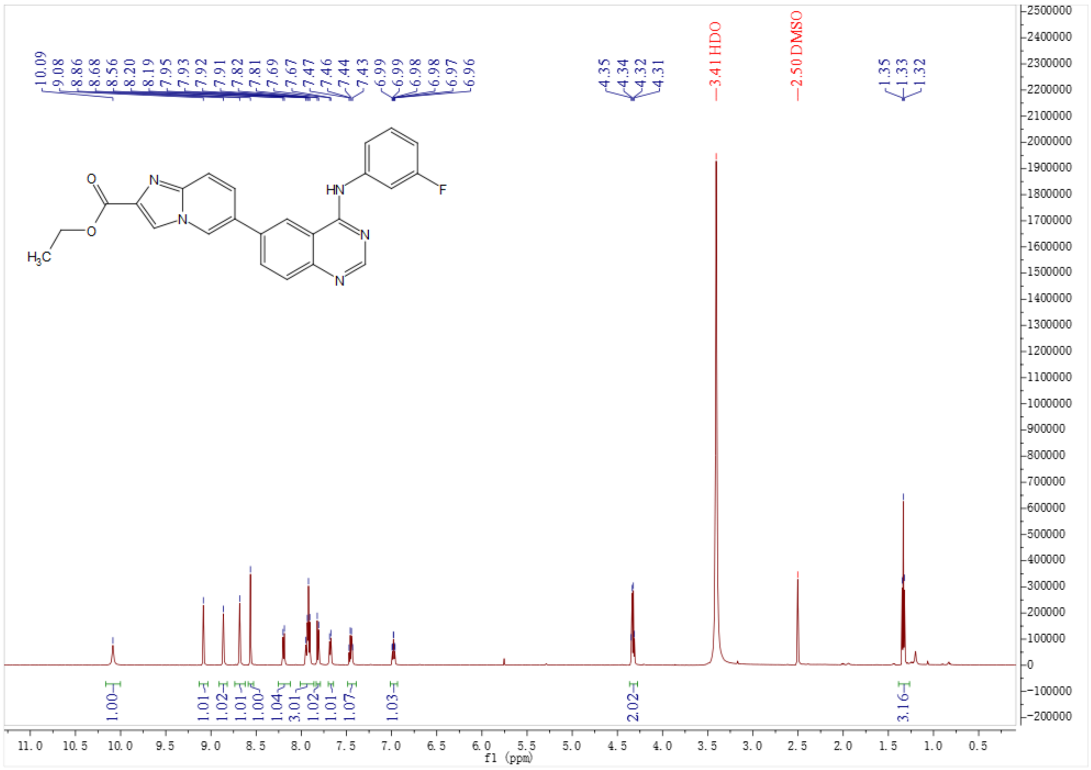

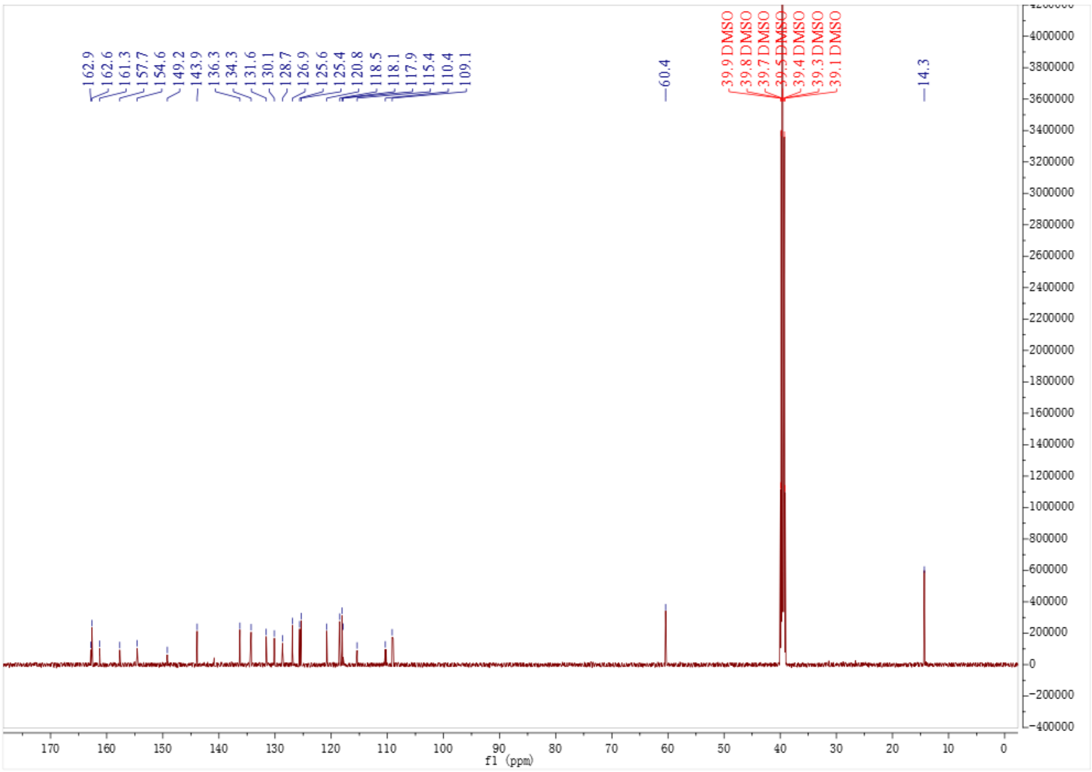
162.9
162.6
161.3
157.7
154.6
149.2
143.9
136.3
134.3
131.6
130.1
128.7
126.9
125.6
125.4
120.8
118.5
118.1
117.9
115.4
110.4
109.1
60.4
39.9 DMSO
39.8 DMSO
39.7 DMSO
39.5 DMSO
39.4 DMSO
39.3 DMSO
39.1 DMSO
14.3
f1 (ppm)

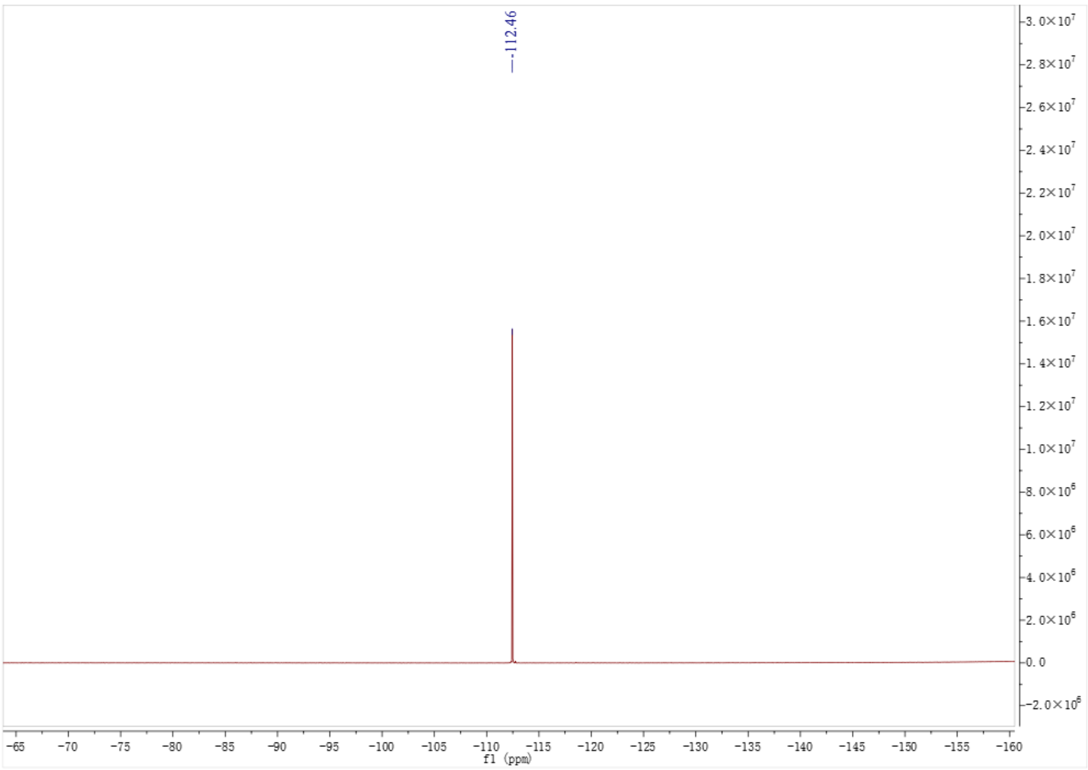
-112.46
f1 (ppm)

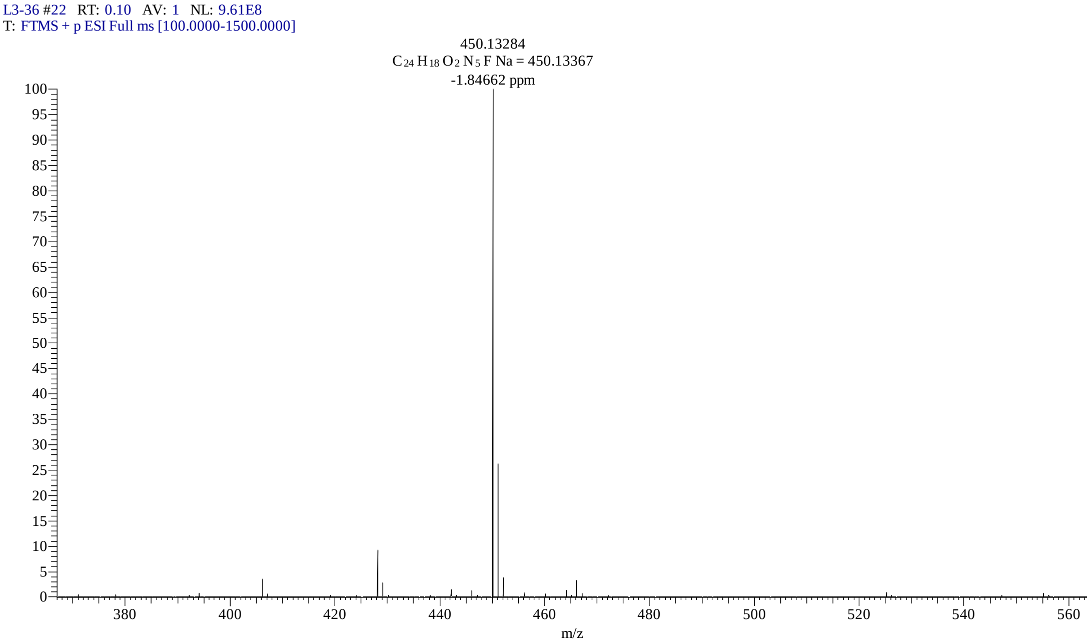

Spectra of compound **10k** (^{1}H NMR, ^{13}C NMR, HRMS)

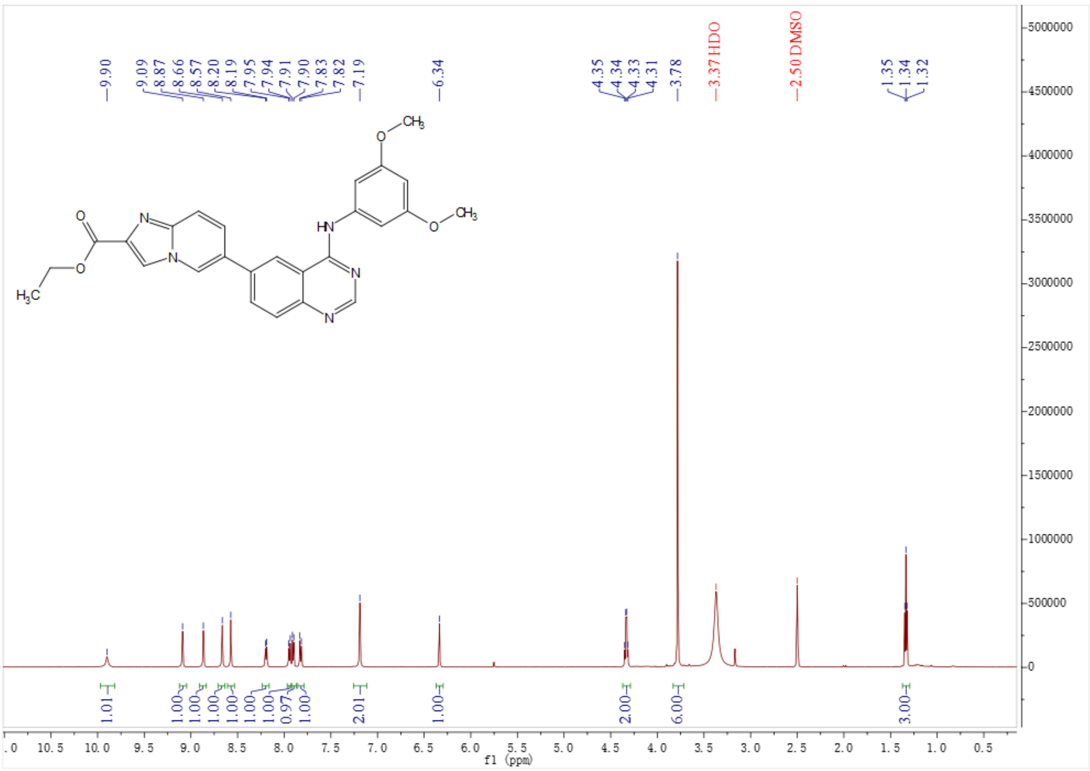

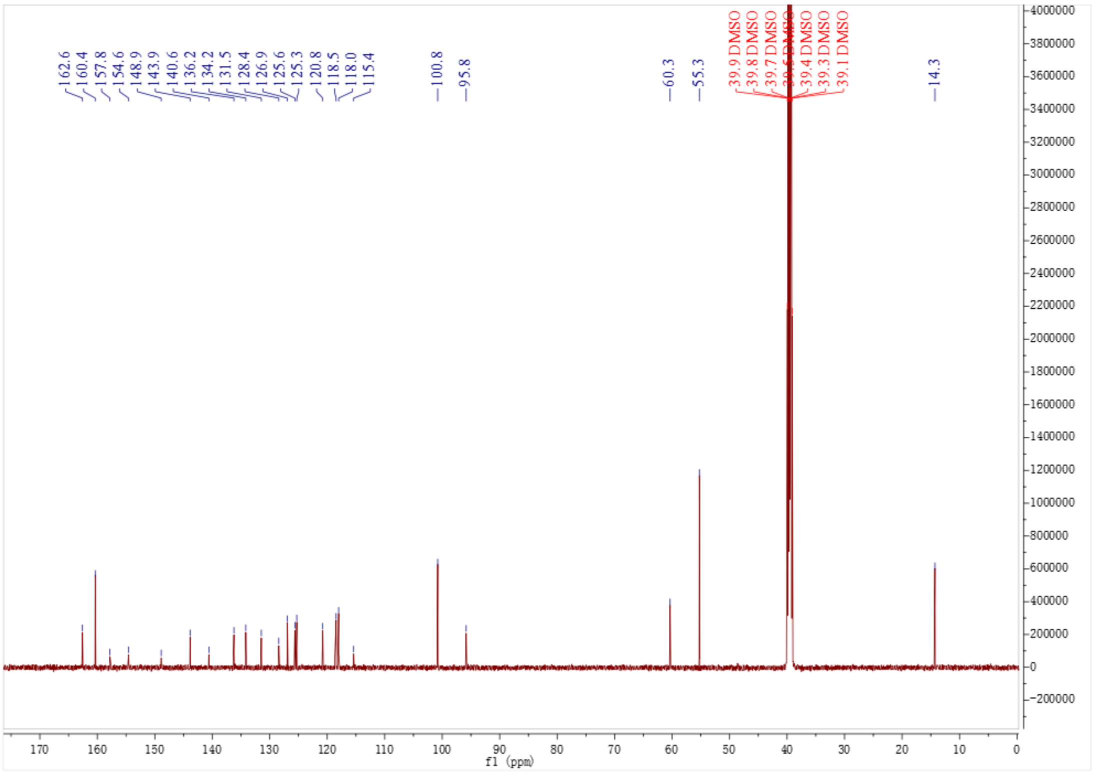
162.6
160.4
157.8
154.6
148.9
143.9
140.6
136.2
134.2
131.5
128.4
126.9
125.6
125.3
120.8
118.5
118.0
115.4
100.8
95.8
60.3
55.3
39.9 DMSO
39.8 DMSO
39.7 DMSO
39.4 DMSO
39.3 DMSO
39.1 DMSO
14.3
f1 (ppm)

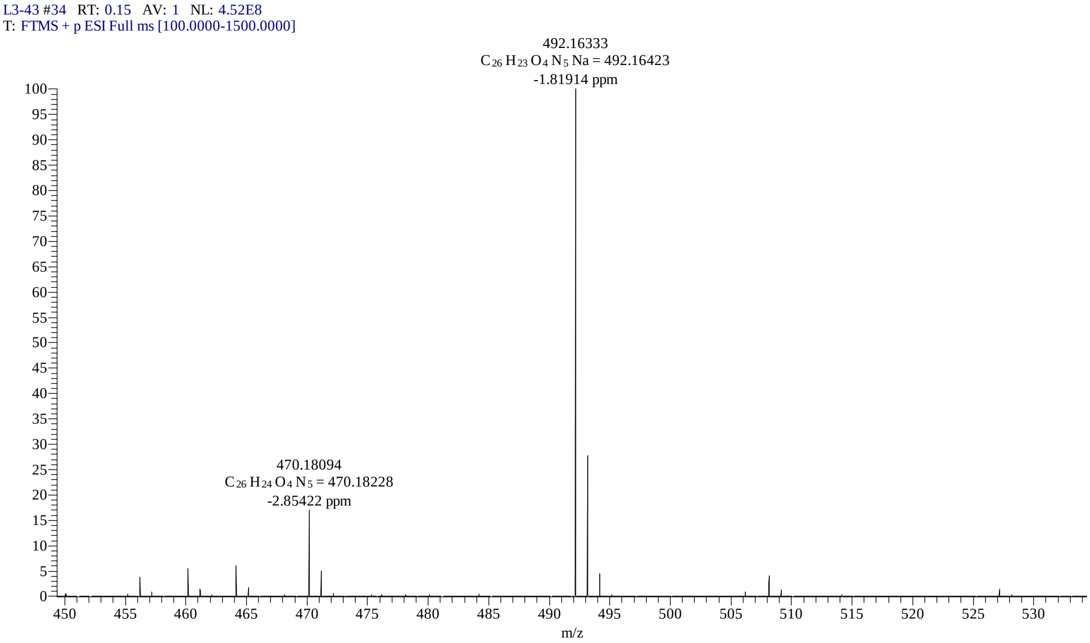
L3-43 #34 RT: 0.15 AV: 1 NL: 4.52E8
T: FTMS + p ESI Full ms [100.0000-1500.0000]
492.16333
C26 H23 O4 N5 Na = 492.16423
-1.81914 ppm
470.18094
C26 H24 O4 N5 = 470.18228
-2.85422 ppm
m/z

Spectra of compound **10l** (^{1}H NMR, ^{13}C NMR, ^{19}F NMR, HRMS)

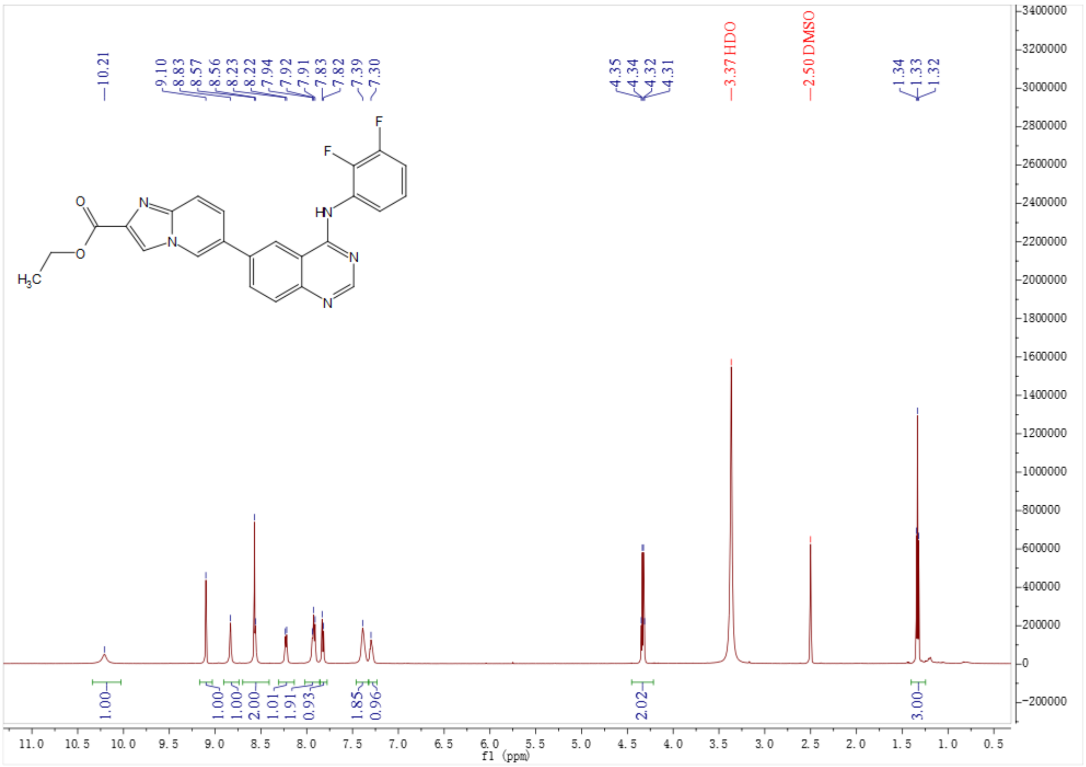

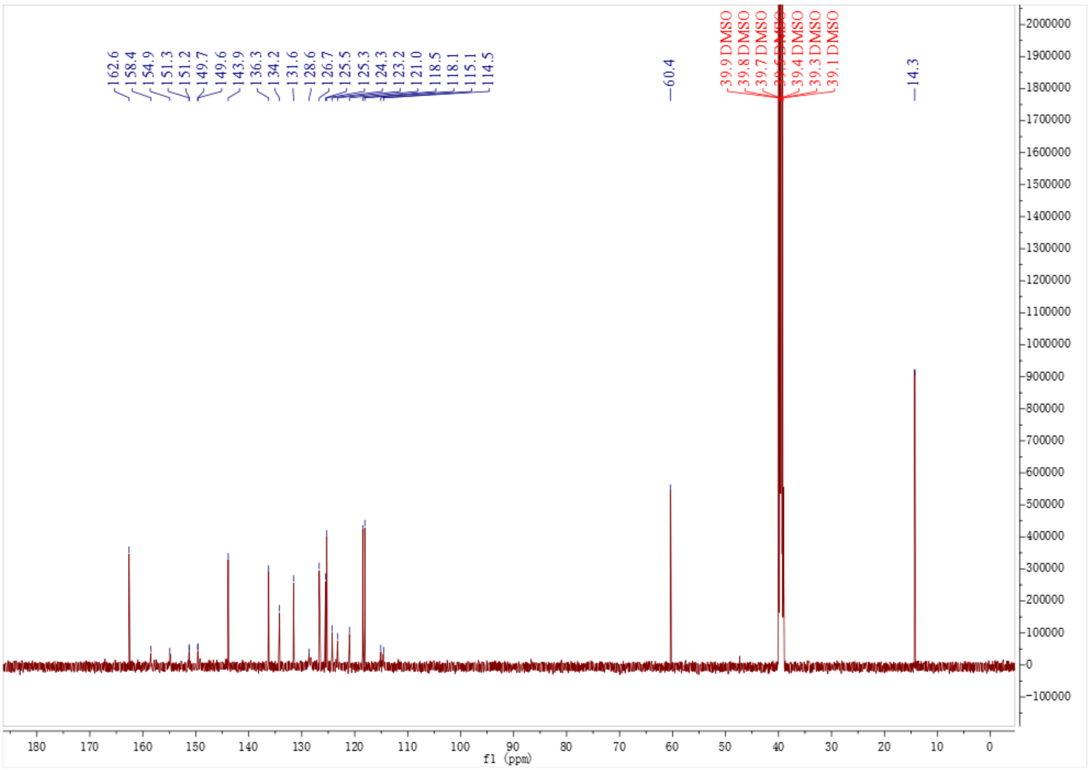

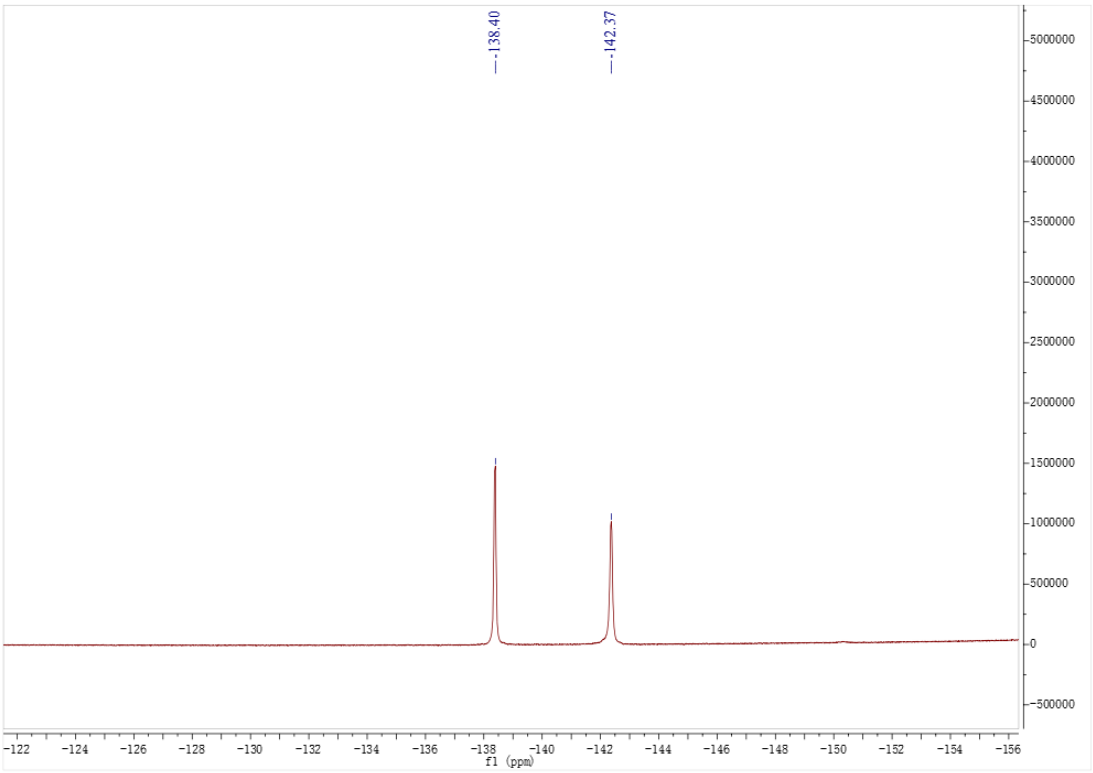

-138.40
-142.37
f1 (ppm)

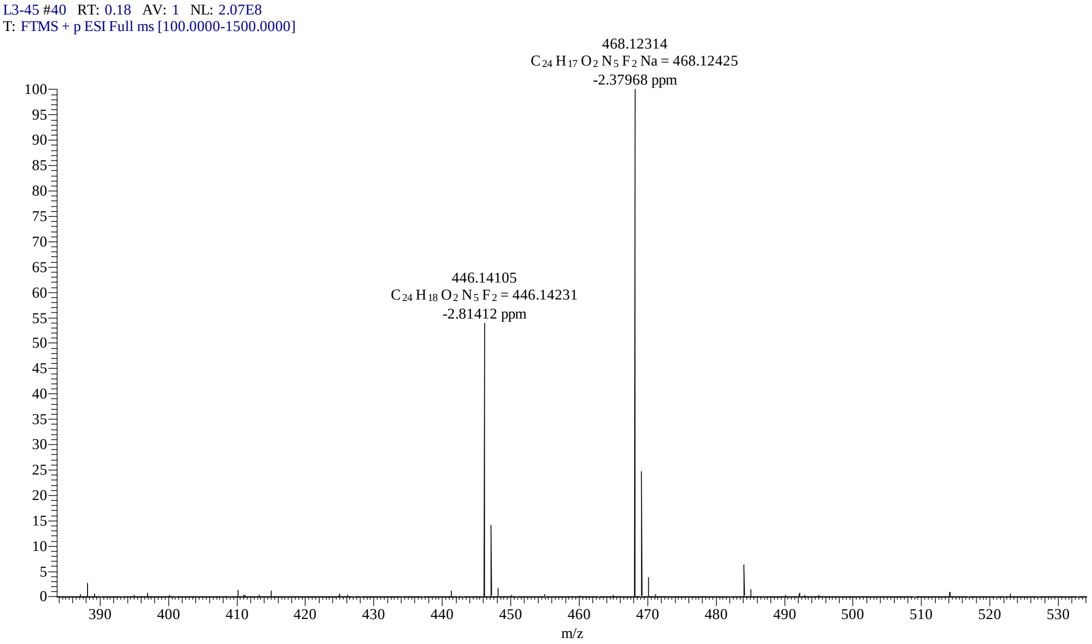

L3-45 #40 RT: 0.18 AV: 1 NL: 2.07E8
T: FTMS + p ESI Full ms [100.0000-1500.0000]
468.12314
C24 H17 O2 N5 F2 Na = 468.12425
-2.37968 ppm
446.14105
C24 H18 O2 N5 F2 = 446.14231
-2.81412 ppm
m/z

Spectra of compound **10m** (^{1}H NMR, ^{13}C NMR, HRMS)

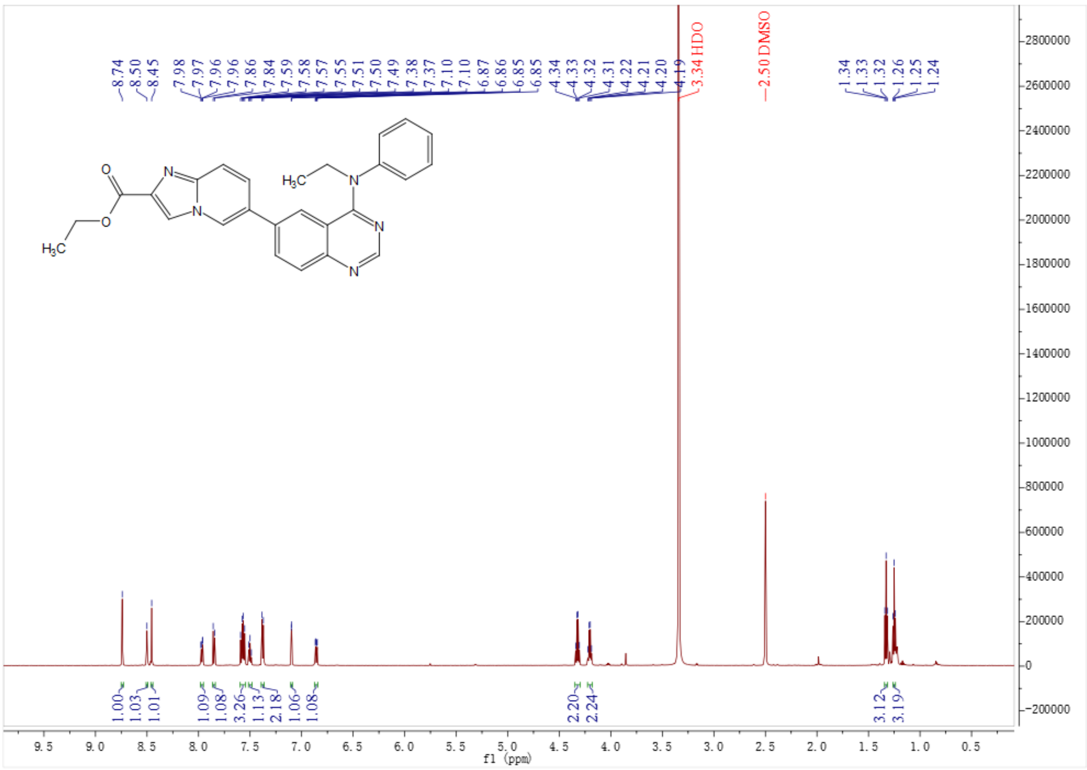

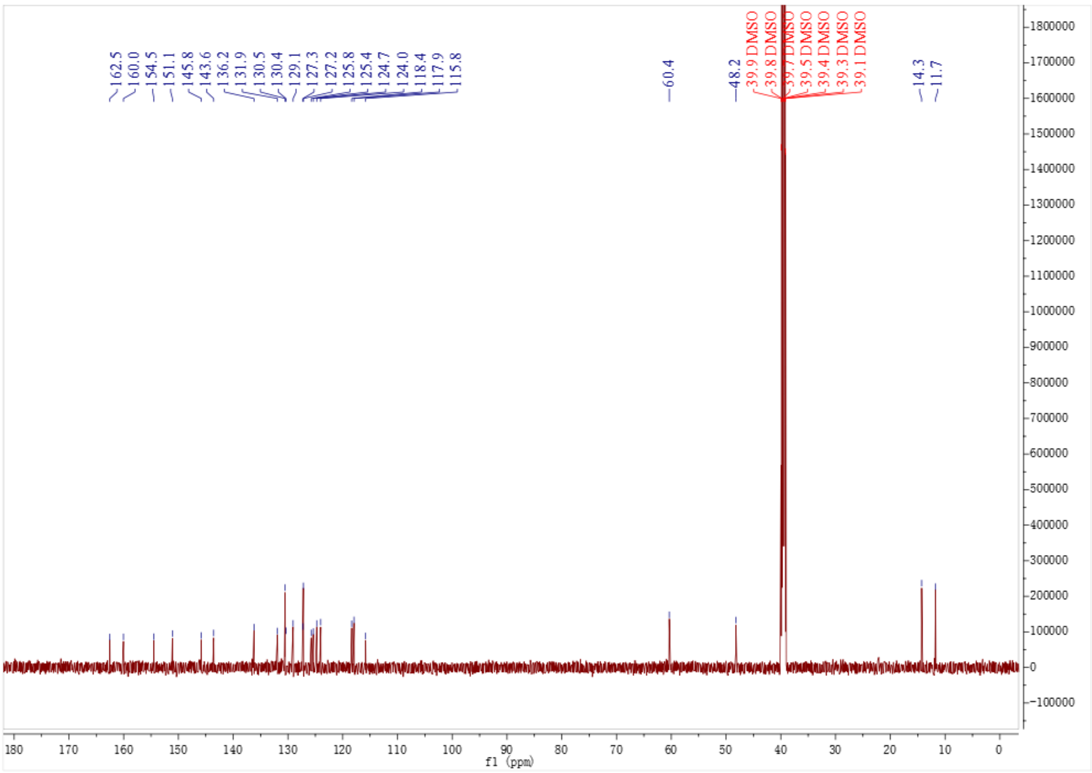

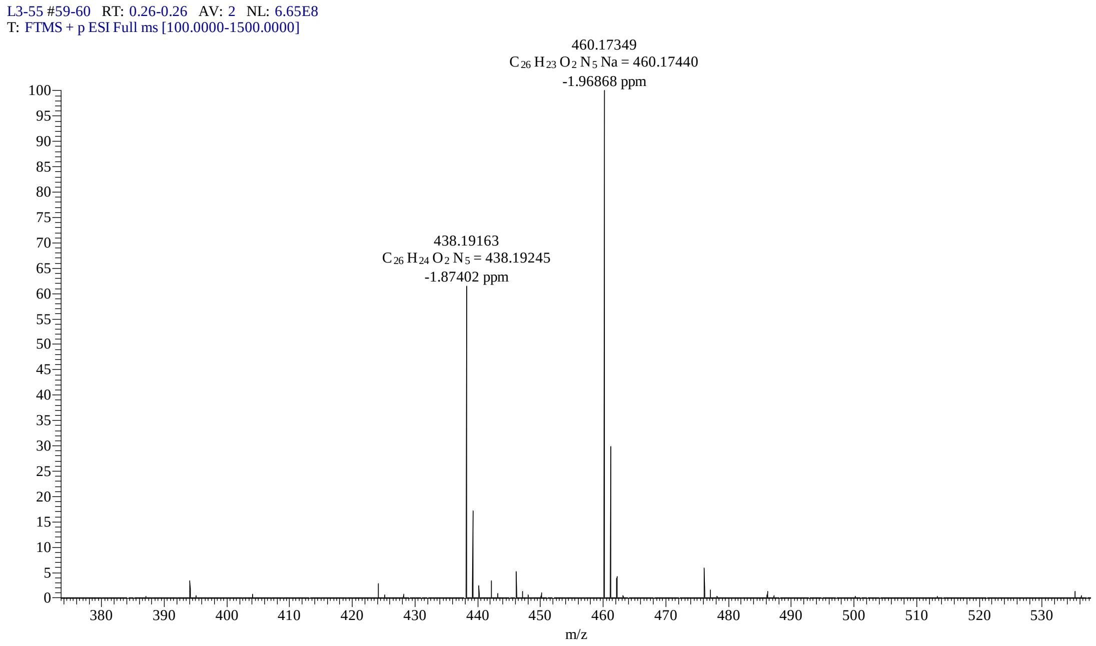

Spectra of compound **10n** (^{1}H NMR, ^{13}C NMR, HRMS)

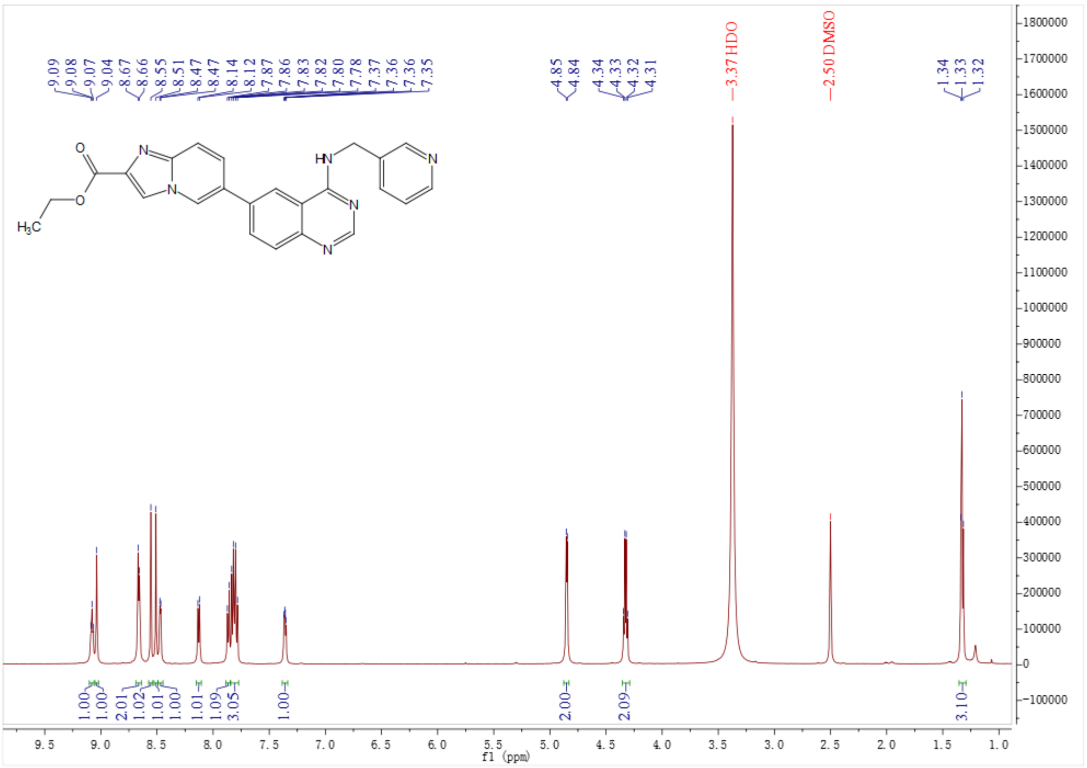

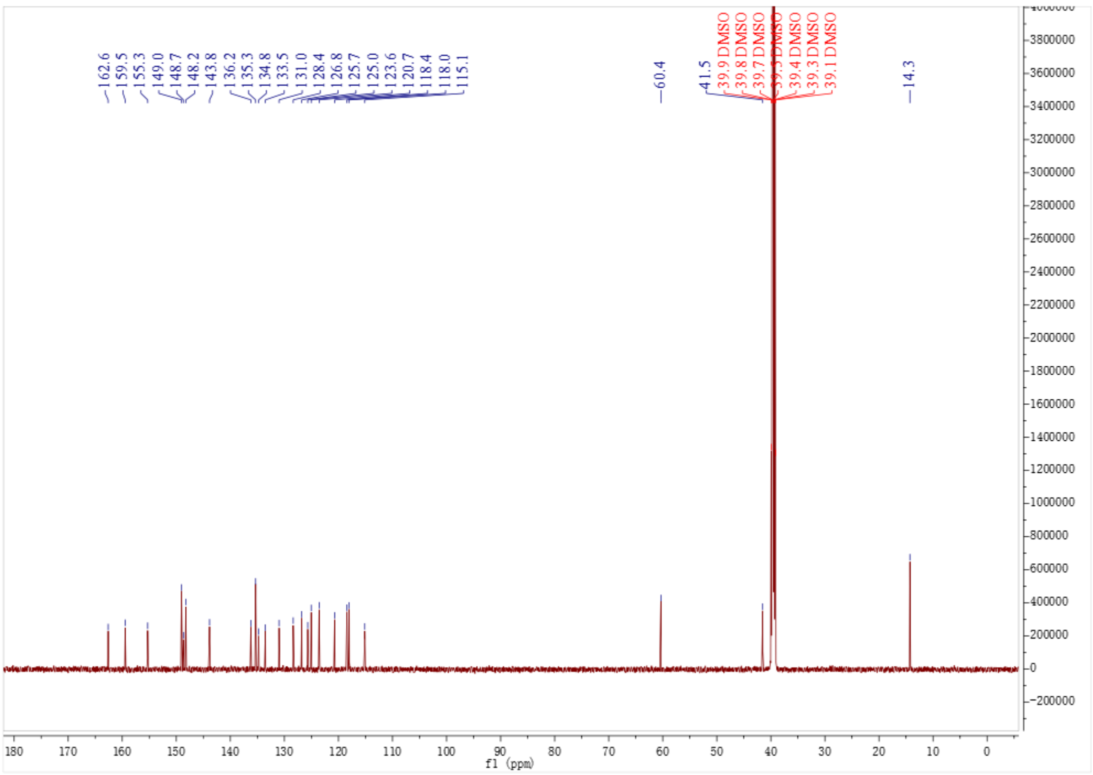
162.6
159.5
155.3
149.0
148.7
148.2
143.8
136.2
135.3
134.8
133.5
131.0
128.4
126.8
125.7
125.0
123.6
120.7
118.4
118.0
115.1
60.4
41.5
39.9 DMSO
39.8 DMSO
39.7 DMSO
39.5 DMSO
39.4 DMSO
39.3 DMSO
39.1 DMSO
14.3
f1 (ppm)

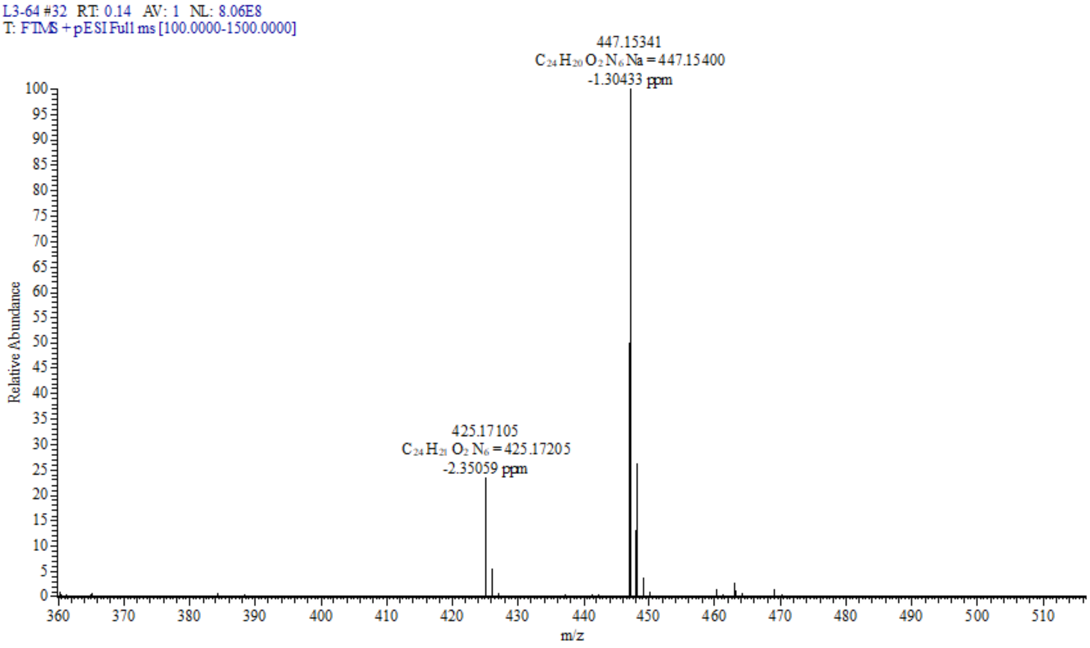
L3-64 #32 RT: 0.14 AV: 1 NL: 8.06E8
T: FTMS + p ESI Full ms [100.0000-1500.0000]
447.15341
C24 H20 O2 N6 Na = 447.15400
-1.30433 ppm
425.17105
C24 H21 O2 N6 = 425.17205
-2.35059 ppm
Relative Abundance
m/z

Spectra of compound **10o** (^{1}H NMR, ^{13}C NMR, HRMS)

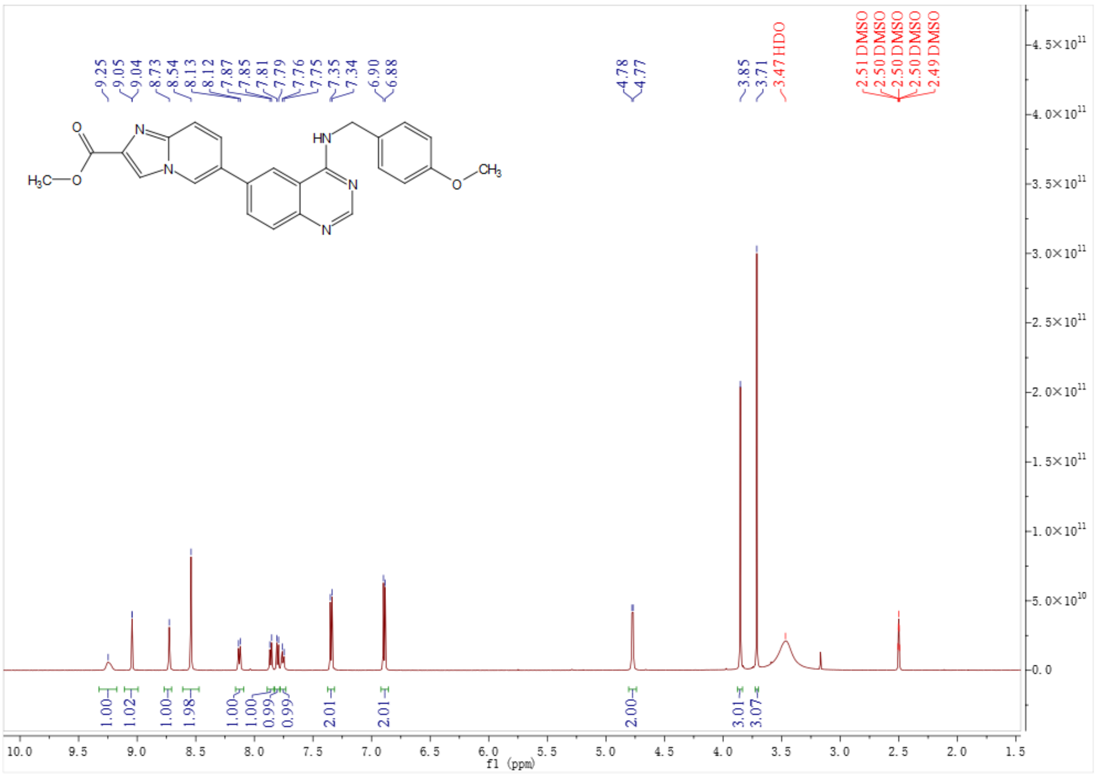

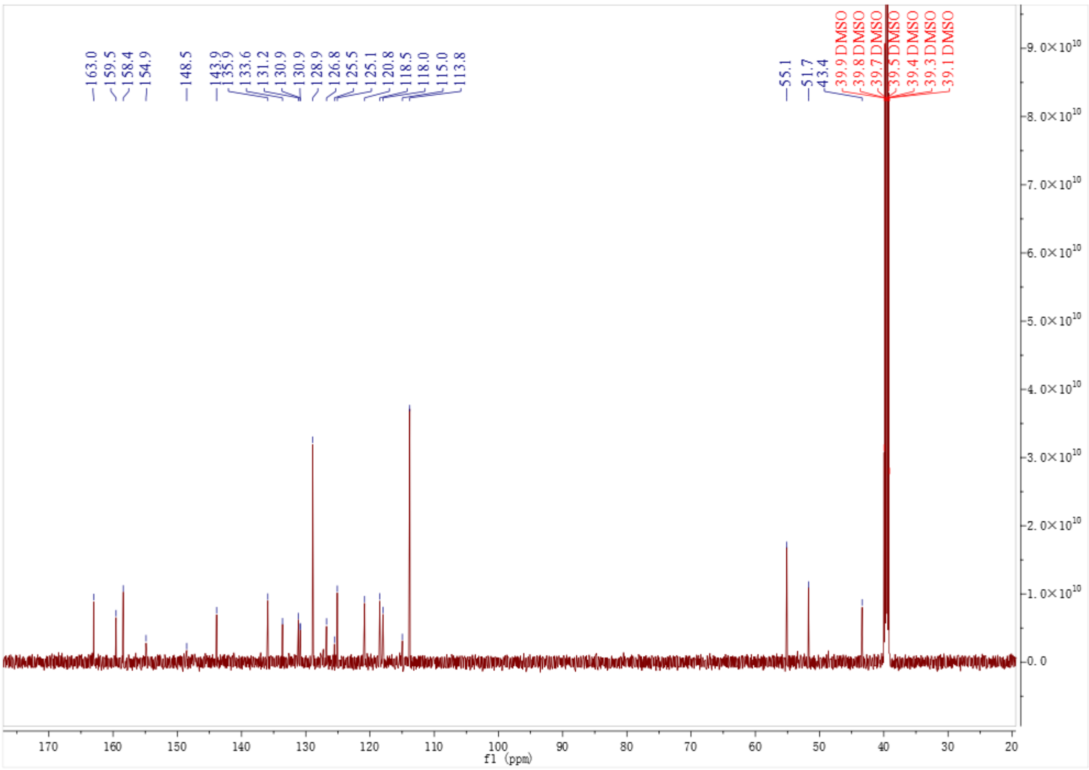

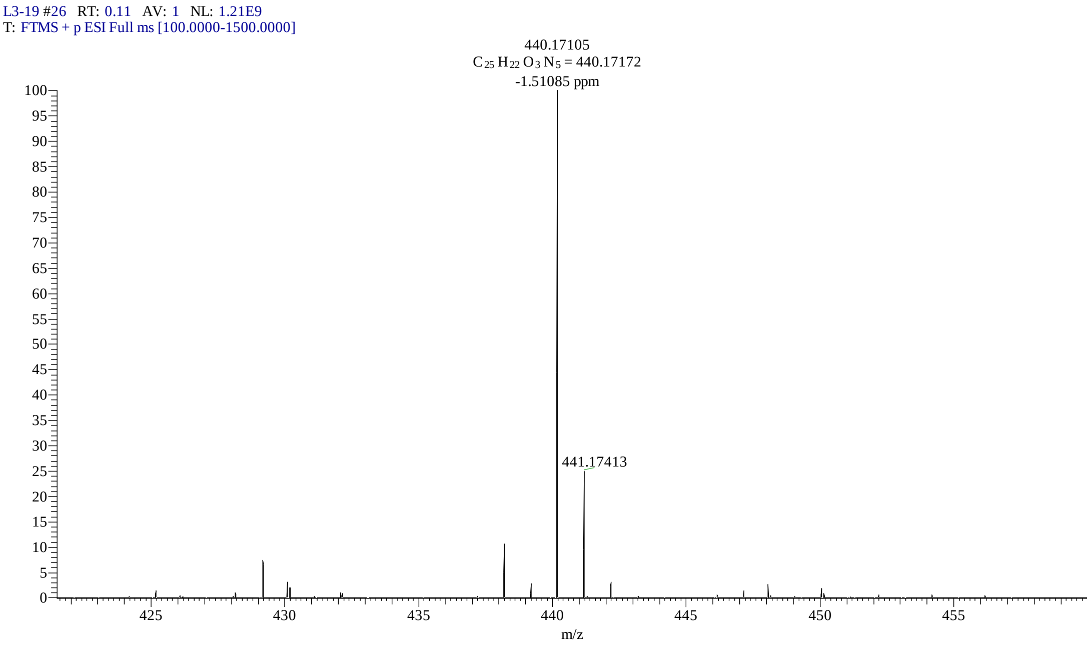

Spectra of compound **10p** (^{1}H NMR, ^{13}C NMR, HRMS)

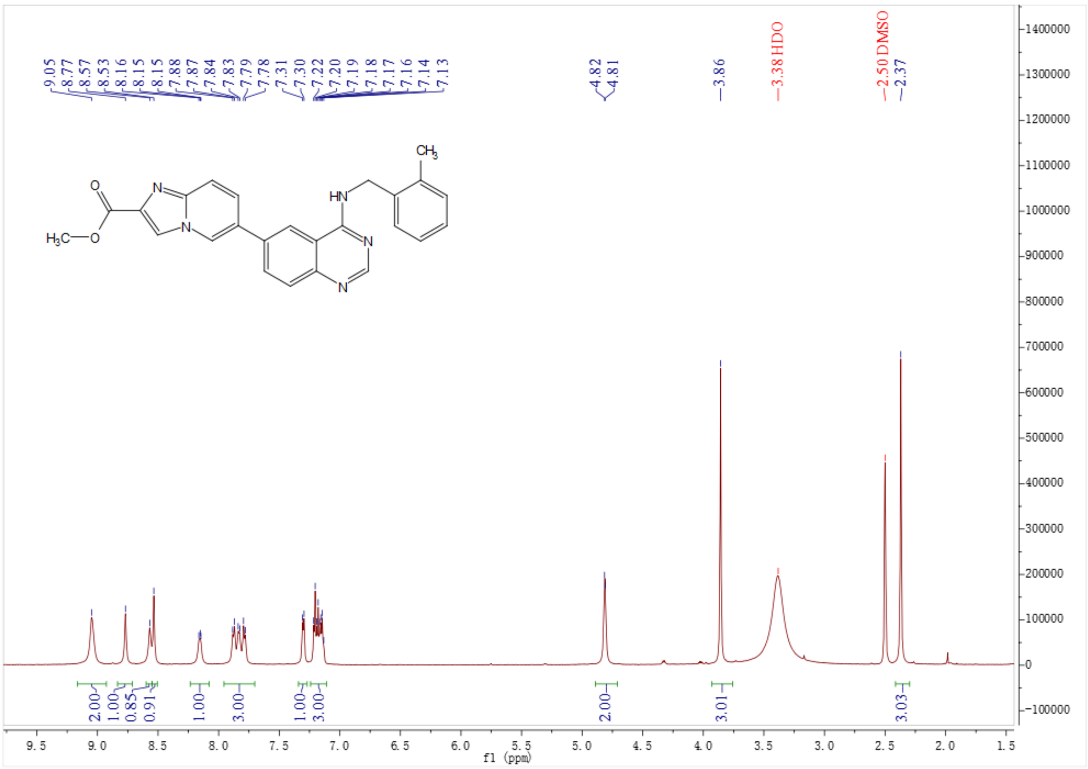

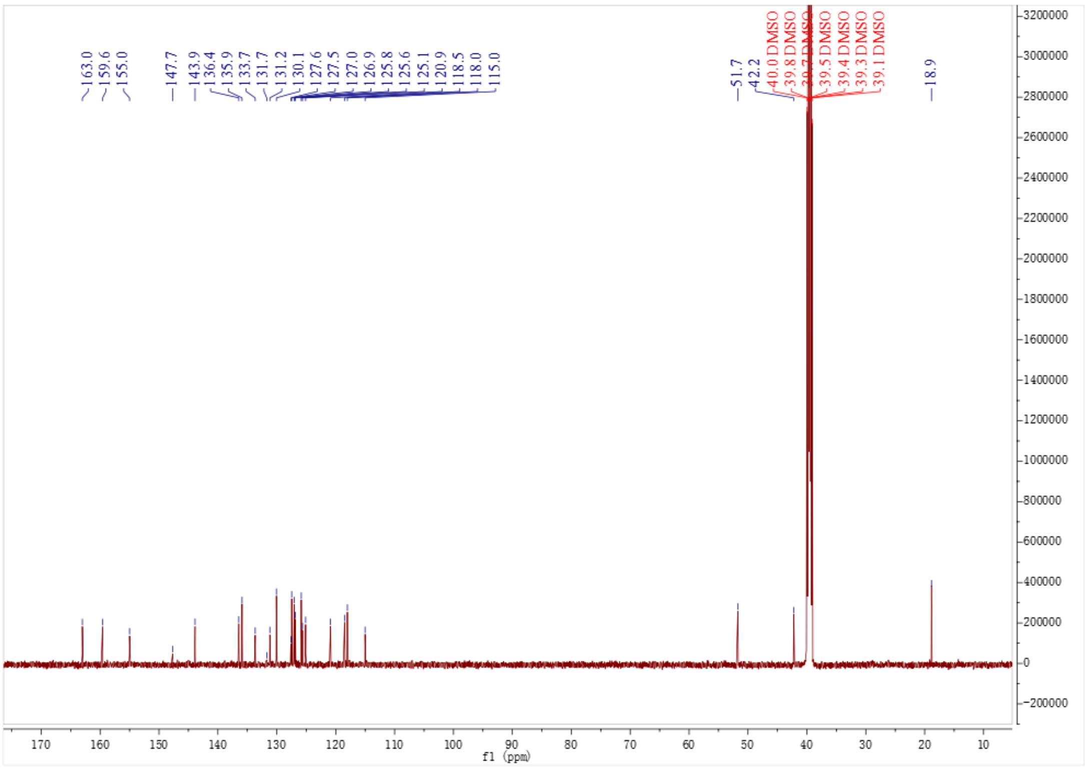

163.0
159.6
155.0
147.7
143.9
136.4
135.9
133.7
131.7
131.2
130.1
127.6
127.5
127.0
126.9
125.8
125.6
125.1
120.9
118.5
118.0
115.0
51.7
42.2
40.0 DMSO
39.8 DMSO
39.7 DMSO
39.5 DMSO
39.4 DMSO
39.3 DMSO
39.1 DMSO
18.9
f1 (ppm)

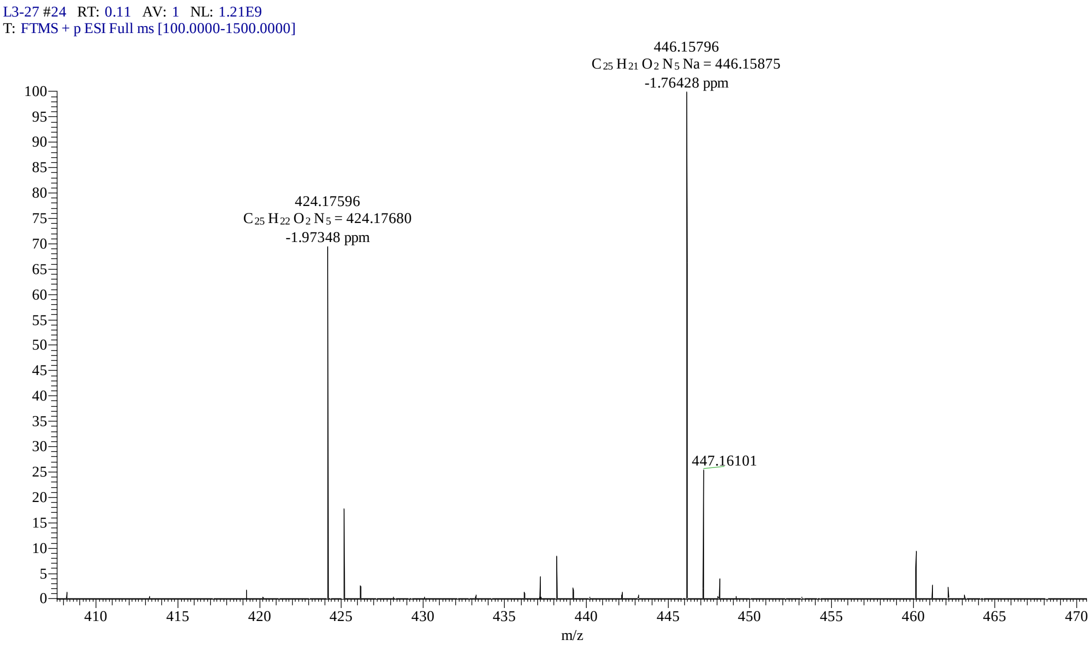

L3-27 #24 RT: 0.11 AV: 1 NL: 1.21E9
T: FTMS + p ESI Full ms [100.0000-1500.0000]
446.15796
C25 H21 O2 N5 Na = 446.15875
-1.76428 ppm
424.17596
C25 H22 O2 N5 = 424.17680
-1.97348 ppm
447.16101
m/z

Spectra of compound **10q** (^{1}H NMR, ^{13}C NMR, HRMS)

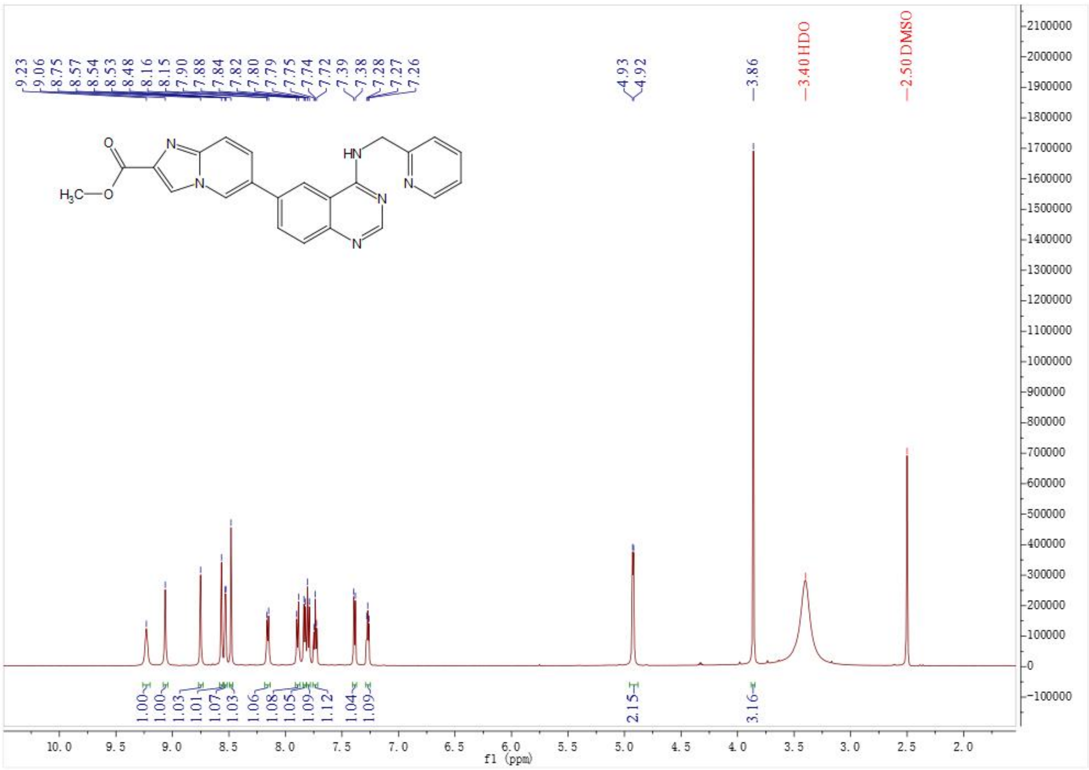

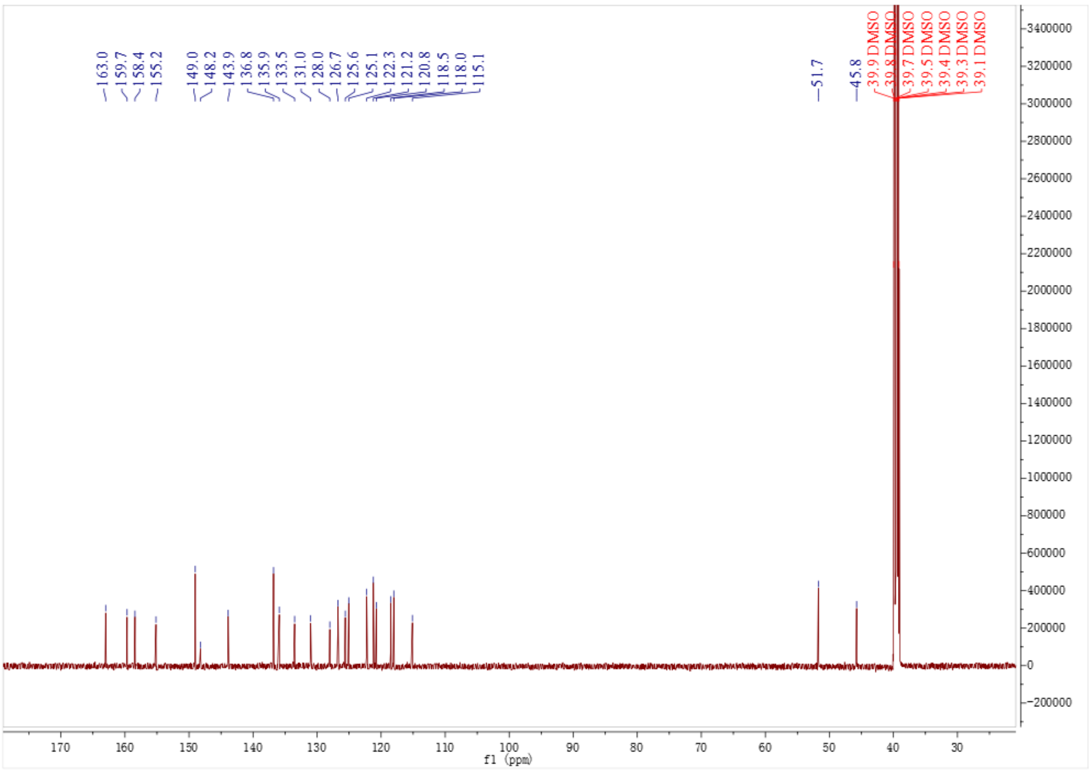

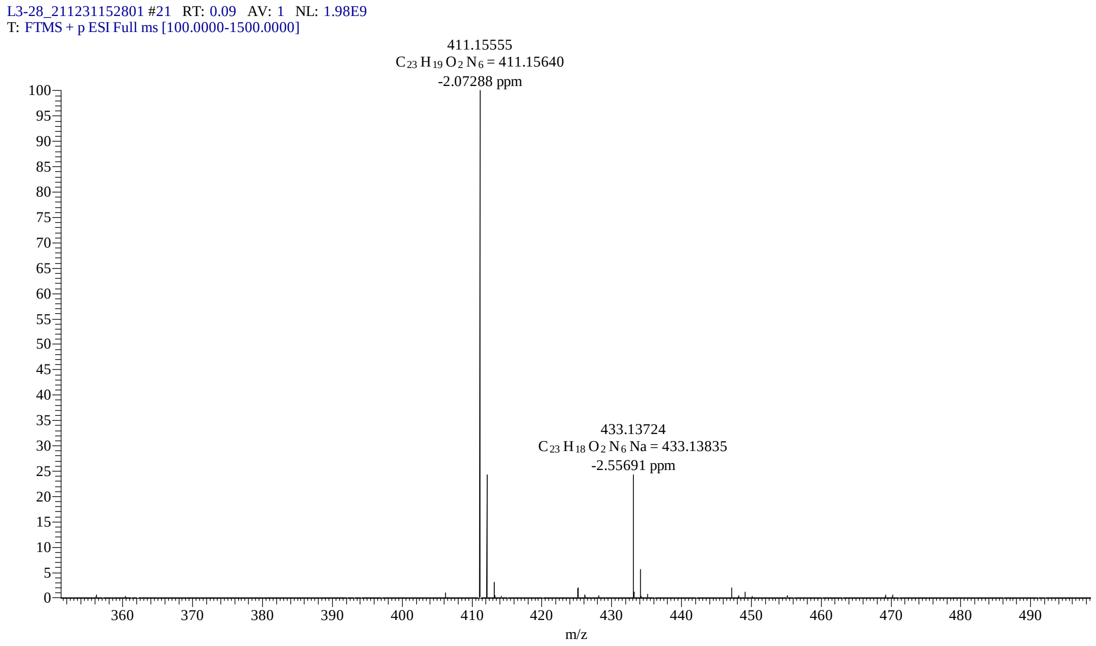

Spectra of compound **10r** (^{1}H NMR, ^{13}C NMR, ^{19}F NMR, HRMS)

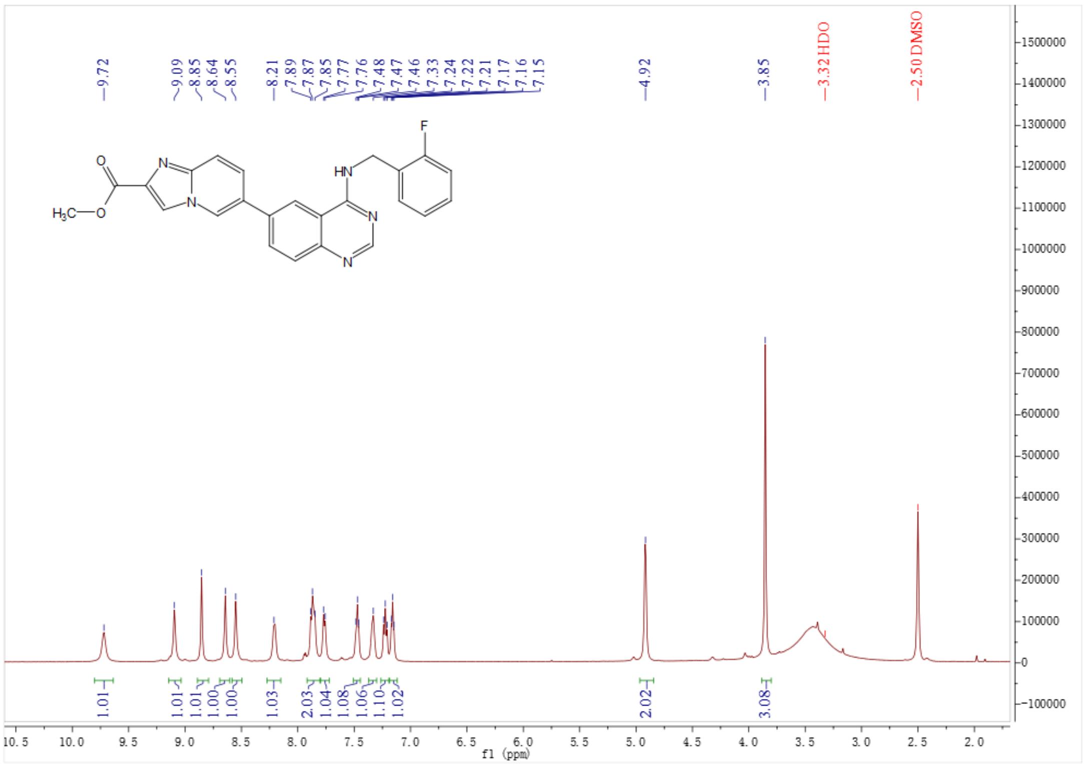

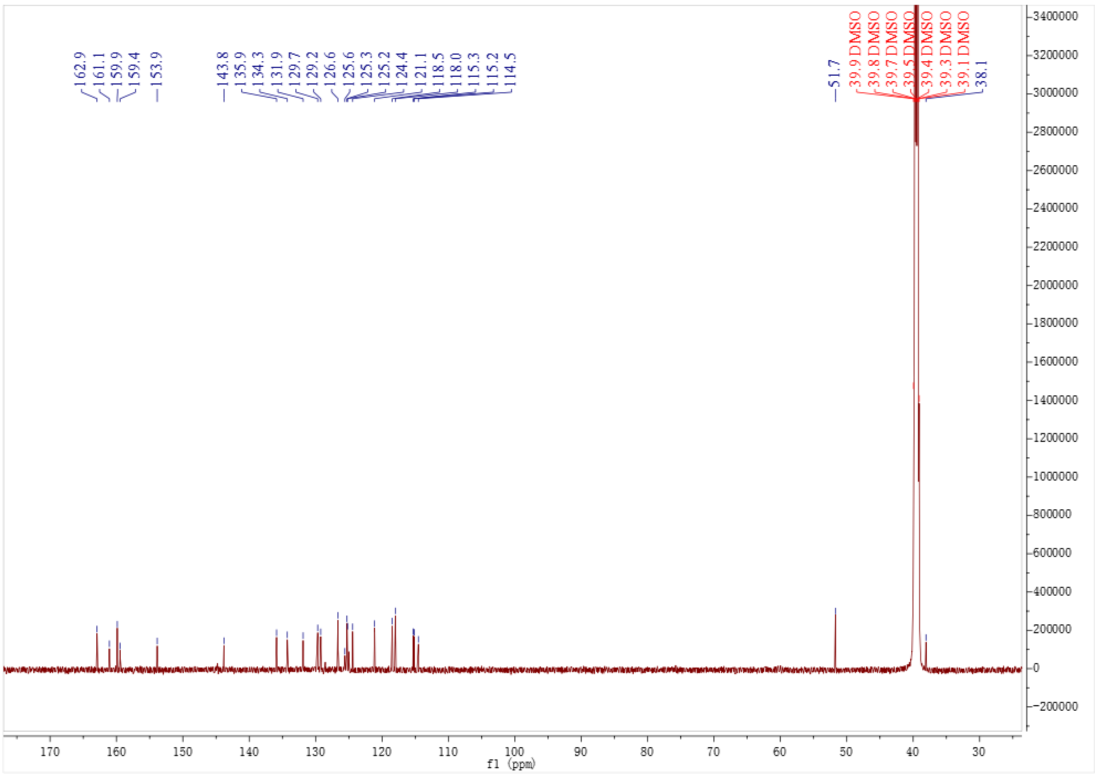
162.9
161.1
159.9
159.4
153.9
143.8
135.9
134.3
131.9
129.7
129.2
126.6
125.6
125.3
125.2
124.4
121.1
118.5
118.0
115.3
115.2
114.5
51.7
39.9 DMSO
39.8 DMSO
39.7 DMSO
39.5 DMSO
39.4 DMSO
39.3 DMSO
39.1 DMSO
38.1
f1 (ppm)

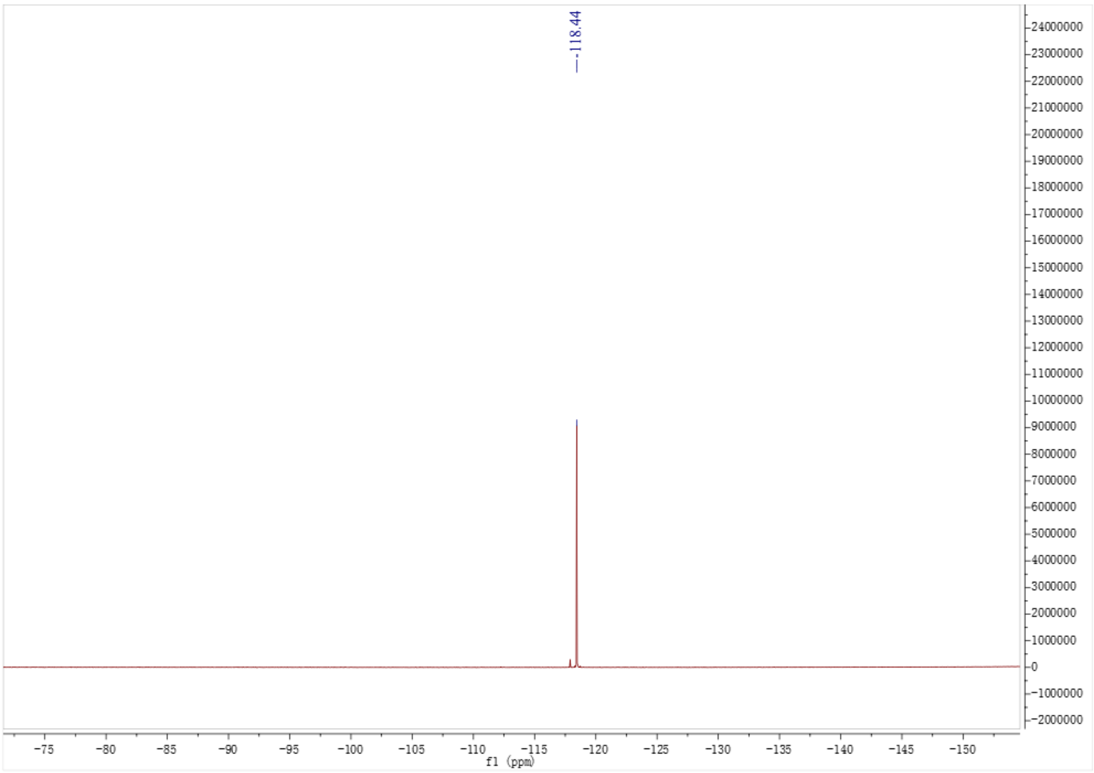
-118.44
f1 (ppm)

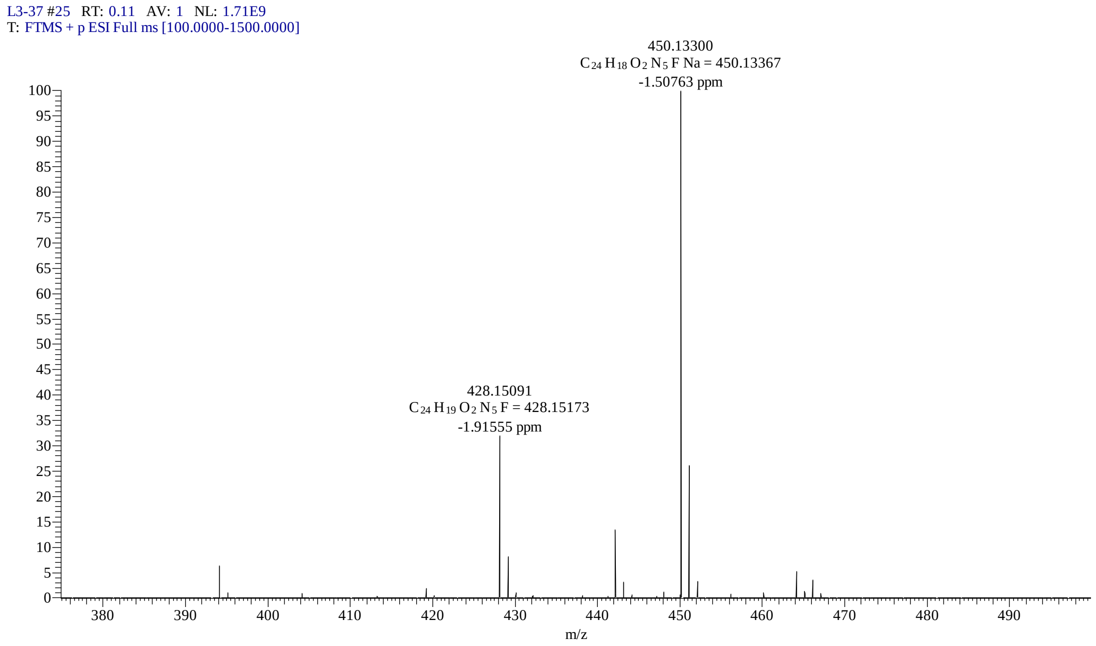

Spectra of compound **10s** (^{1}H NMR, ^{13}C NMR, ^{19}F NMR, HRMS)

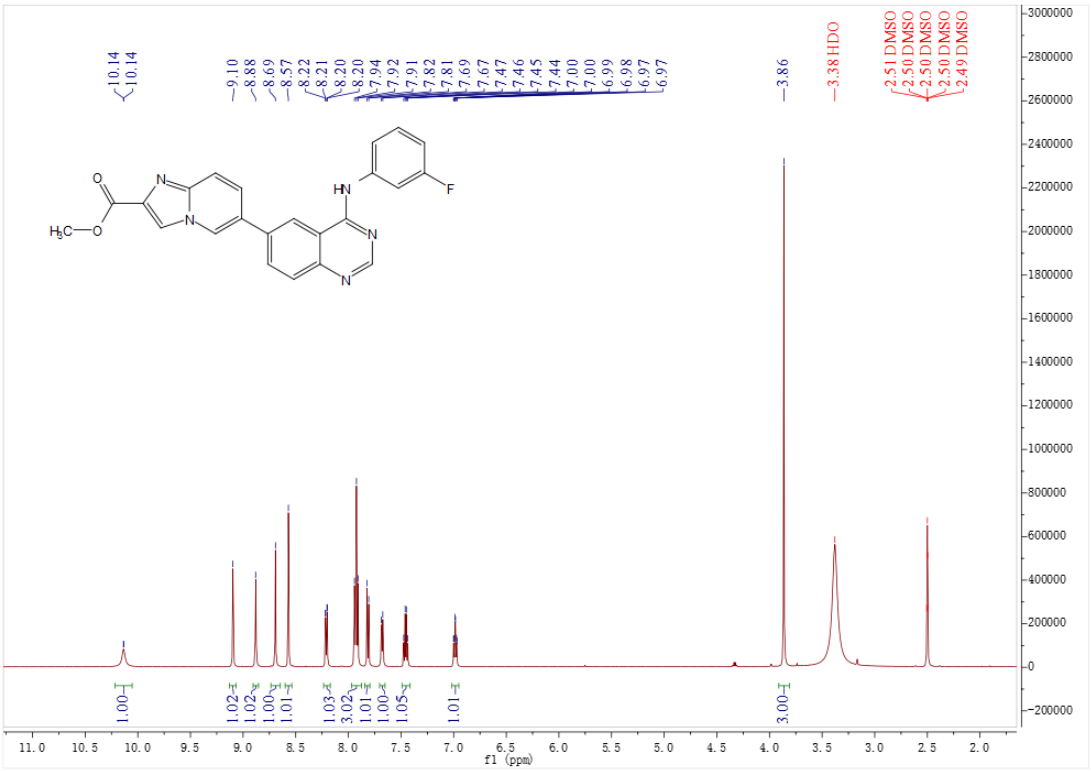

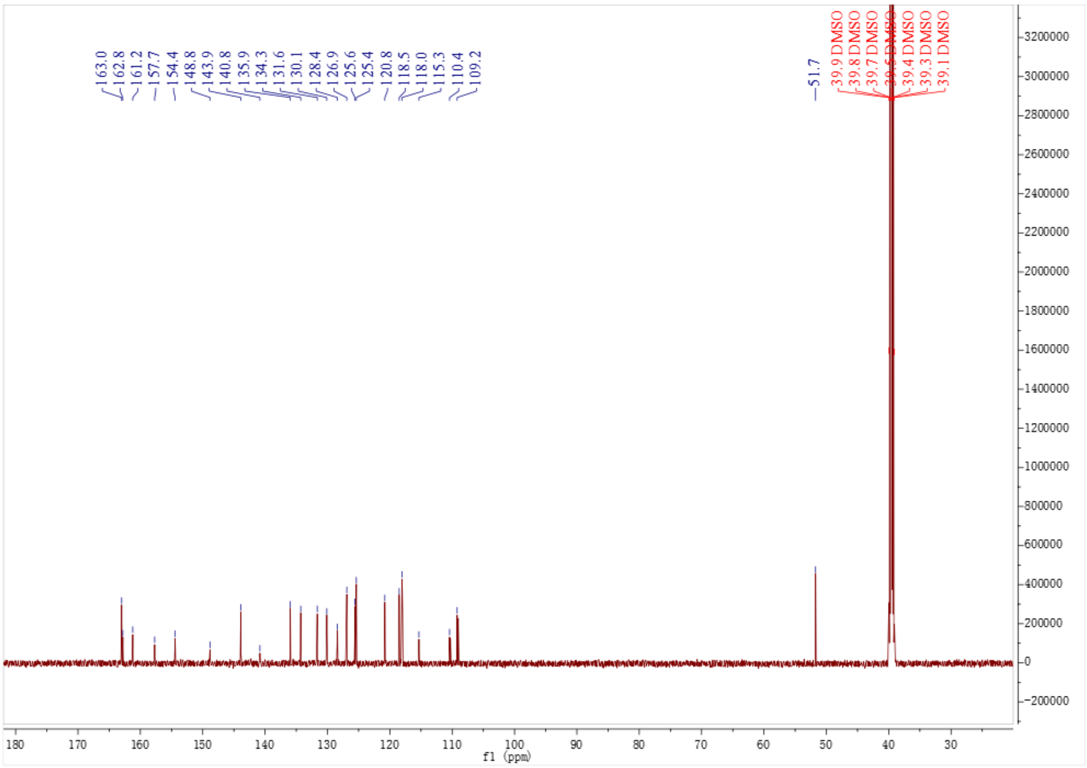

163.0
162.8
161.2
157.7
154.4
148.8
143.9
140.8
135.9
134.3
131.6
130.1
128.4
126.9
125.6
125.4
120.8
118.5
118.0
115.3
110.4
109.2
51.7
39.9 DMSO
39.8 DMSO
39.7 DMSO
39.5 DMSO
39.4 DMSO
39.3 DMSO
39.1 DMSO
f1 (ppm)

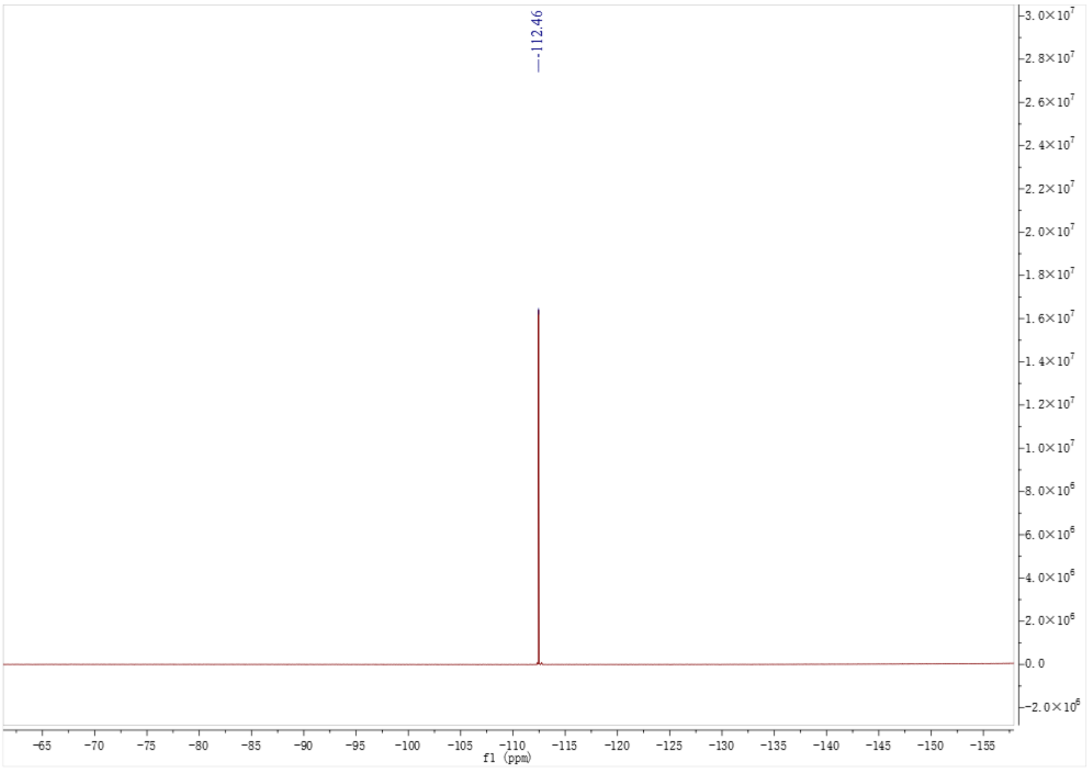

-112.46
f1 (ppm)

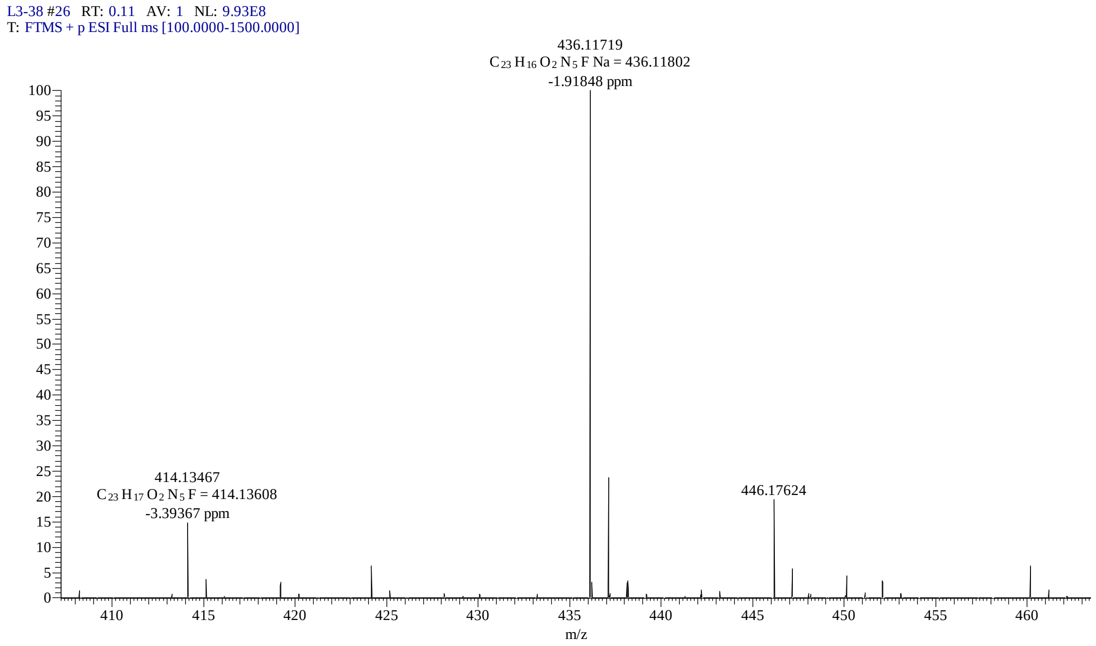

Spectra of compound **10t** (^{1}H NMR, ^{13}C NMR, HRMS)

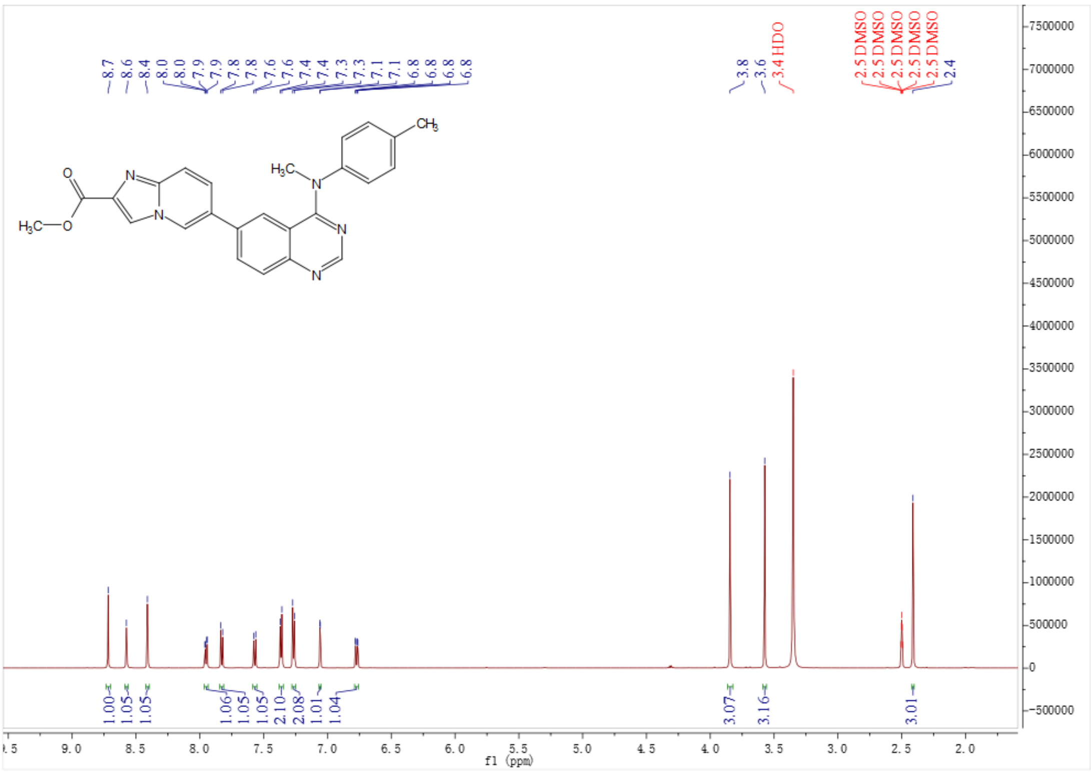

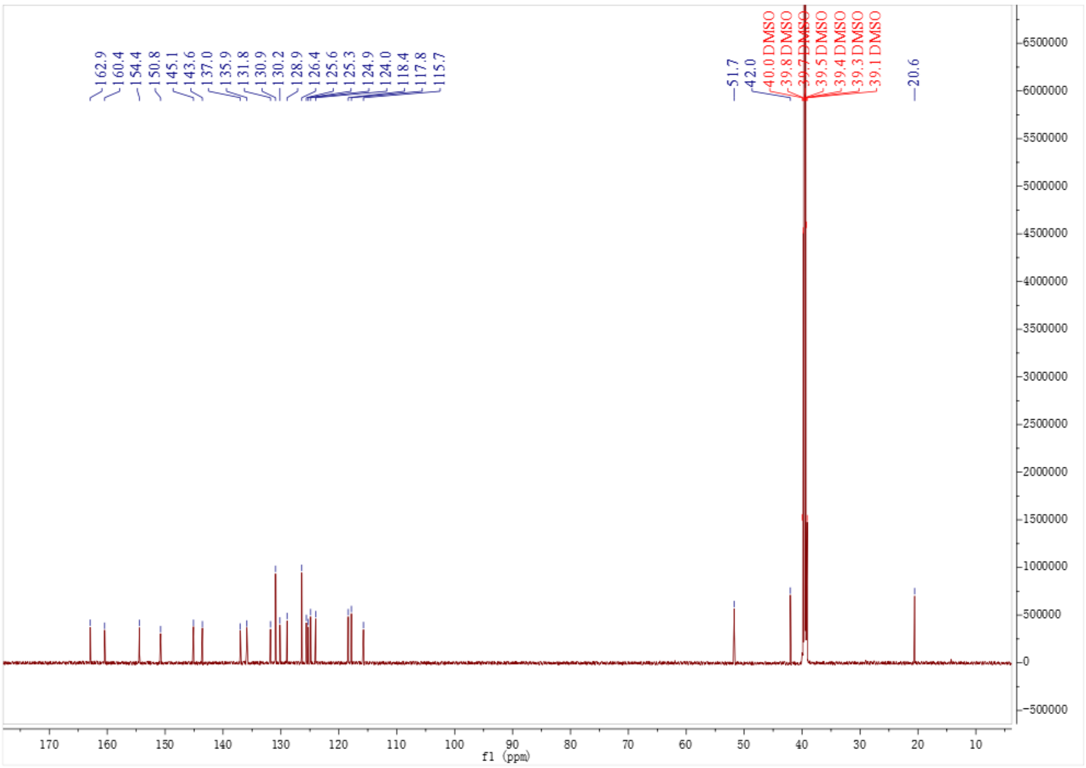
162.9
160.4
154.4
150.8
145.1
143.6
137.0
135.9
131.8
130.9
130.2
128.9
126.4
125.6
125.3
124.9
124.0
118.4
117.8
115.7
51.7
42.0
40.0 DMSO
39.8 DMSO
39.7 DMSO
39.5 DMSO
39.4 DMSO
39.3 DMSO
39.1 DMSO
20.6
f1 (ppm)

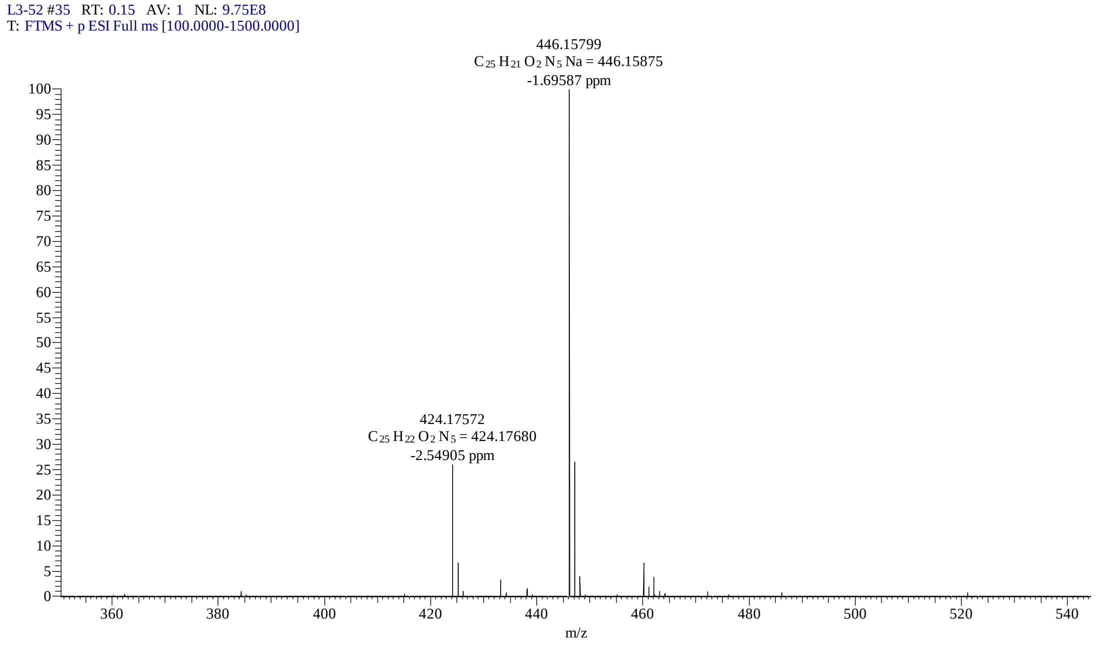
L3-52 #35 RT: 0.15 AV: 1 NL: 9.75E8
T: FTMS + p ESI Full ms [100.0000-1500.0000]
446.15799
C25 H21 O2 N5 Na = 446.15875
-1.69587 ppm
424.17572
C25 H22 O2 N5 = 424.17680
-2.54905 ppm
m/z

Spectra of compound **10u** (^{1}H NMR, ^{13}C NMR, HRMS)

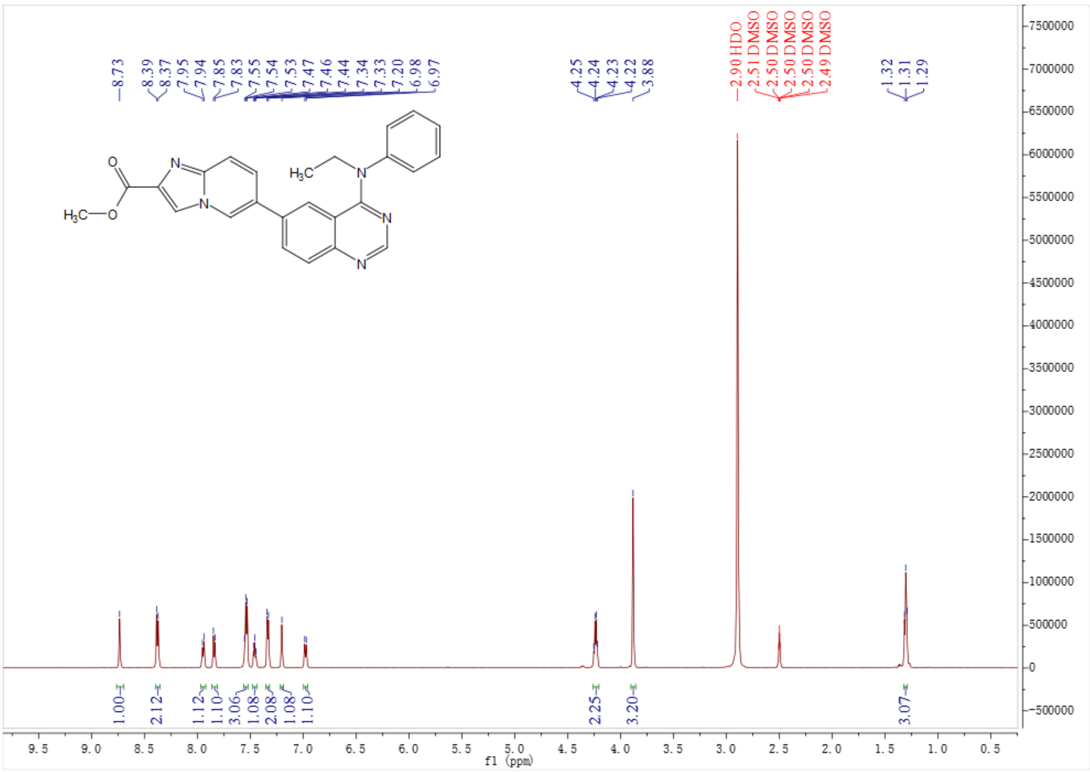

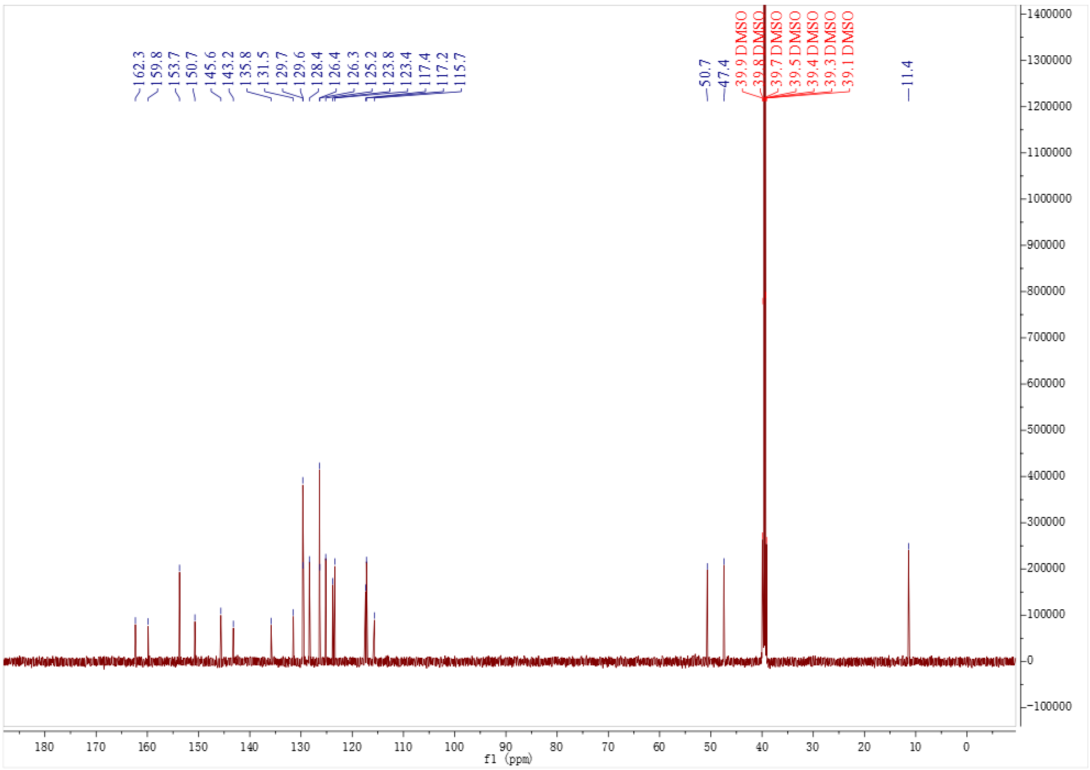

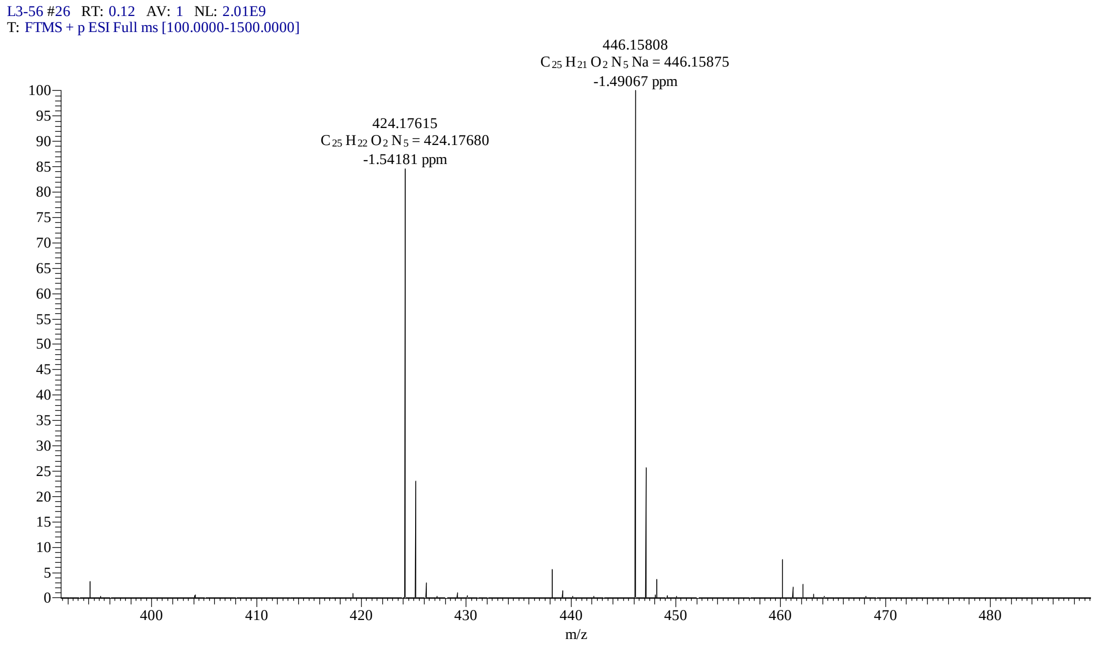

5. ^{1}H NMR and ^{13}C NMR Spectrum of the compounds 13a-13k.

Spectra of compound **13a** (^{1}H NMR, ^{13}C NMR, ^{19}F NMR, HRMS)

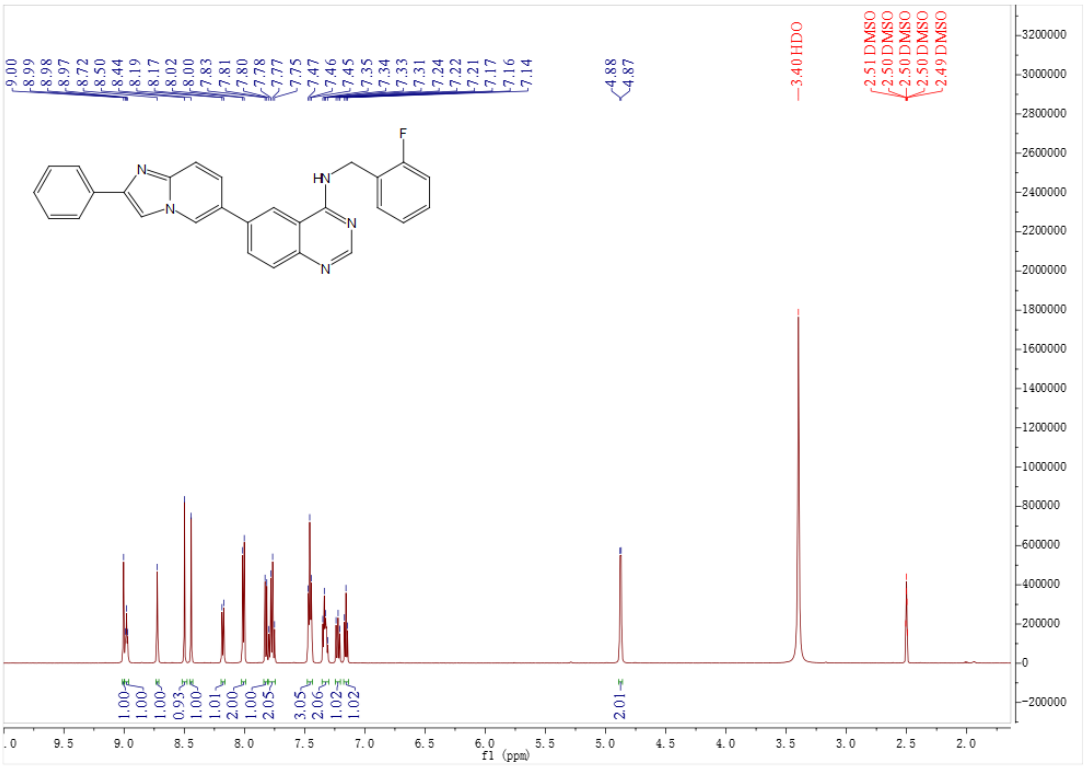

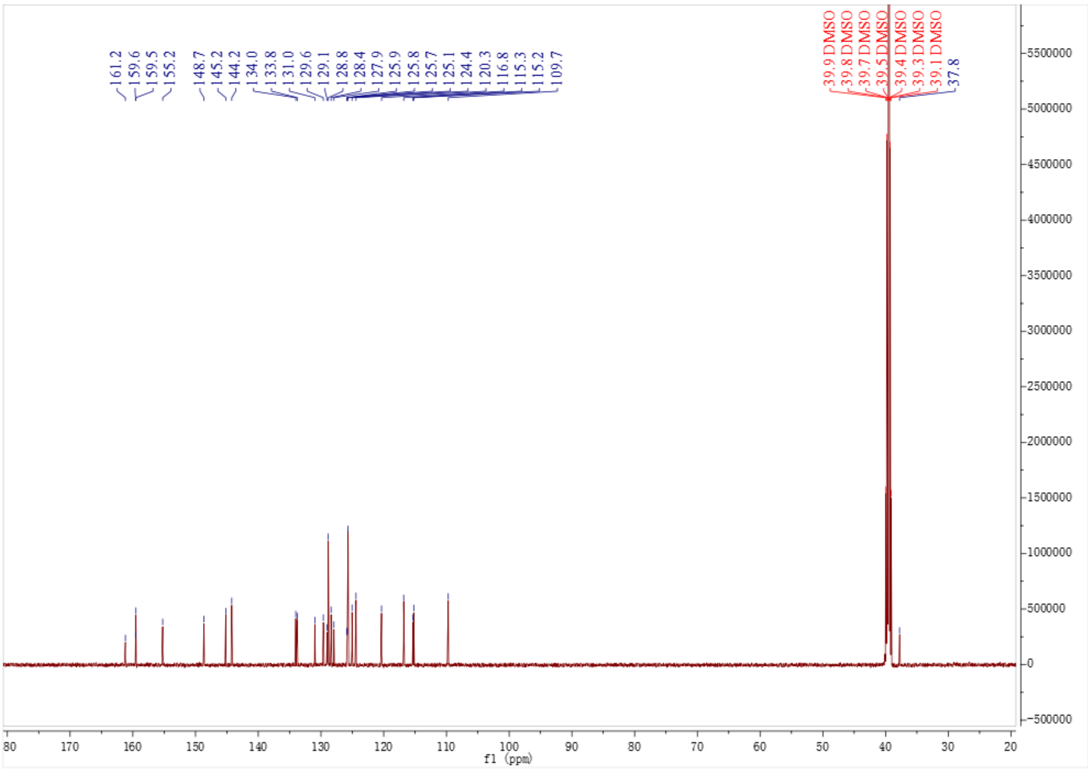
161.2
159.6
159.5
155.2
148.7
145.2
144.2
134.0
133.8
131.0
129.6
129.1
128.8
128.4
127.9
125.9
125.8
125.7
125.1
124.4
120.3
116.8
115.3
115.2
109.7
39.9 DMSO
39.8 DMSO
39.7 DMSO
39.5 DMSO
39.4 DMSO
39.3 DMSO
39.1 DMSO
37.8
f1 (ppm)

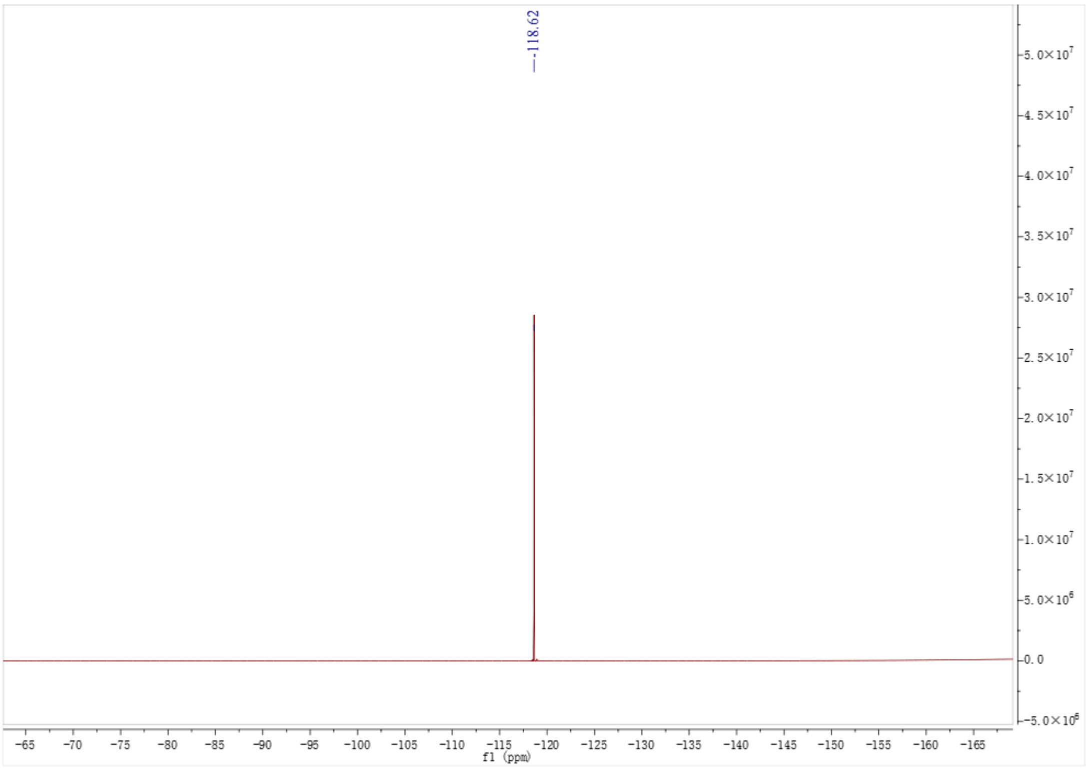
-118.62
f1 (ppm)

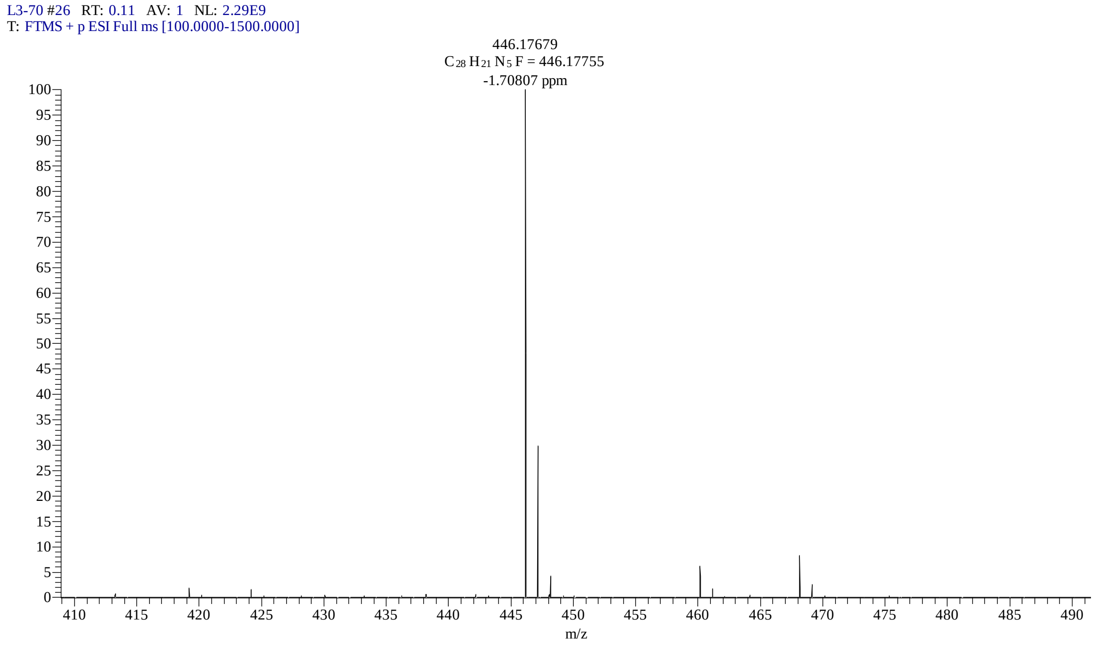

Spectra of compound **13b** (^{1}H NMR, ^{13}C NMR, ^{19}F NMR, HRMS)

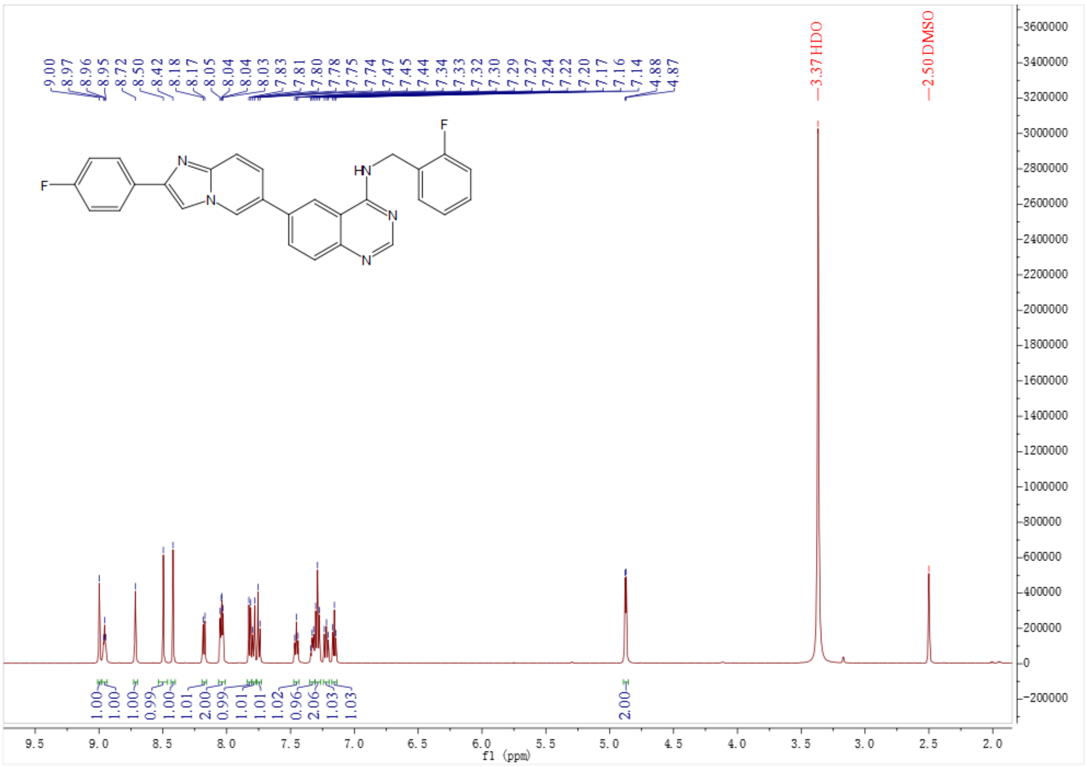

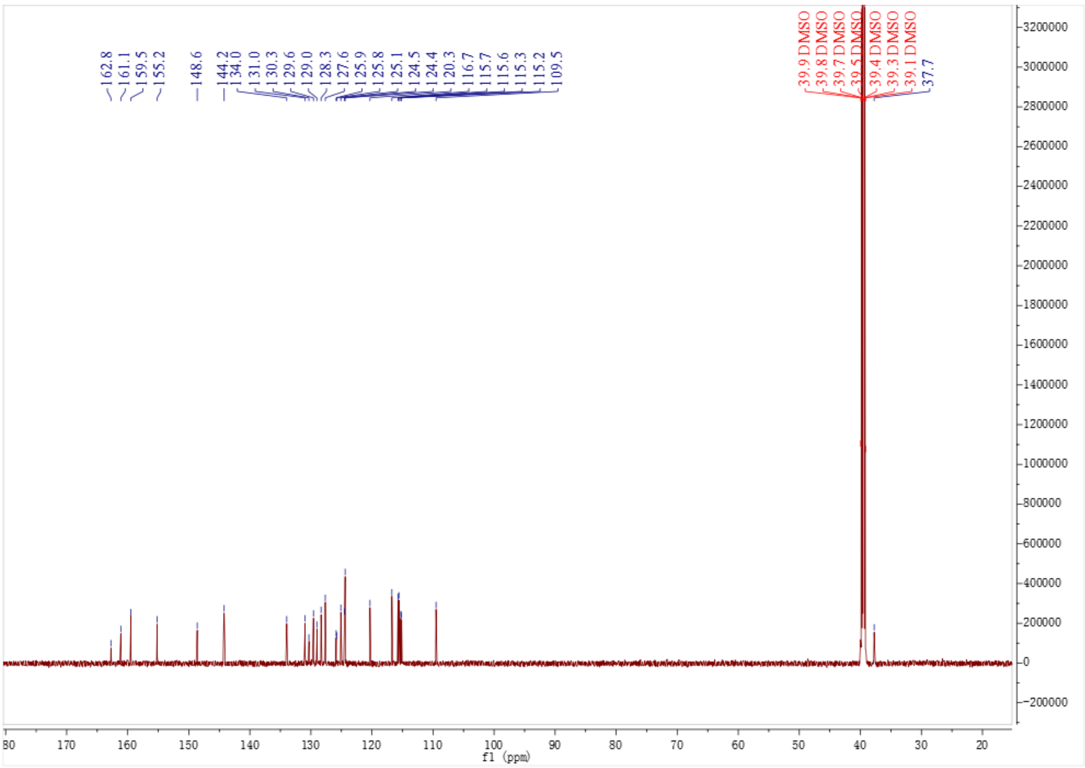
162.8
161.1
159.5
155.2
148.6
144.2
134.0
131.0
130.3
129.6
129.0
128.3
127.6
125.9
125.8
125.1
124.5
124.4
120.3
116.7
115.7
115.6
115.3
115.2
109.5
39.9 DMSO
39.8 DMSO
39.7 DMSO
39.5 DMSO
39.4 DMSO
39.3 DMSO
39.1 DMSO
37.7
f1 (ppm)

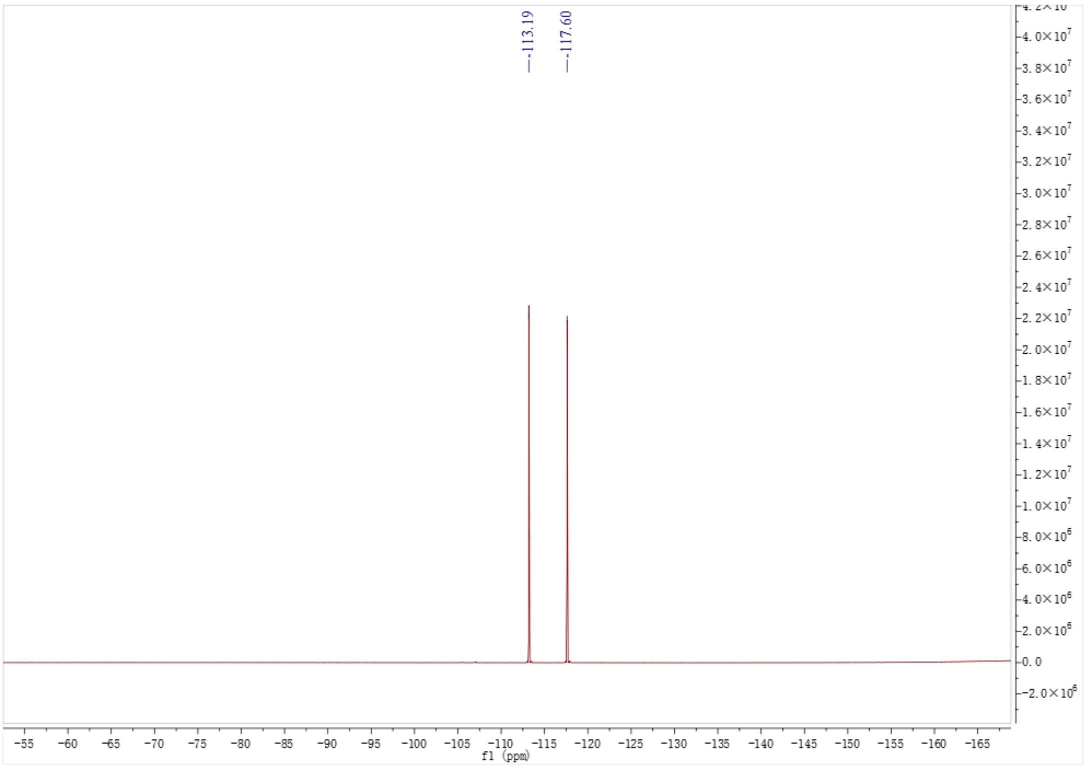
-113.19
-117.60
f1 (ppm)

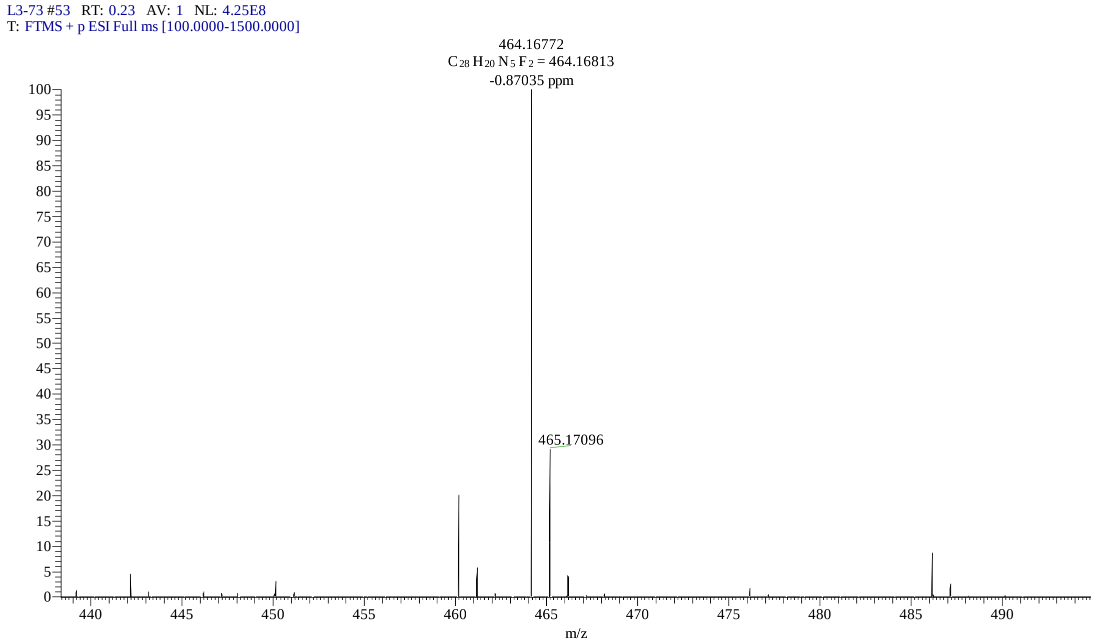

Spectra of compound **13c** (^{1}H NMR, ^{13}C NMR, ^{19}F NMR, HRMS)

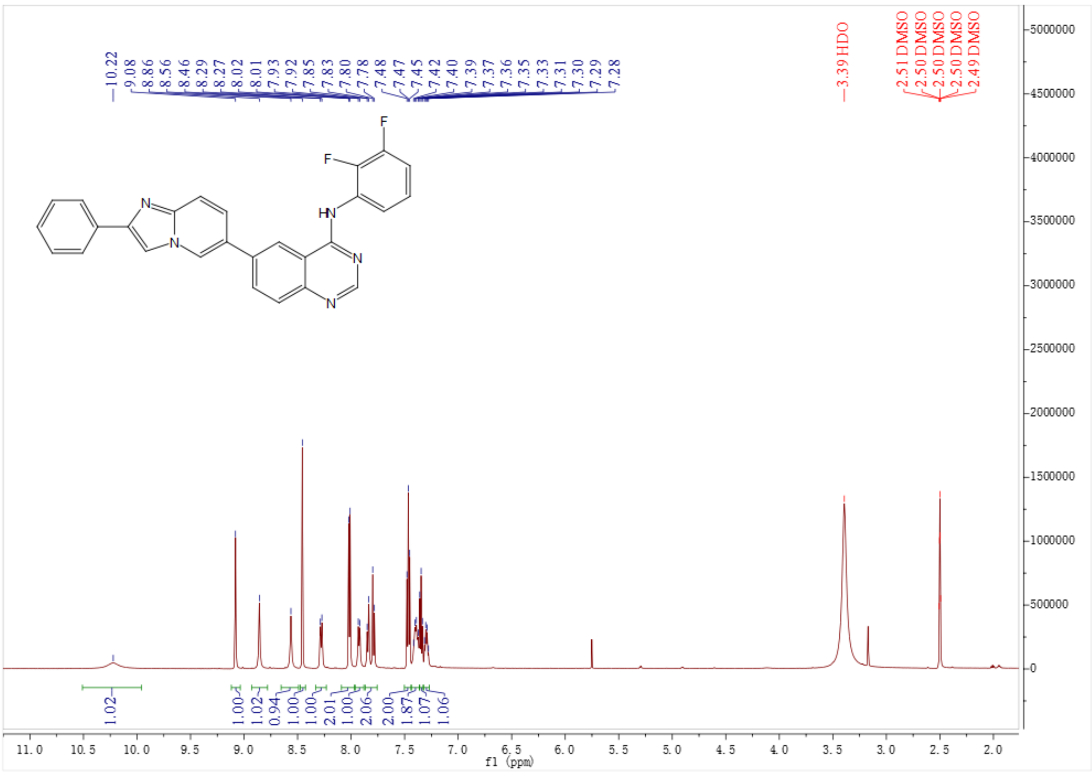

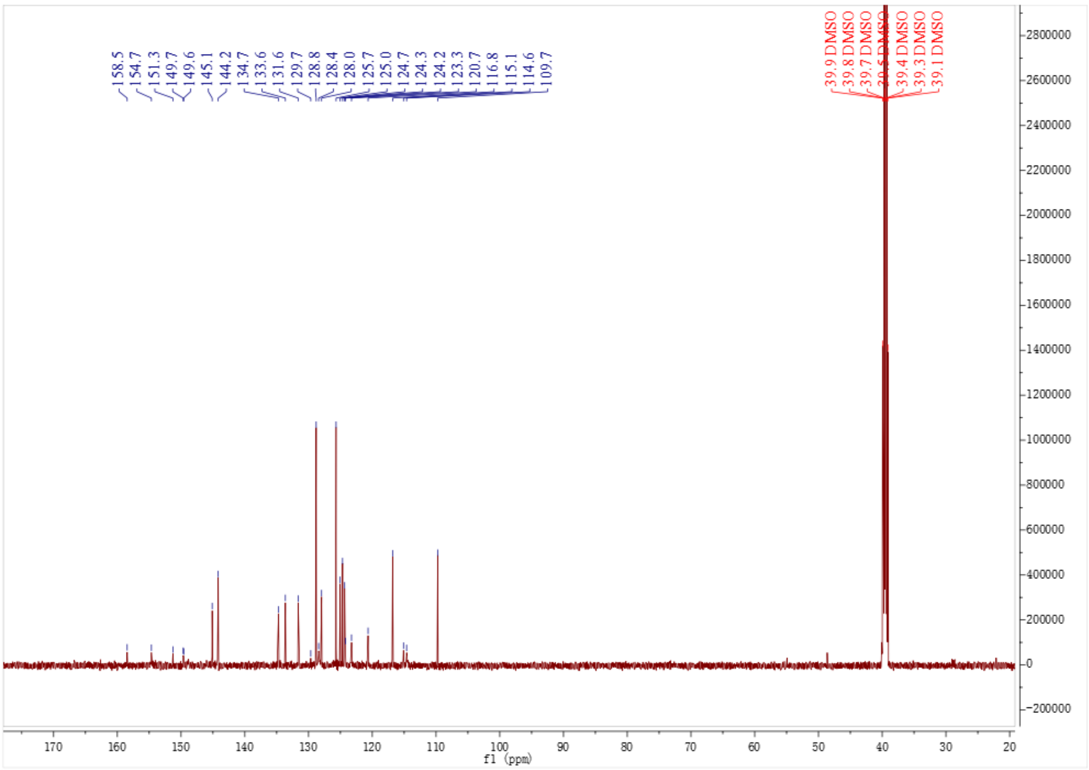
158.5
154.7
151.3
149.7
149.6
145.1
144.2
134.7
133.6
131.6
129.7
128.8
128.4
128.0
125.7
125.0
124.7
124.3
124.2
123.3
120.7
116.8
115.1
114.6
109.7
39.9 DMSO
39.8 DMSO
39.7 DMSO
39.4 DMSO
39.3 DMSO
39.1 DMSO
f1 (ppm)

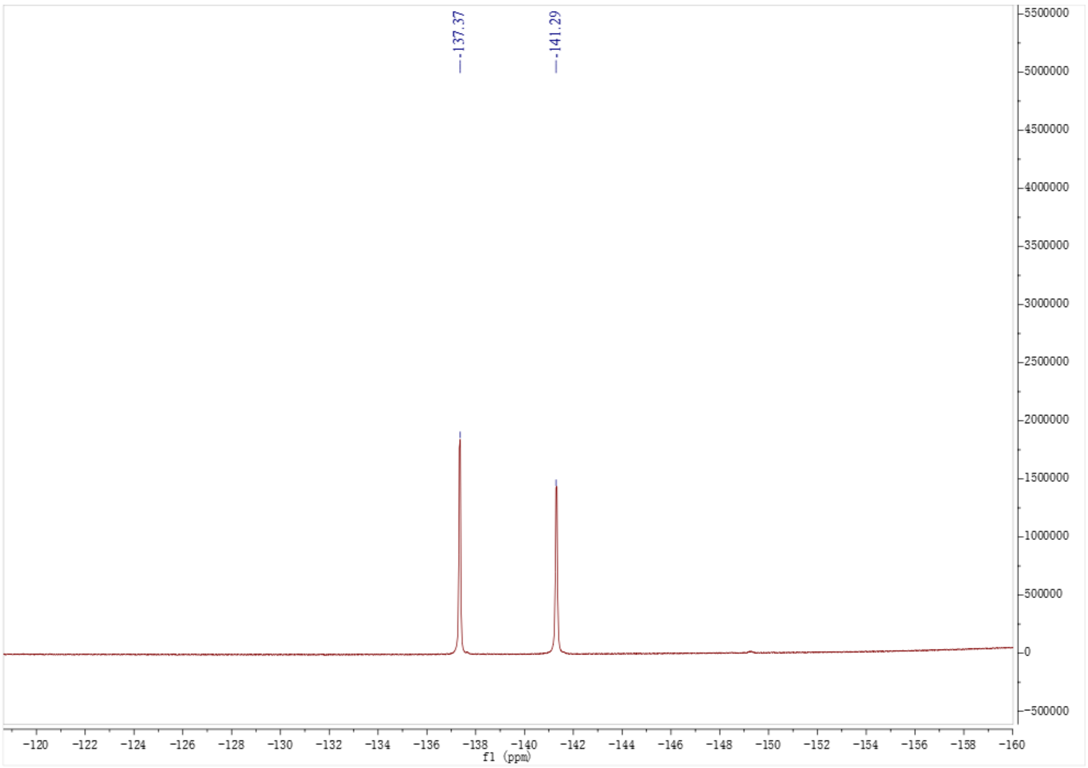
-137.37
-141.29
f1 (ppm)

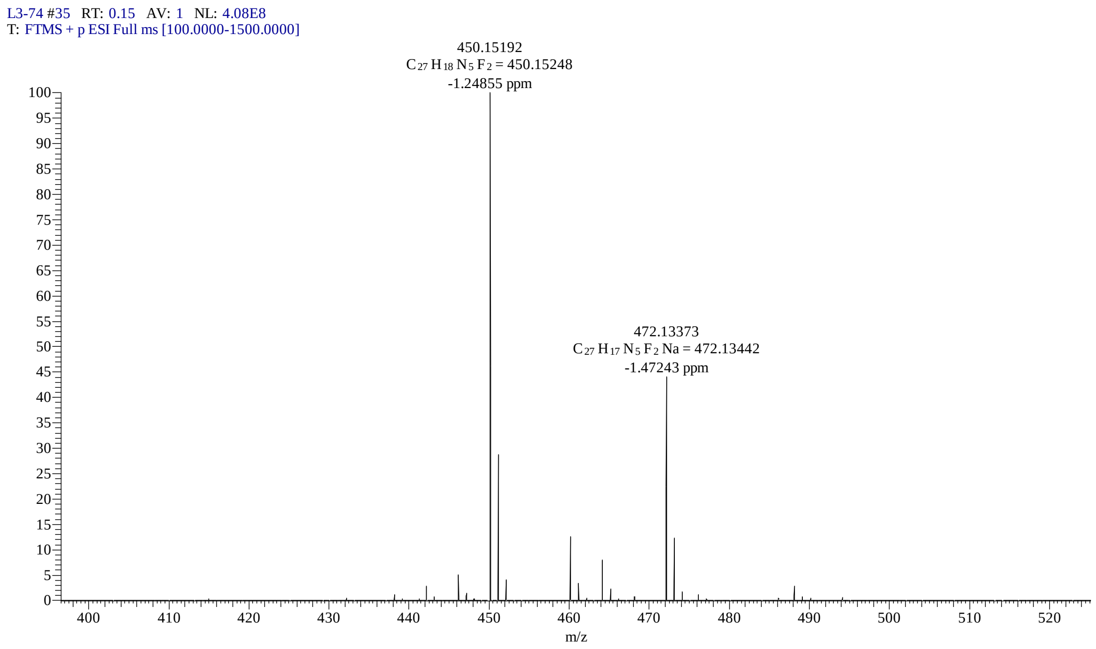

Spectra of compound **13d** (^{1}H NMR, ^{13}C NMR, ^{19}F NMR, HRMS)

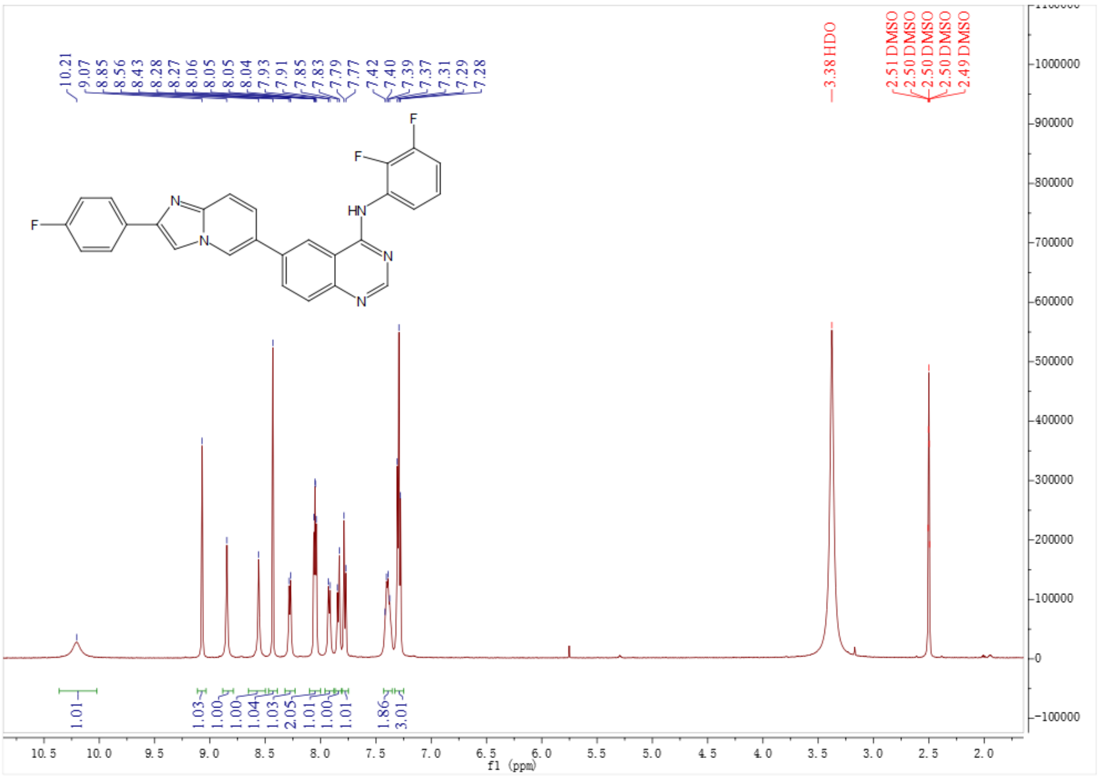

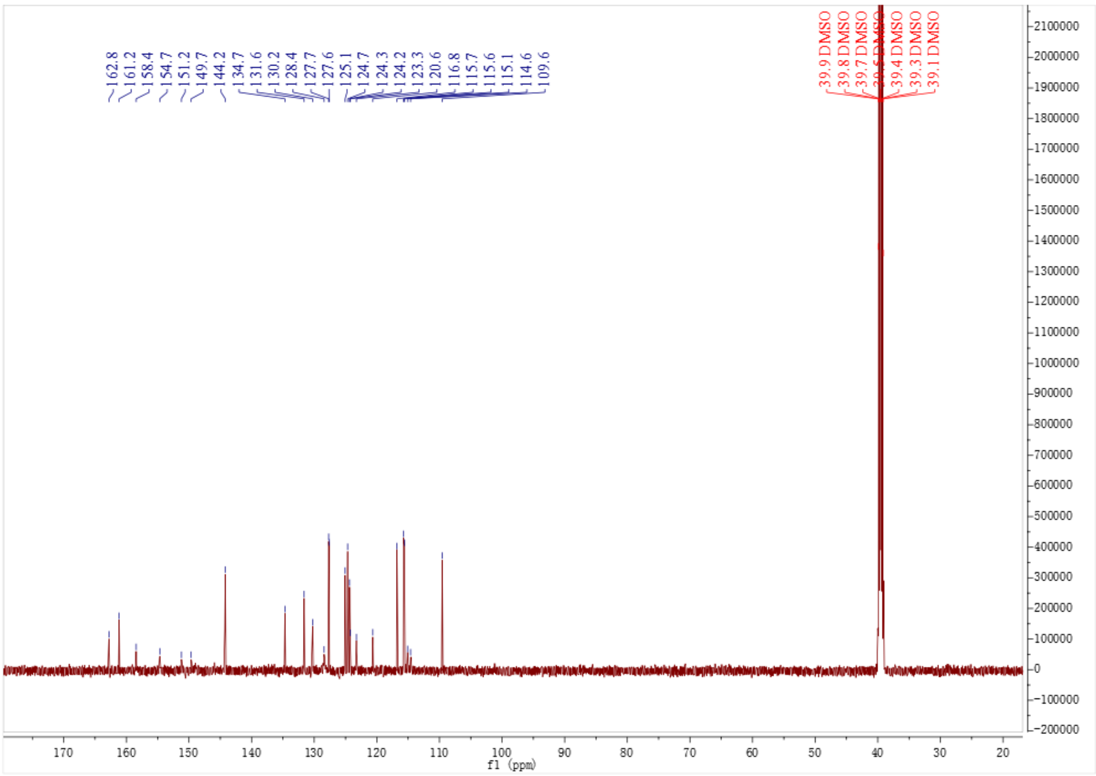

162.8
161.2
158.4
154.7
151.2
149.7
144.2
134.7
131.6
130.2
128.4
127.7
127.6
125.1
124.7
124.3
124.2
123.3
120.6
116.8
115.7
115.6
115.1
114.6
109.6
39.9 DMSO
39.8 DMSO
39.7 DMSO
39.4 DMSO
39.3 DMSO
39.1 DMSO
f1 (ppm)

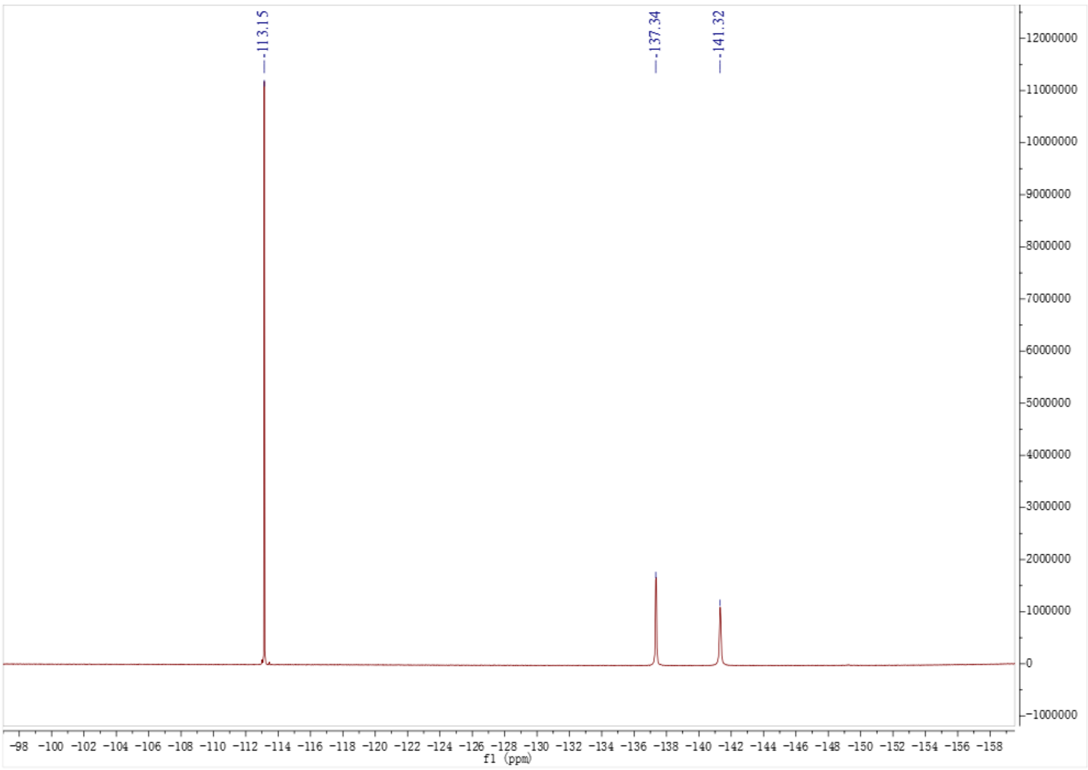

-113.15
-137.34
-141.32
f1 (ppm)

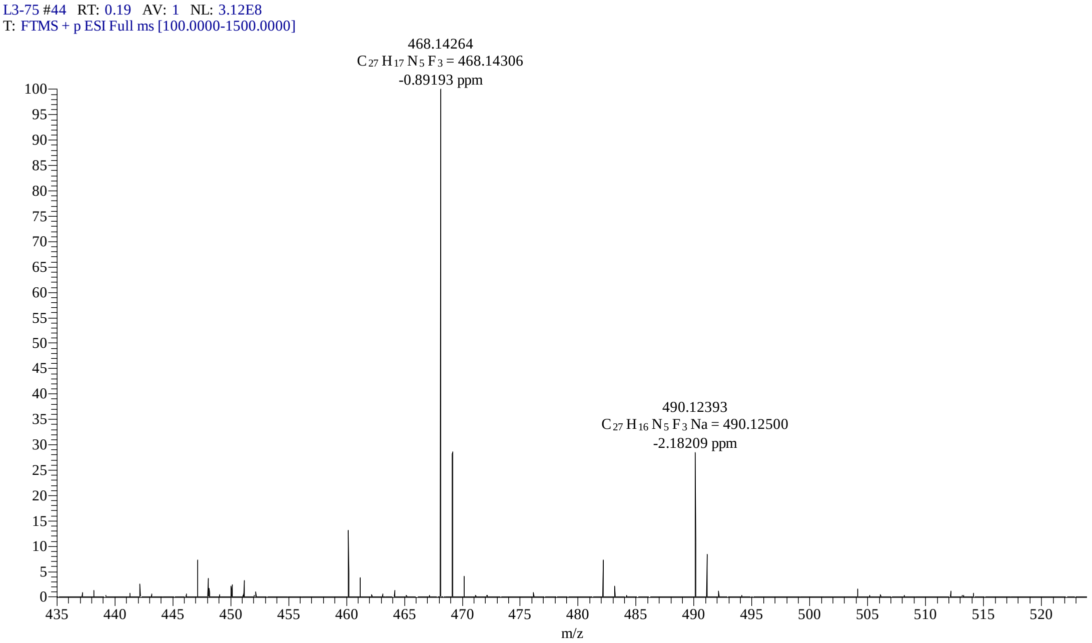

Spectra of compound **13e** (^{1}H NMR, ^{13}C NMR, ^{19}F NMR, HRMS)

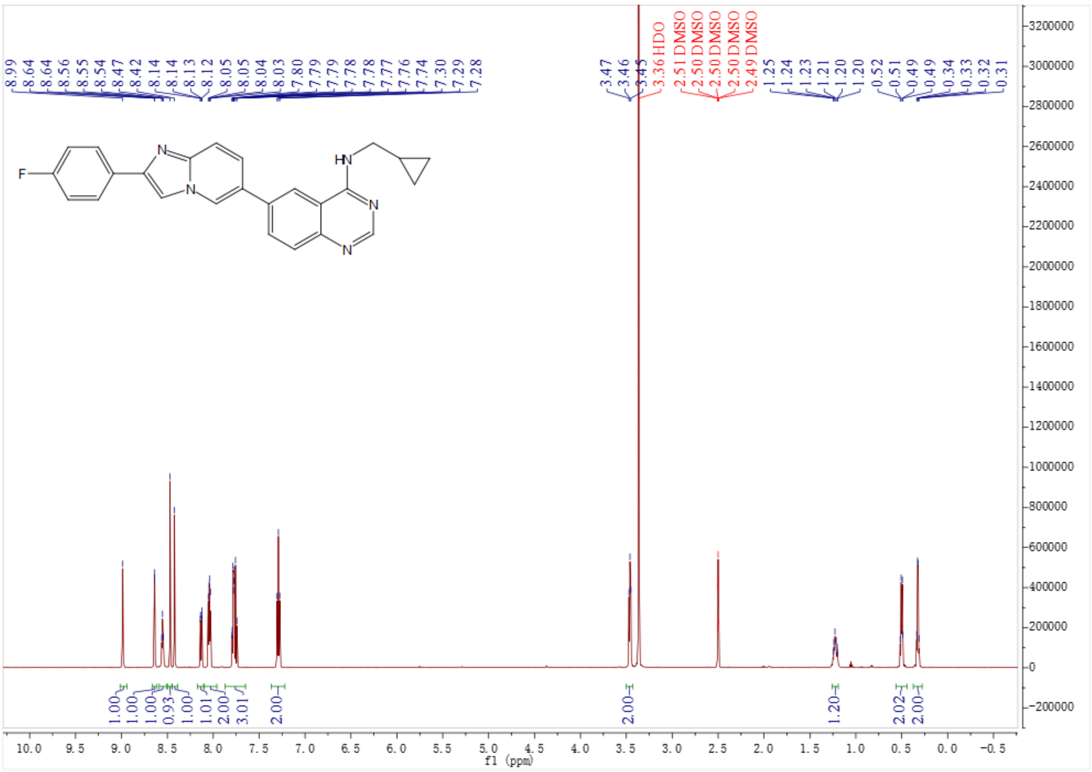

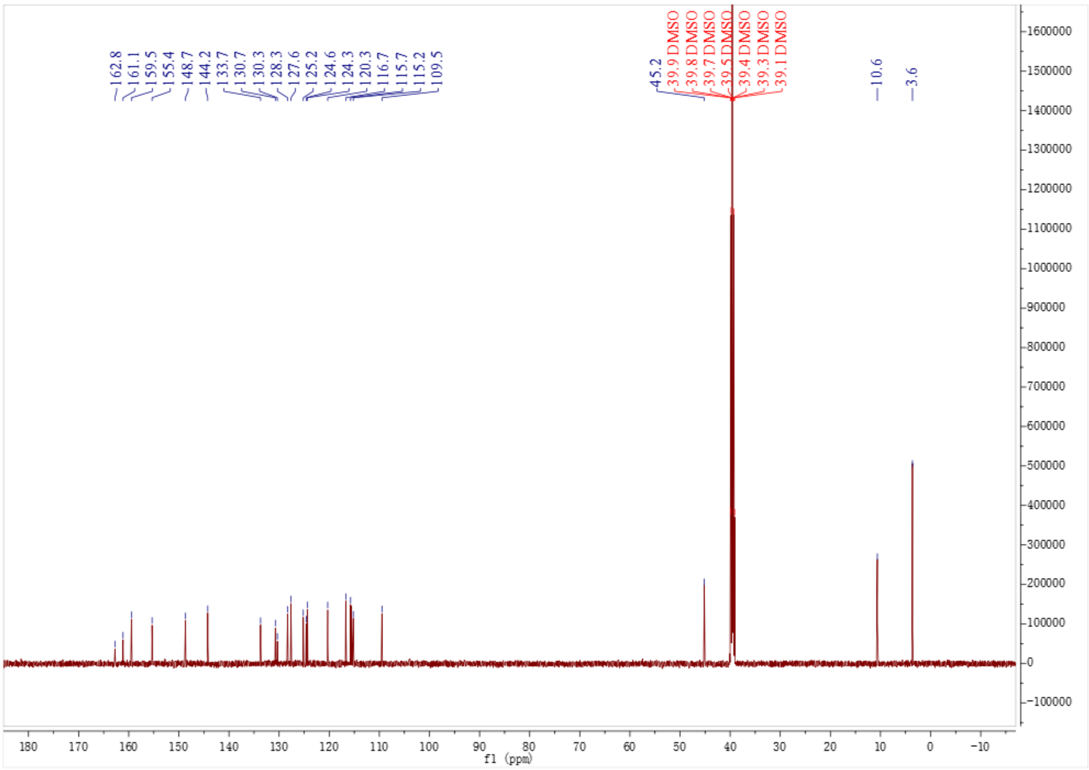
162.8
161.1
159.5
155.4
148.7
144.2
133.7
130.7
130.3
128.3
127.6
125.2
124.6
124.3
120.3
116.7
115.7
115.2
109.5
45.2
39.9 DMSO
39.8 DMSO
39.7 DMSO
39.5 DMSO
39.4 DMSO
39.3 DMSO
39.1 DMSO
10.6
3.6
f1 (ppm)

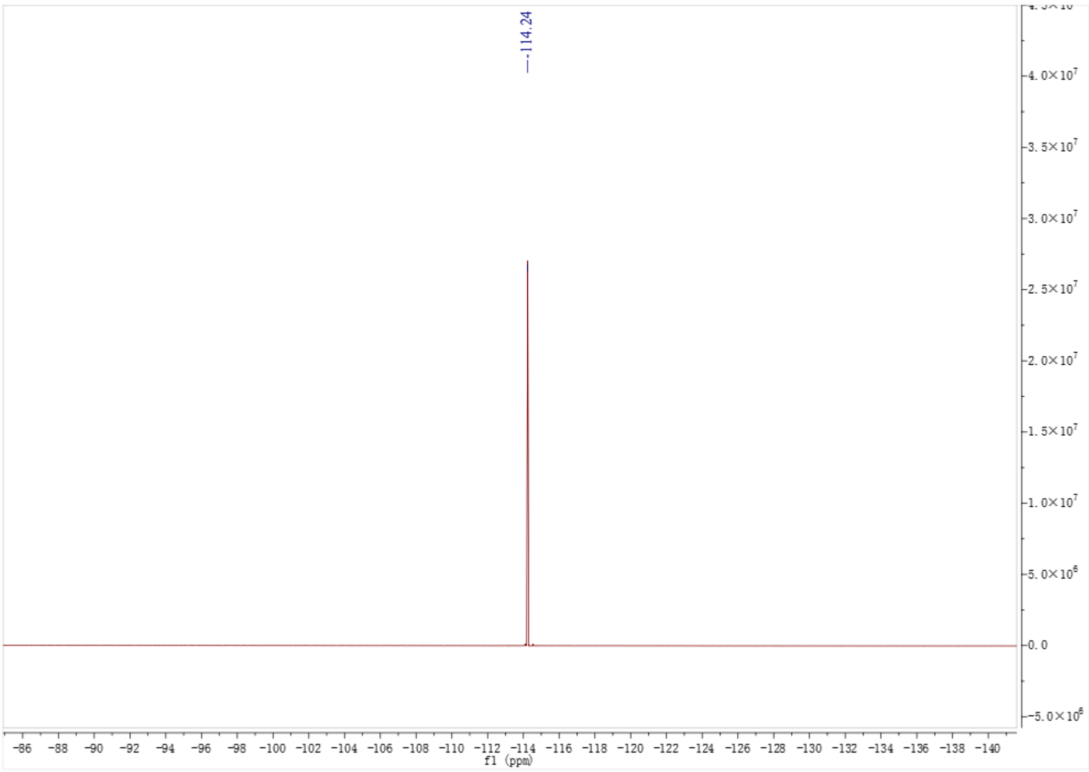
-114.24
f1 (ppm)

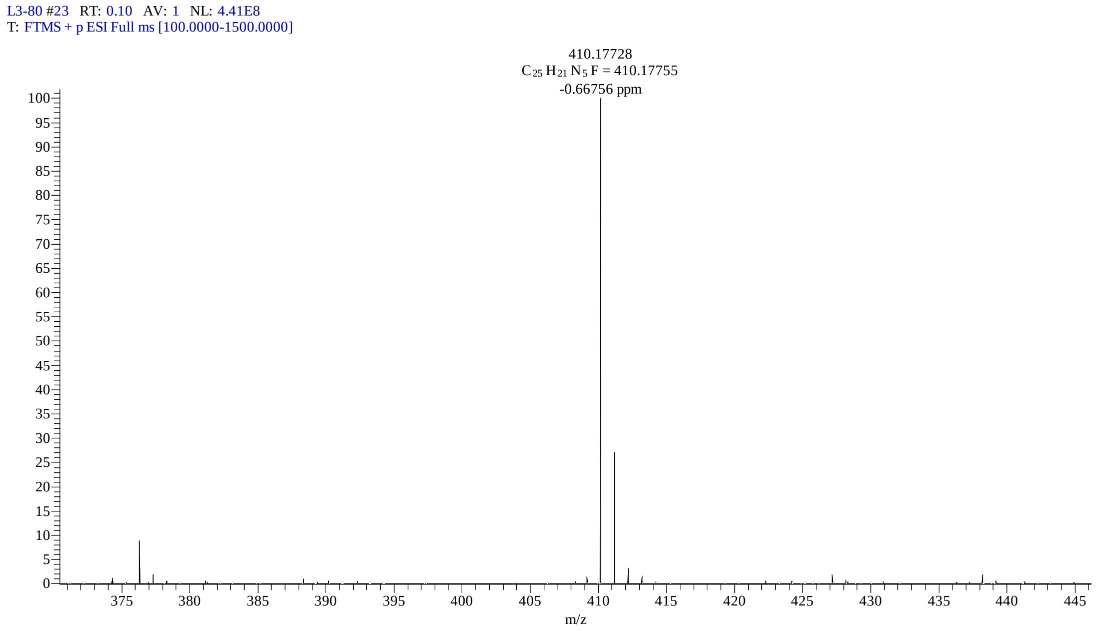

Spectra of compound **13f** (^{1}H NMR, ^{13}C NMR, HRMS)

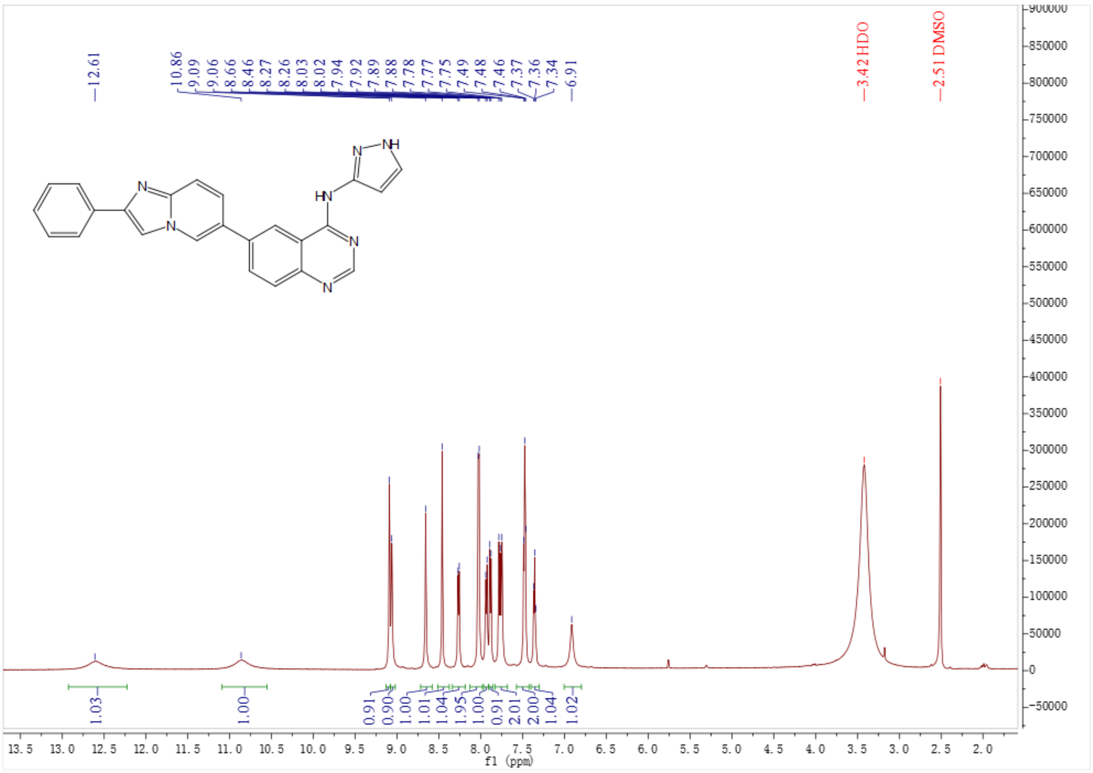

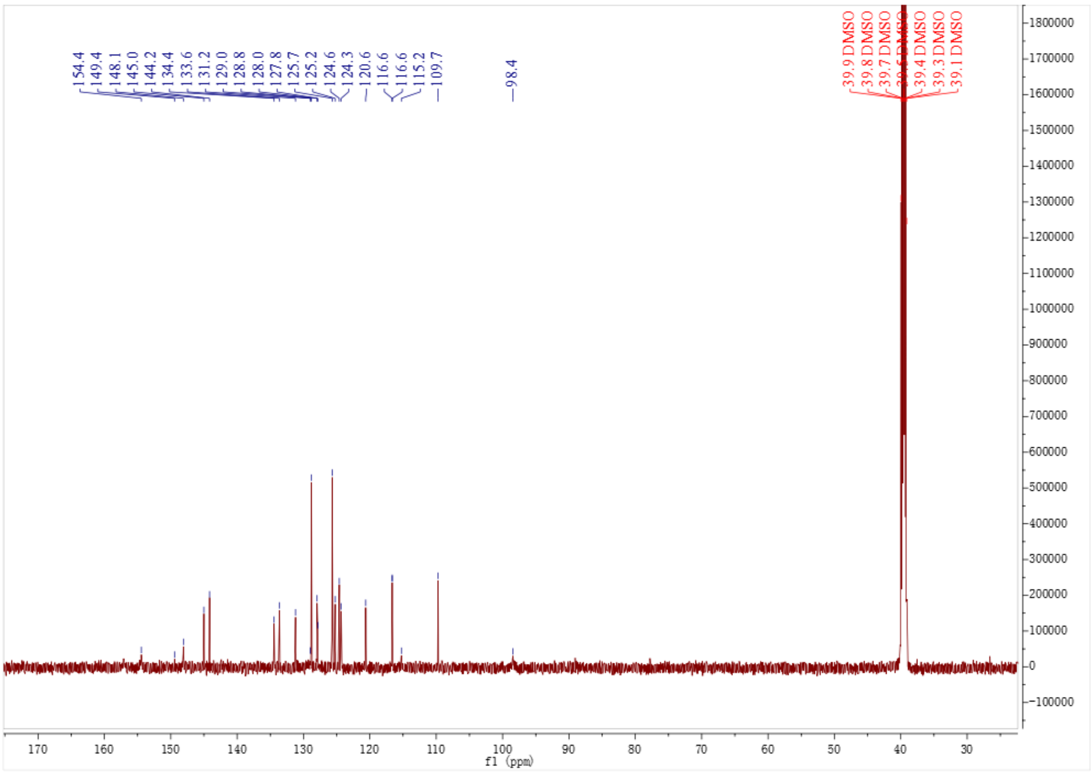
154.4
149.4
148.1
145.0
144.2
134.4
133.6
131.2
129.0
128.8
128.0
127.8
125.7
125.2
124.6
124.3
120.6
116.6
116.6
115.2
109.7
98.4
39.9 DMSO
39.8 DMSO
39.7 DMSO
39.4 DMSO
39.3 DMSO
39.1 DMSO
f1 (ppm)

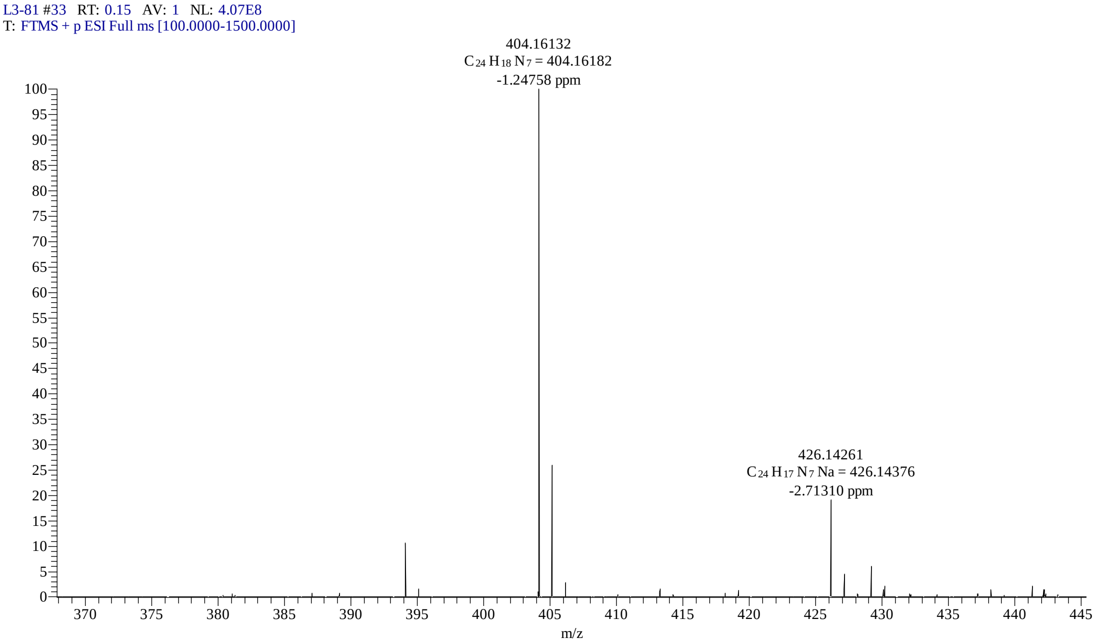
L3-81 #33 RT: 0.15 AV: 1 NL: 4.07E8
T: FTMS + p ESI Full ms [100.0000-1500.0000]
404.16132
C24 H18 N7 = 404.16182
-1.24758 ppm
426.14261
C24 H17 N7 Na = 426.14376
-2.71310 ppm
m/z

Spectra of compound **13g** (^{1}H NMR, ^{13}C NMR, HRMS)

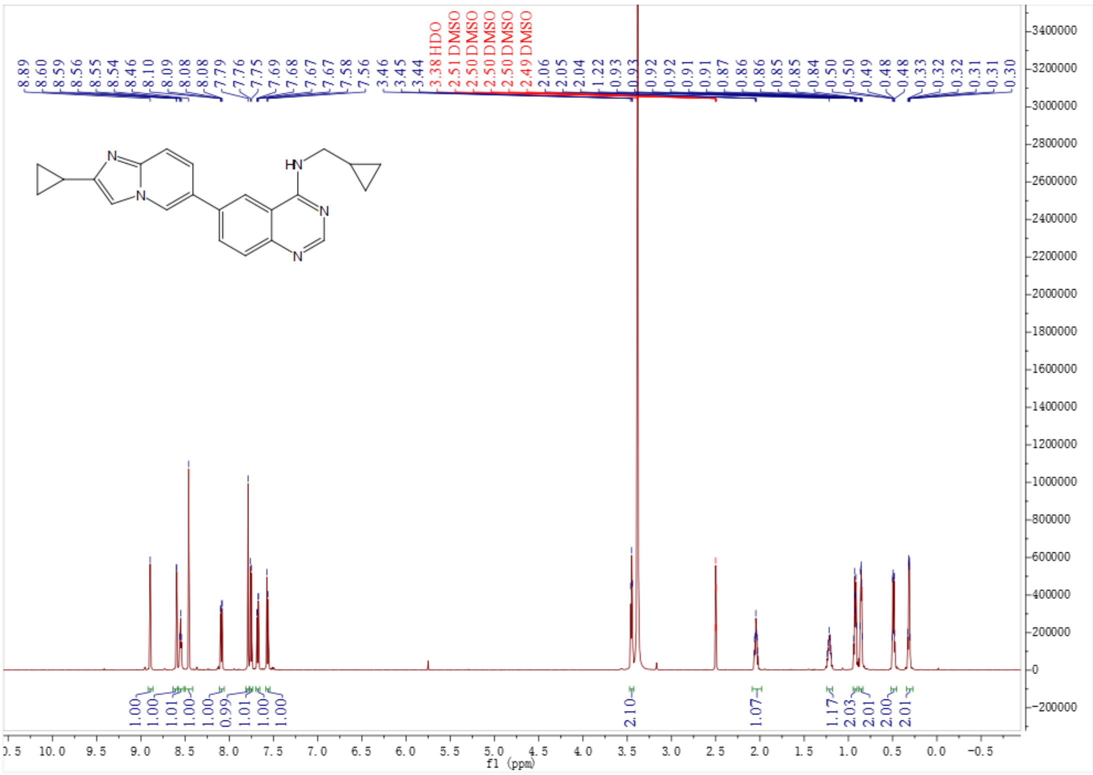

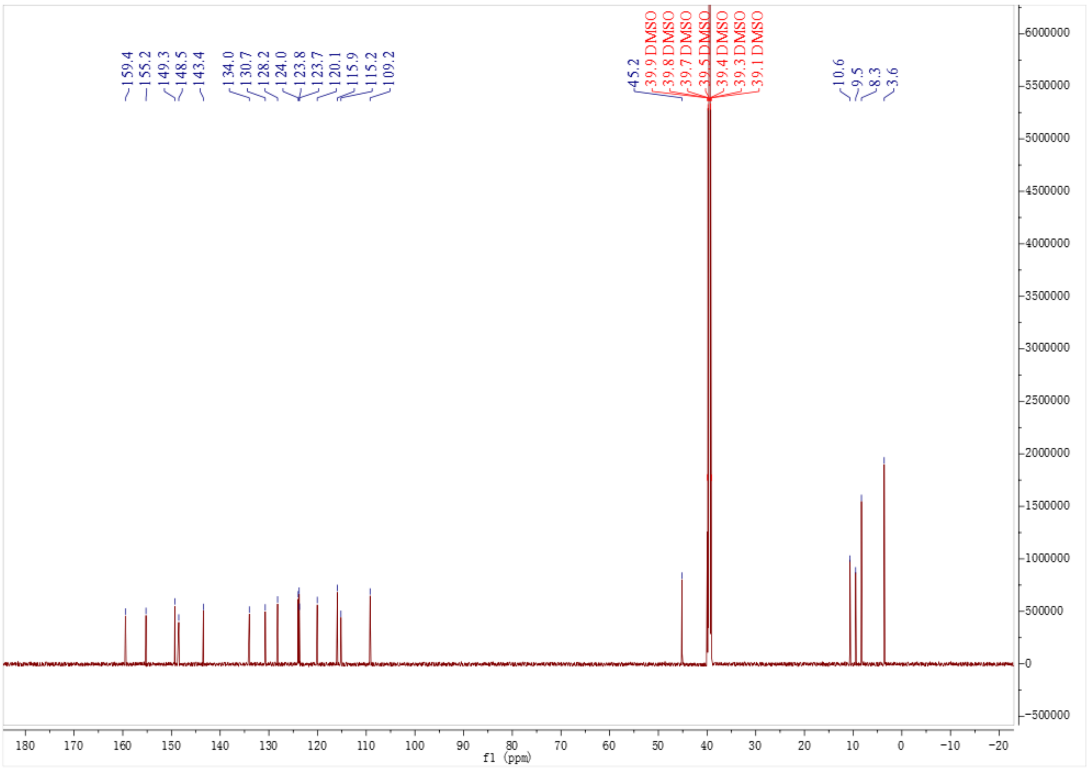

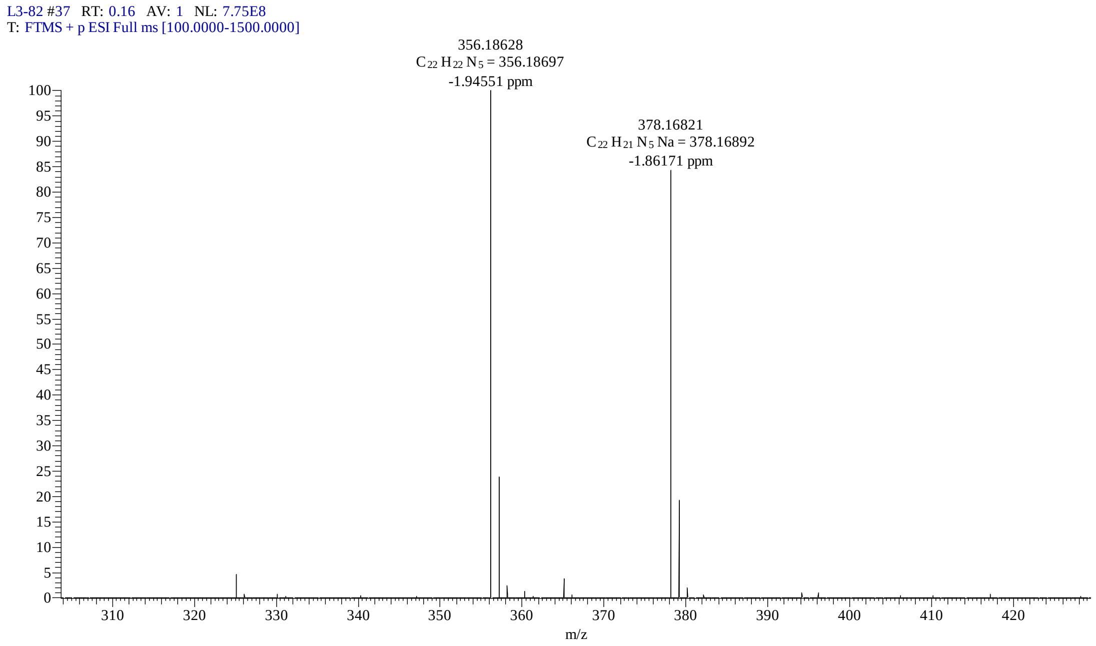

Spectra of compound **13h** (^{1}H NMR, ^{13}C NMR, HRMS)

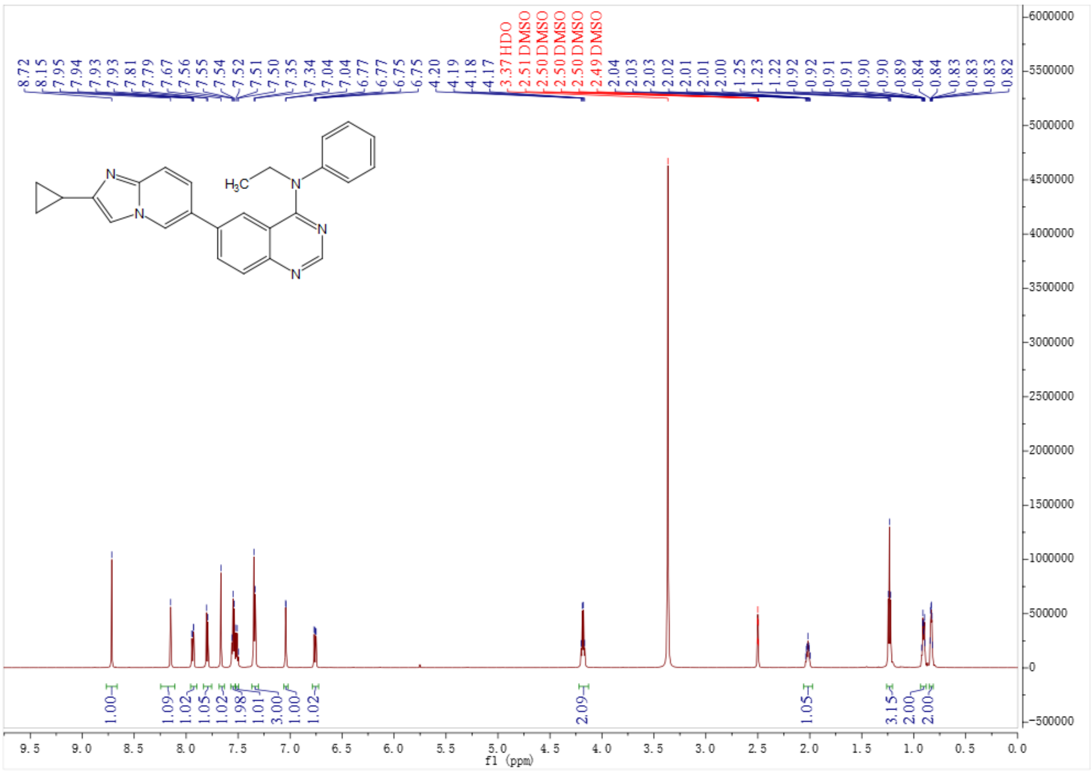

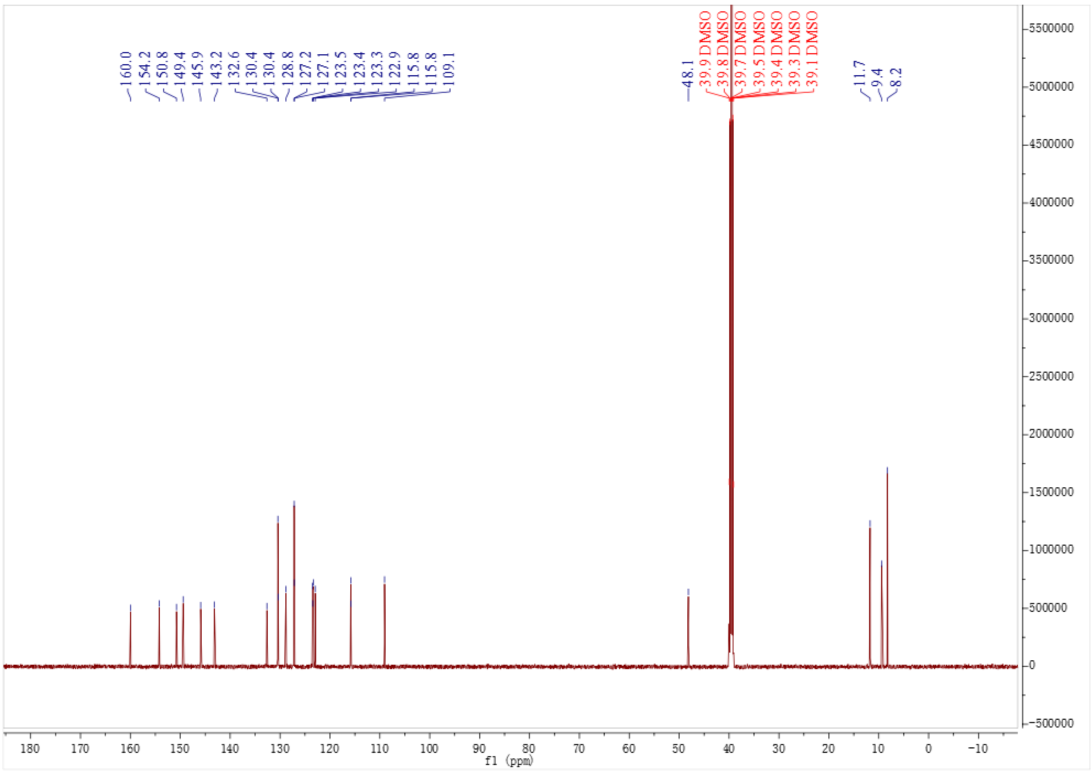
160.0
154.2
150.8
149.4
145.9
143.2
132.6
130.4
130.4
128.8
127.2
127.1
123.5
123.4
123.3
122.9
115.8
115.8
109.1
48.1
39.9 DMSO
39.8 DMSO
39.7 DMSO
39.5 DMSO
39.4 DMSO
39.3 DMSO
39.1 DMSO
11.7
9.4
8.2
f1 (ppm)

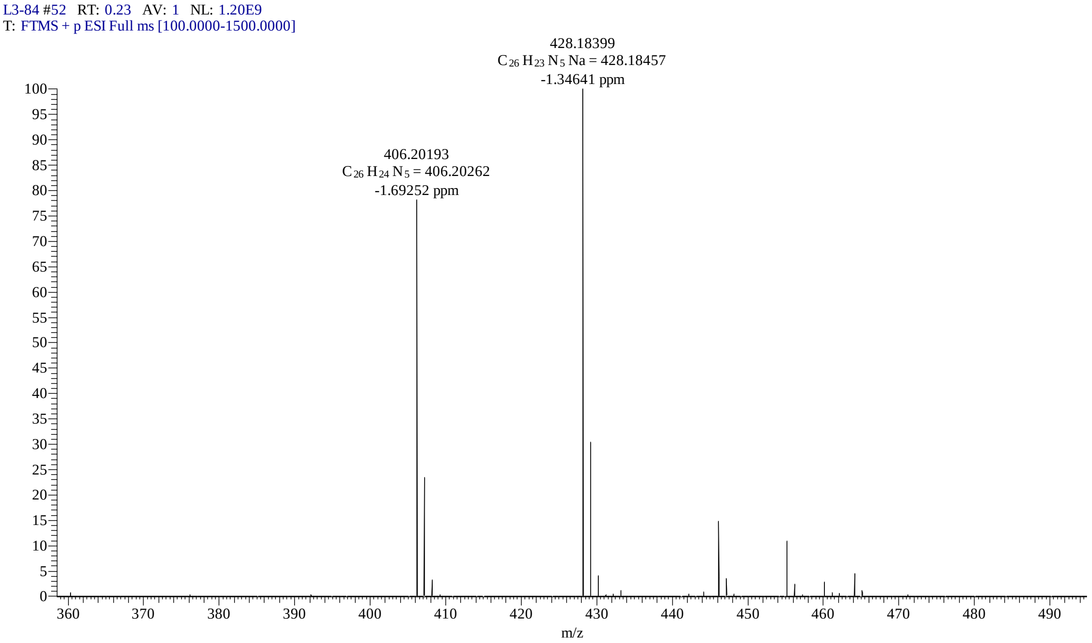
L3-84 #52 RT: 0.23 AV: 1 NL: 1.20E9
T: FTMS + p ESI Full ms [100.0000-1500.0000]
428.18399
C26 H23 N5 Na = 428.18457
-1.34641 ppm
406.20193
C26 H24 N5 = 406.20262
-1.69252 ppm
m/z

Spectra of compound **13i** (^{1}H NMR, ^{13}C NMR, HRMS)

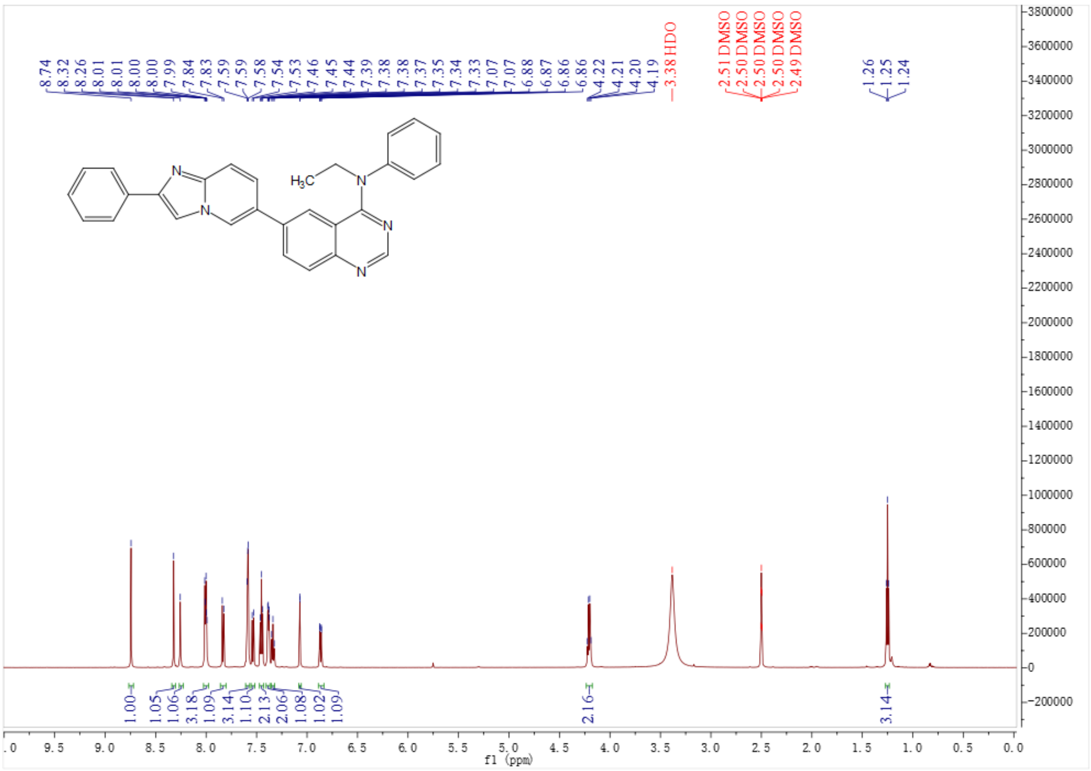

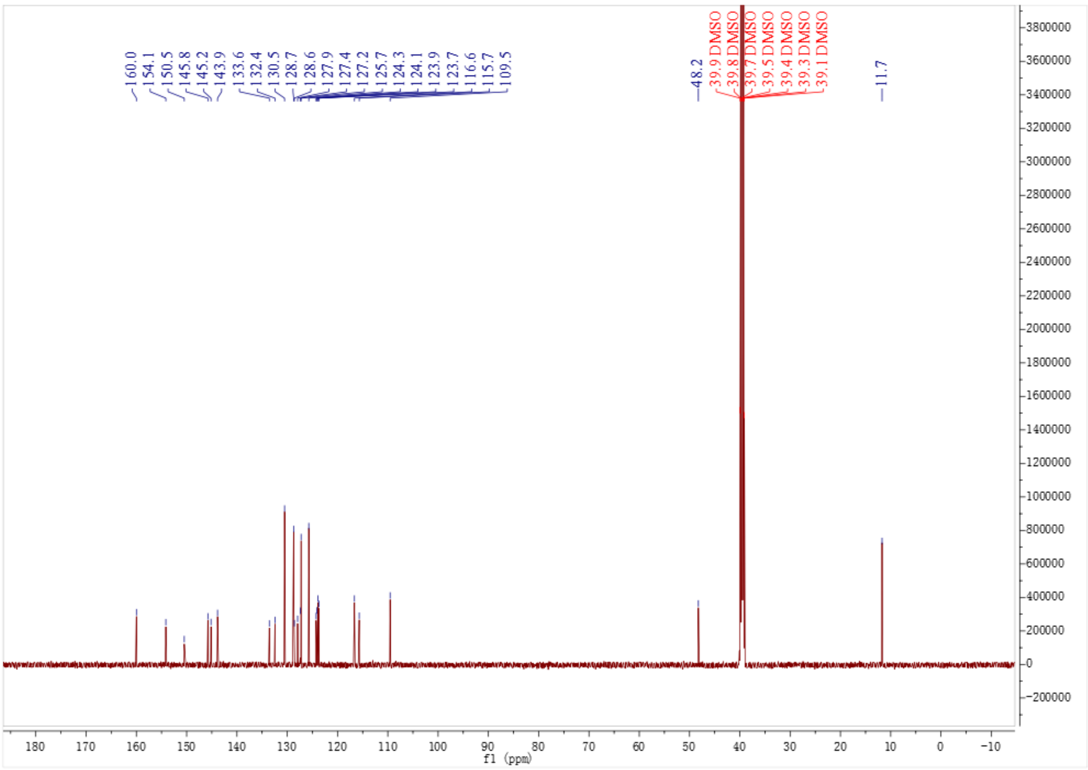

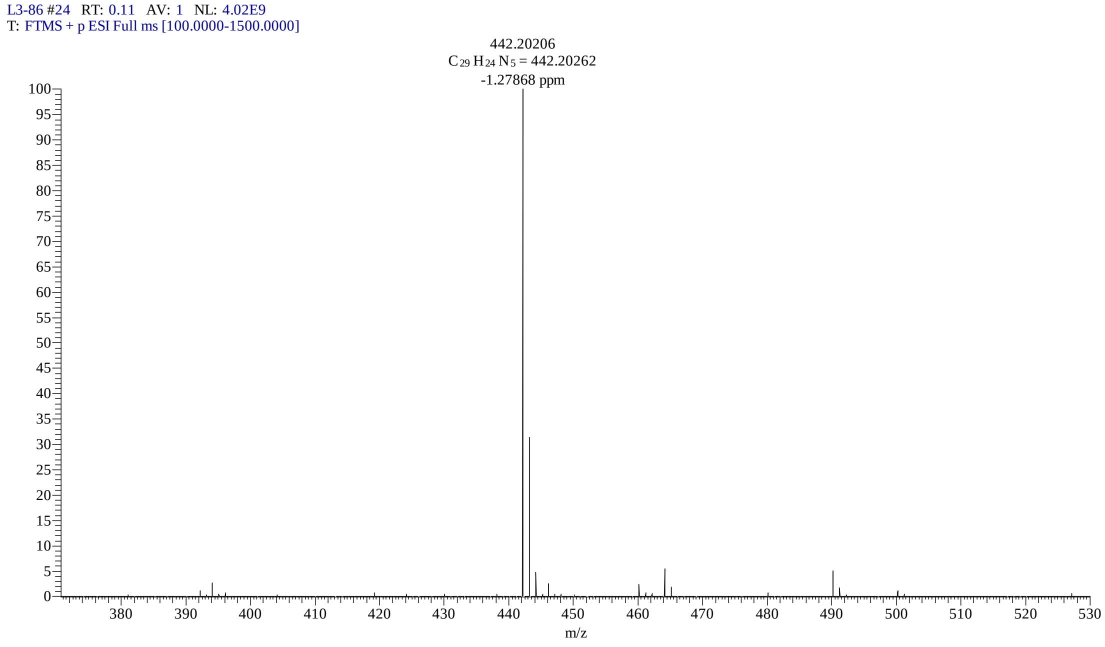

Spectra of compound **13j** (^{1}H NMR, ^{13}C NMR, HRMS)

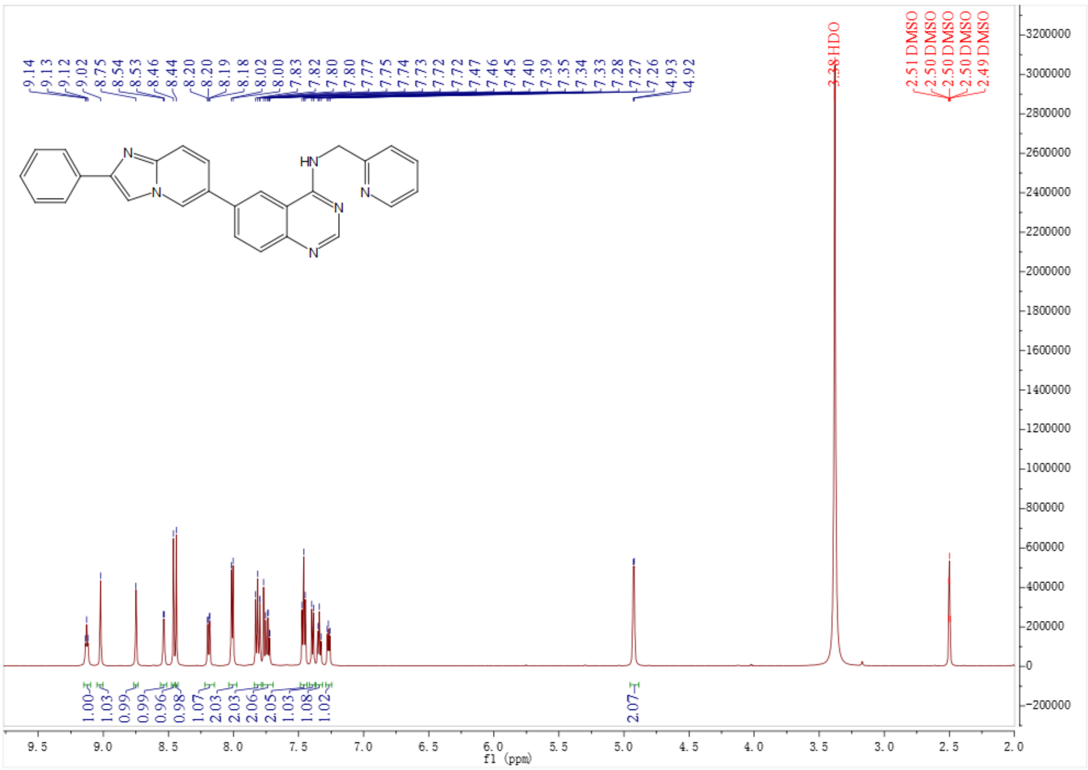

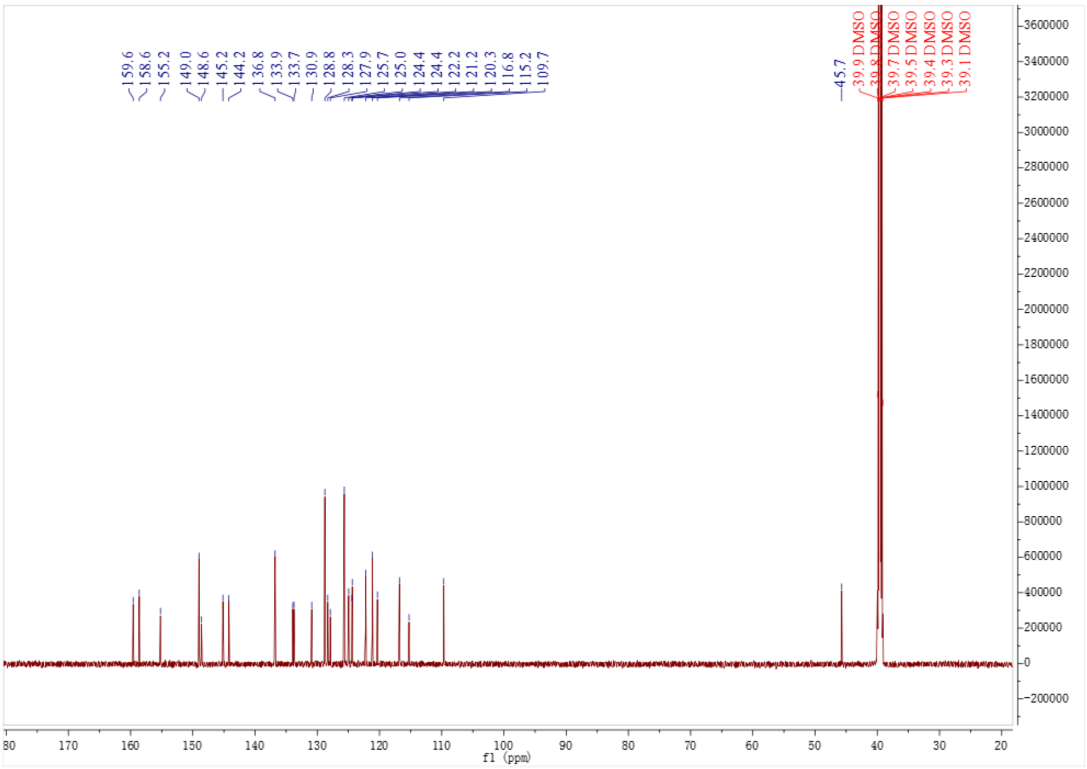

159.6
158.6
155.2
149.0
148.6
145.2
144.2
136.8
133.9
133.7
130.9
128.8
128.3
127.9
125.7
125.0
124.4
124.4
122.2
121.2
120.3
116.8
115.2
109.7
45.7
39.9 DMSO
39.8 DMSO
39.7 DMSO
39.5 DMSO
39.4 DMSO
39.3 DMSO
39.1 DMSO
f1 (ppm)

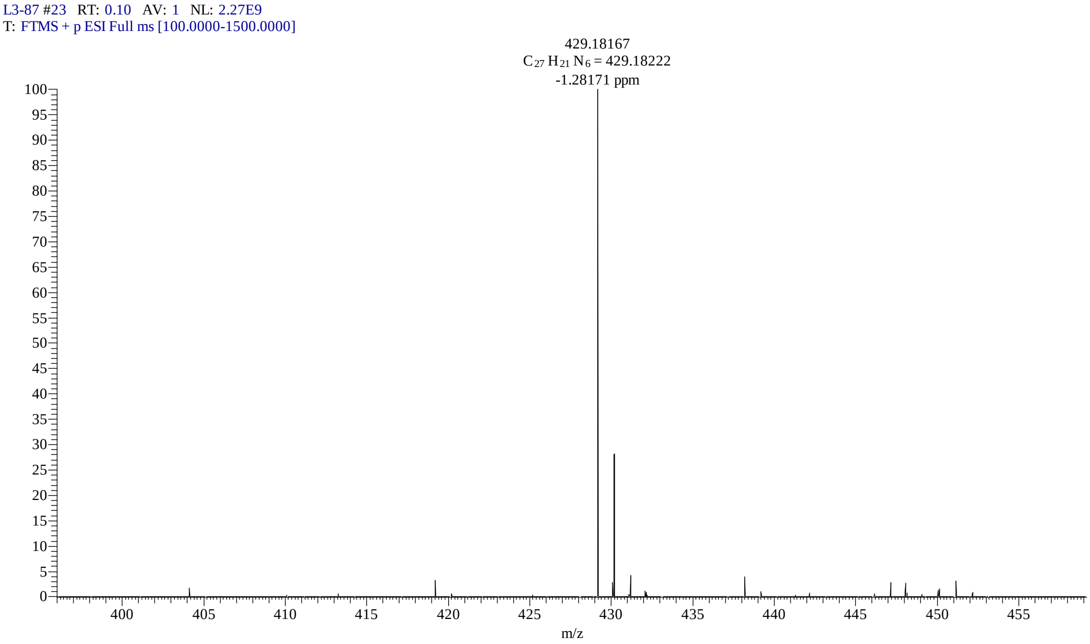

L3-87 #23 RT: 0.10 AV: 1 NL: 2.27E9
T: FTMS + p ESI Full ms [100.0000-1500.0000]
429.18167
C27 H21 N6 = 429.18222
-1.28171 ppm
m/z

Spectra of compound **13k** (^{1}H NMR, ^{13}C NMR, HRMS)

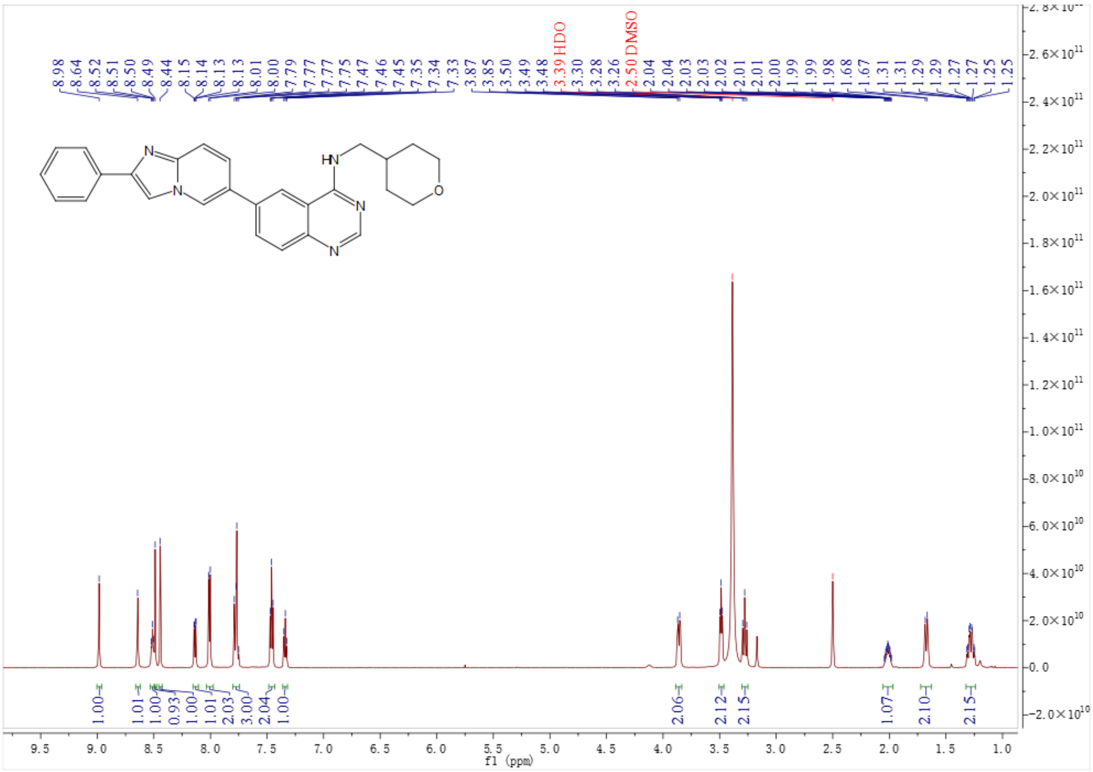

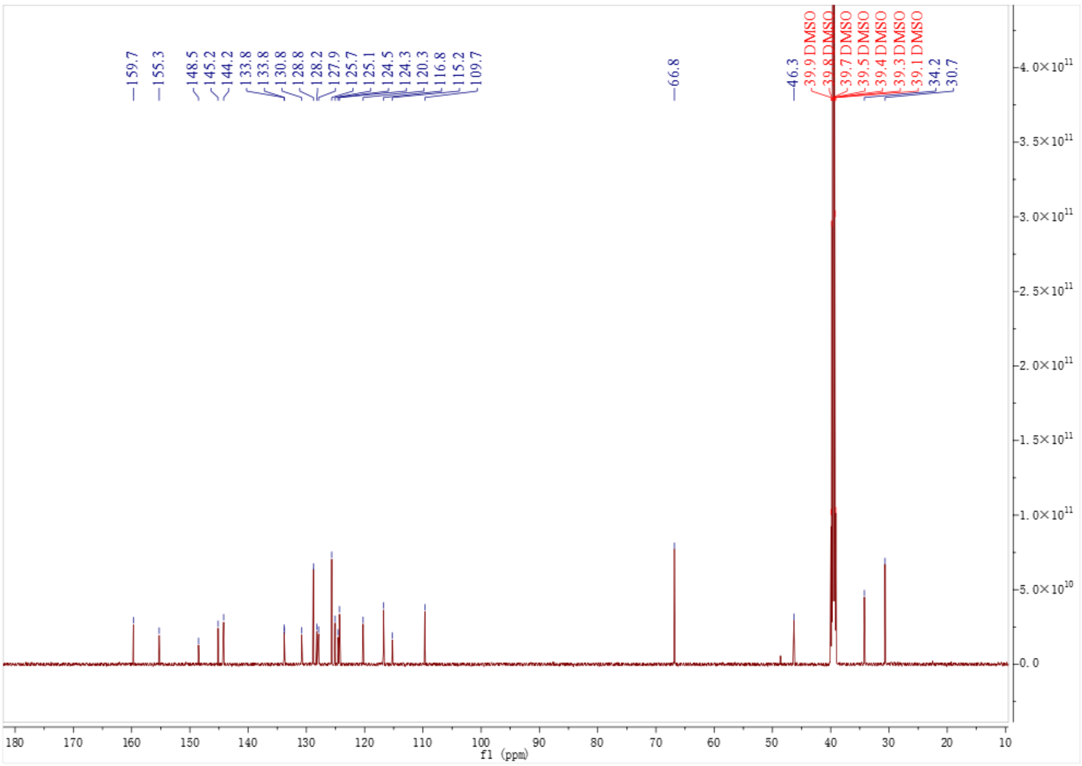

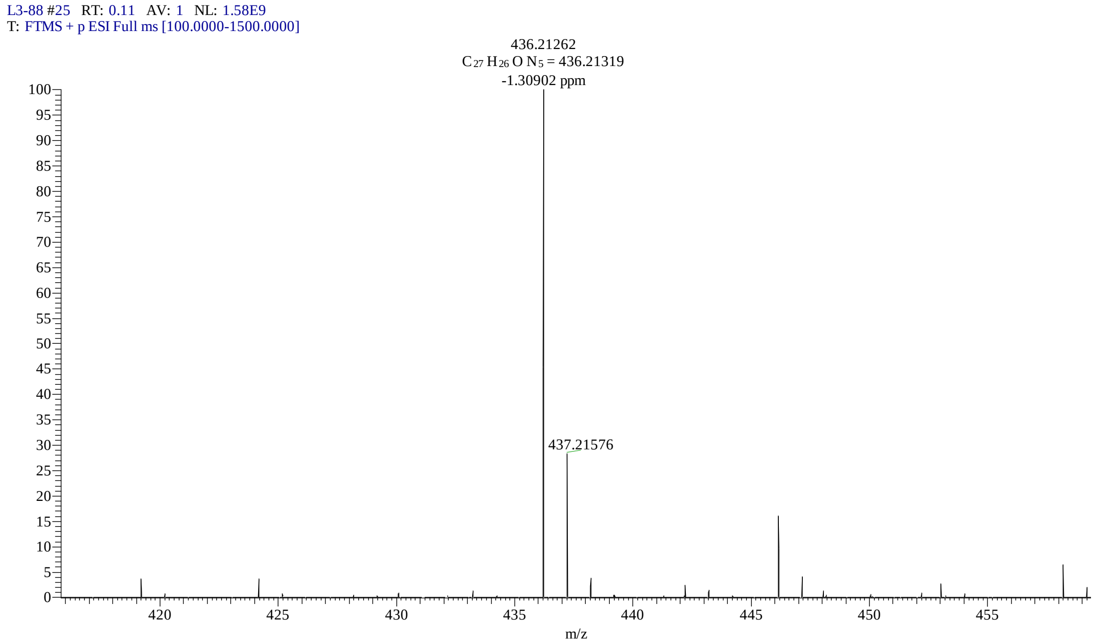

L3-88 #25 RT: 0.11 AV: 1 NL: 1.58E9
T: FTMS + p ESI Full ms [100.0000-1500.0000]
436.21262
C27 H26 O N5 = 436.21319
-1.30902 ppm
437.21576
m/z
